# Natural Genetic Variation for Growth and Development Revealed by High-Throughput Phenotyping in *Arabidopsis thaliana*

**DOI:** 10.1534/g3.111.001487

**Published:** 2012-01-01

**Authors:** Xu Zhang, Ronald J. Hause, Justin O. Borevitz

**Affiliations:** * Department of Ecology and Evolution; † Ben May Department for Cancer Research; ‡ Committee on Genetics, Genomics, and Systems Biology, University of Chicago, Chicago, Illinois 60637

**Keywords:** high throughput phenotyping, time-lapse image analysis, growth, development, developmental heterogeneity

## Abstract

Leaf growth and development determines a plant’s capacity for photosynthesis and carbon fixation. These morphological traits are the integration of genetic and environmental factors through time. Yet fine dissection of the developmental genetic basis of leaf expansion throughout a growing season is difficult, due to the complexity of the trait and the need for real time measurement. In this study, we developed a time-lapse image analysis approach, which traces leaf expansion under seasonal light variation. Three growth traits, rosette leaf area, circular area, and their ratio as compactness, were measured and normalized on a linear timescale to control for developmental heterogeneity. We found high heritability for all growth traits that changed over time. Our study highlights a cost-effective, high-throughput phenotyping approach that facilitates the dissection of genetic basis of plant shoot growth and development under dynamic environmental conditions.

Whole-genome studies that aim to reveal the genetic basis of phenotypic traits have been greatly facilitated by technological advances in genotyping and sequencing. These studies, however, typically depend on phenotyping procedures that are labor intensive. In addition, many interesting biological processes are currently not suitable for genetic mapping due to a lack of approach to efficiently and reliably record the trait. For -omic phenotypes such as metabolite accumulation, parallel measurement of many traits is often possible by utilizing specific instruments (Fiehn *et al.* 2001; Sawada *et al.* 2009). For morphological traits, automatic phenotyping approaches are needed to quantify trait value through time. Recent approaches have applied imaging tracking systems to record, for example, root (Miller *et al.* 2010) and hypocotyl (Cole *et al.* 2011) dynamics, seedling growth (Walter *et al.* 2007), and pathogen resistance (Berger *et al.* 2007).

Growth is an environmentally sensitive trait interconnecting cell biology, organogenesis, and physiology. In Arabidopsis, leaf growth occurs primarily at the rosette stage. Leaf primordia initiate in a sequential manner, upon which leaf blade and tissue layers establish (Donnelly *et al.* 1999). Across development, leaf growth is coordinated with other life history events, through modulating the activity of the shoot apical meristem (Cookson *et al.* 2007). Growth traits are highly plastic, affected by a broad range of internal and external signals (Ben-Haj-Salah and Tardieu 1995; Massonnet *et al.* 2010; Pereyra-Irujo *et al.* 2008; Werner *et al.* 2003). Leaf area expansion, a proxy of leaf growth that can be measured using a noninvasive image analysis approach (Arvidsson *et al.* 2011; El-Lithy *et al.* 2004; Granier *et al.* 2006), was shown to be controlled by significant genetic factors (El-Lithy *et al.* 2004; Massonnet *et al.* 2010; Perez-Perez *et al.* 2002; Tisne *et al.* 2008).

Previous approaches to high-throughput phenotyping of leaf growth often focus on one plant at a time and are relatively expensive (Arvidsson *et al.* 2011; El-Lithy *et al.* 2004; Granier *et al.* 2006). These systems often depend on robotic arms or conveyor belts with cameras and lighting to sequentially photograph target plants. Alignment of sequential images can be an issue, and there are structural limitations on lighting and growing conditions. For an ordinary laboratory, the cost of the hardware is prohibitive. In this study, we present an image analysis approach that is cost effective, allows seasonally variable growth chamber settings, and features real-time data acquisition and phenotype processing. We implement this high-throughput phenotyping pipeline in an initial attempt to finely map common growth QTL throughout development and across simulated climates.

## Materials and Methods

### Plant growth setting

The plant growth conditions were reported in our previous study (Li *et al.* 2010). In the experiment, the chambers simulated environments for two locations (Spain and Sweden) by two seasons (spring and summer). For each of the four environments, 144 accessions were divided into four flats placed on two shelves. Due to data quality, 57 accessions from the top shelf of Spain spring and 58 accessions from the bottom shelf of Spain summer were used for genome-wide association studies (GWAS). An additional five training accessions with five replicates were grown on the top shelf for each of Spain spring and Sweden spring conditions.

### Imaging system

Canon SD870 cameras were mounted below fluorescent lights using a tripod and powered by an AC adaptor. During the nighttime, images were taken under illumination by green LED light strips to avoid interference with light-regulated development. The Eye-Fi wireless SD card was used to transmit the images to a Linux server. The open source CHDK firmware provides full programming control of the camera via UBASIC scripts, which permits the user to hardcode exposure and focus, to disable the flash, to define a custom color space for improving green color sampling, and to acquire time-lapse images. For the operation of our system, special aperture conditions were adjusted for the day (ultra intervalometer) and night (long exposure intervalometer).

### Image analysis pipeline

The image analysis pipeline consists of several steps.

#### 1) Preprocessing of the images

To determine the appropriate timeframe for phenotyping, pictures of each flat were examined in time order. The analysis was restricted to the timeframe from the date when the plants were thinned to one plant per pot to the date when the first plant bolted within the flat. The picturing time was extracted by “exiftool” in Perl. The positional coordinates of individual plants within the flat were determined using the “locator” function in R. A flat may have multiple coordinate files due to slight movement of the flat during the experiment. An Excel spreadsheet was generated for each flat, recording the file names of the images in time order across the phenotyping timeframe, and the corresponding coordinate files. Corrupted pictures were also marked out on the spreadsheet.

#### 2) Separation of daytime pictures from nighttime picture

As the signal- to-noise ratio for nighttime pictures was low, we removed nighttime pictures from further analysis. For each flat, the images were filtered by subtracting the red channel from the green channel. The mean filtered intensities of images were plotted against time to empirically determine the cutoff between daytime and nighttime images.

#### 3) Make image stacks for individual plants

The images for each flat were cropped according to positional coordinates so that each cropped image contains a single plant. An image stack in time order was generated for each plant and matched to the corresponding accession name. From then on, analysis was carried out on the image stacks.

#### 4) Color filtering

The color filter is a linear combination of pixel intensities from the red, green, and blue channel

$$D_{\mathrm{ijk}}=0.35\times (50000-A_{\mathrm{ik}})/50000$$

$$F_{\mathrm{ijk}}=(G_{\mathrm{ijk}}-R_{\mathrm{ijk}}+0.4)\times (1-D_{\mathrm{ijk}})+(G_{\mathrm{ijk}}-{\mathrm{RB}}_{\mathrm{ijk}}+0.4)\times D_{\mathrm{ijk}}$$

Where the subscripts represent the i^th^ plant at the j^th^ time point in the k^th^ day. A_ik_ denotes the rosette area for the i^th^ plant at noontime in the k^th^ day. D_ijk_ is a weighting factor. As blue pot edges were easily blended in the foreground pixels when plants were small, D_ijk_ is determined so that a proportion of blue channel is subtracted depending on plant size. F_ijk_ is the filtering function, with G_ijk_, R_ijk_, and RB_ijk_ representing the pixel intensities of the green channel, red channel, and the weighted mean across red and blue channels, respectively. Further image analysis was applied on the filtered pixel intensities.

#### 5) Estimate background threshold

The background threshold of an image is estimated by

$$T_{\mathrm{ijk}}=C_{s}(s=\text{spring | summer})+0.470xU_{\mathrm{ijk}}-0.00000146\times A_{\mathrm{ik}}$$

where the subscripts i, j, and k were described above. C_s_ is a season-dependent constant with 0.335 for spring and 0.305 for summer. U is the mean pixel intensity of the filtered image. A_ik_ is the rosette area of the i^th^ plant at the noontime in k^th^ day.

#### 6) Rosette detection

The images of the i^th^ plant in the k^th^ day were read as JPG files. The noontime image, which generally has the highest mean (green − red) channel intensity, was selected for analysis to estimate the rosette area A_ik_. The functions for color filtering and background threshold estimation are similar to that described above, with slight modifications:

$$D_{\mathrm{ik}}=0.35\times (50000-A_{i(k-1)})/50000$$

$$F_{\text{ik}}=(G_{\mathrm{ik}}-R_{\mathrm{ik}})\times (1-D_{\mathrm{ik}})+(G_{\mathrm{ik}}-{\mathrm{RB}}_{\mathrm{ik}})\times D_{\mathrm{ik}}$$

$$T_{\mathrm{ik}}=0.12+0.475\times U_{\mathrm{ik}}-0.00000130\times A_{i(k-1)}$$

Where A_i(k−1)_ is the rosette area at the noontime in the (k-1)^th^ day, with A_i1_ set to 1000 for the beginning day. Pixels with intensity < T_ik_ were set to 0 as background. A distance map was calculated for the foreground pixels (R::EBImage::diatmap), and a watershed function was applied to index the foreground objects with tolerance = 1 and extension = 3 (R::EBImage::watershed). Hull features were calculated for the indexed objects (R::EBImage::hullFeatures). Those objects with surface area or perimeter < N, or surface area / perimeter < 1.5, were removed as noise objects. Here, N = 30 if A_i(k−1)_ ≤ 20,000; otherwise, N = 60. The remaining objects were combined into one object, for which surface area A_ik_ was calculated. All images for the i^th^ plant in the k^th^ day were then analyzed with A_ik_ introduced. Edge profiles were also calculated (R::EBImage::edgeFeatures) to determine the radius of the rosette.

The estimation of model parameters took several steps. We started by working on a training flat with five accessions by five replicates grown in Spain spring condition. Images were color filtered by directly subtracting red channel from green channel. In general, there was a relatively sharp separation between foreground and background pixel distributions for noontime images. We then estimated parameters of the threshold function for noon images, ${\text{T}}_{\text{ik}}{=\text{C1 - C3 x A}}_{\text{i(k−1)}}$. C1 was estimated empirically by examining the pixel distributions (see supporting information, Figure S2A) across plants when they had ∼six true leaves. C3 is the parameter of rosette area A_i(k−1)_, which describes across-day growth variation of the focal plant. C3 was estimated by probing an empirically defined parameter space while fixing C1 across developmental stages of the training plants. In the general threshold function ${\text{T}}_{\text{ijk}}{=\text{C1+C2 x U}}_{\text{ijk}}{\text{− C3 × A}}_{\text{ik}}$, C2 is the parameter of the mean filtered pixel intensity U_ijk_, which describes daily chamber light variation. Images for the training plants across time points on the date of ∼six true leaves were explored to obtain an initial estimation of C2 by fixing C1 and C3. Adjustment of the parameters was carried out across all training plants, through developmental stages, and across seasonal conditions. As there is no closed-form estimation of model parameters, the fitness of the models was solely determined by comparing the original image with the masked image (see Figure S1). Analysis scripts are available at http://borevitzlab.uchicago.edu/resources/imaging.

### Genetic analysis and genome-wide association mapping

For genetic analysis, heritability of traits was estimated with a mixed-effects model with genotype as random effect.

## Results

### Time-lapse image analysis

We developed a simple, cost effective imaging system that can be readily adjusted to different growth chambers. We utilized commercial Canon point-and-shoot cameras (PowerShot S series) with Canon Hacker Development Kit (CHDK) open source firmware (http://chdk.wikia.com) to capture time-lapse images. Each picture captured up to 36 plants grown singly in a 6- × 6-pot flat. The top view of plants were imaged every 20 min. Images were transferred wirelessly through Eye-Fi SD cards (http://www.eye.fi) to a Gallery server, where each camera/flat had a unique folder. Within each folder, images were cropped to contain single plants according to the positional coordinates of pots within the flat and stacked in temporal order per plant.

Image analysis was carried out stack-wise using functions of the R EBImage package (Sklyar and Huber 2006). For each image, a distance map was calculated for foreground pixels. Based on the intensity and the relative position of foreground pixels, objects were detected and indexed by a watershed algorithm. Background noise, or false objects, were determined by their geometric property and removed. The remaining objects (rosette leaves) were combined into one object (rosette), on which hull features and edge profiles were calculated to obtain rosette area and radius.

**Figure 1:**
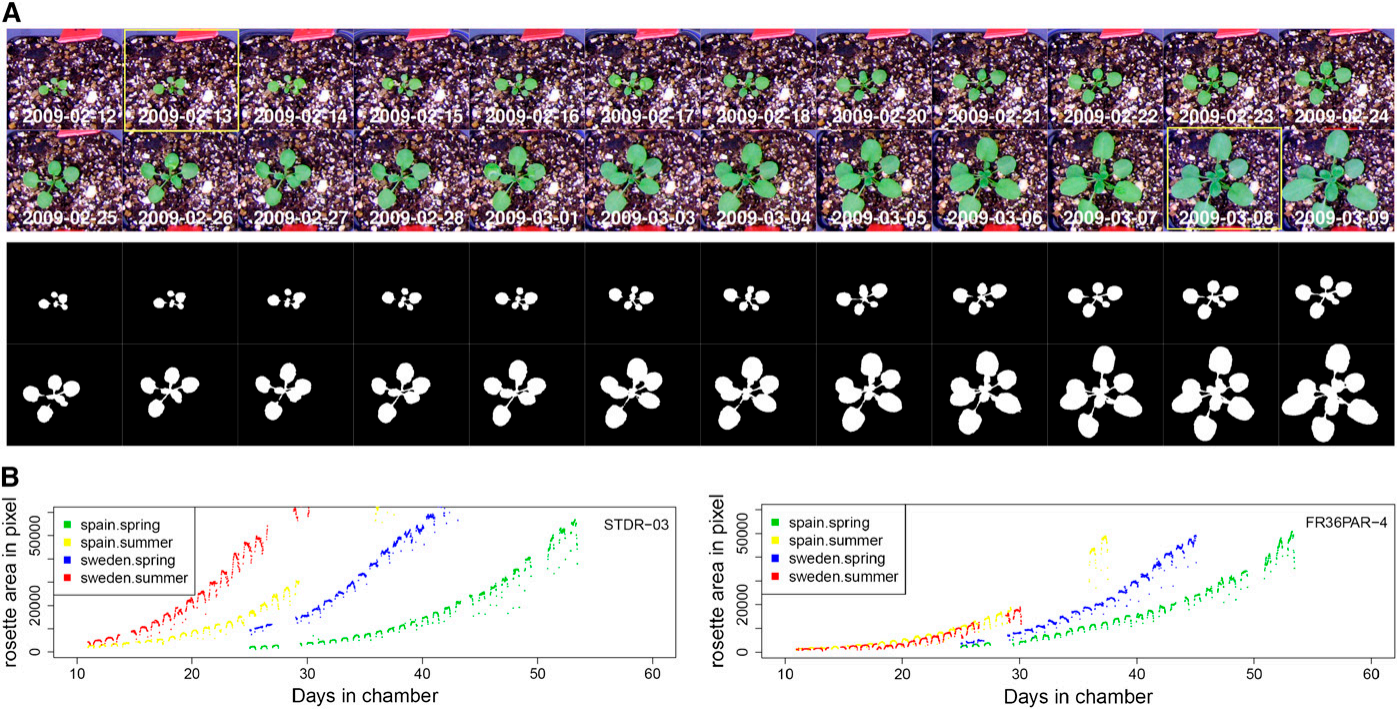
Detection of rosette area. (A) Detection of rosettes for images taken at 16:20:00 across days. The original images (upper) and the detected rosettes (lower). The boxed images represent plant at stage 1.04 and 1.10, respectively. (B) Rosette areas for accession STDR-03 (left) and FR36PAR-4 (right) were plotted against the number of days plants were grown in chambers, for Spain spring (green), Spain summer (yellow), Sweden spring (blue), and Sweden summer (red). Images were taken at 20 min intervals in daytime. Vacant points were due to missing data.

Separation of foreground pixels from background pixels is a critical step in rosette detection. We found that a mixture model, which selects foreground pixels based on the mixture distribution of RGB channel intensity, did not work well due to biased sampling (data not shown). As plants were grown in a common setting, particles of perlite and vermiculite in the soil as well as the edges of pots strongly reflected light. To increase the detection specificity for green pixels, we applied a color filter to the images (Figure S1). A background threshold was then estimated. For our experiment, this threshold was a dynamic parameter due to several factors. First, the light intensity and spectrum of our diurnal and season-simulating chambers varies within a day and across days. Second, the amount and spectrum of light reflected from a rosette vary as a plant grows (Figure S2). Third, individual plants reflect light differently due to their position under the camera. To accommodate light, growth, and individual positioning variation, we developed a hierarchical linear model for estimation of a background threshold (see *Materials and Methods*). Integrating this step into our analysis pipeline allows for the automatic processing of image stacks (Figure 1A, Figure S3, and movie in File S1).

We quantified leaf area during rosette development for plants grown in four simulated seasonal conditions with ∼144 accessions per condition. Rosette growth pattern exhibits large variation across seasonal conditions (Figure 1B). In spring, for example, growth in cooler Spain in March conditions tends to be delayed compared with growth in warmer Sweden in May conditions (Figure S4). And growth in summer is generally faster than in spring. There is also substantial variation among accessions, implying potential genetic effects for rosette growth (Figure 1B). Within a day, leaf area peaks around noon when rosette leaves lie flat. Area is slightly reduced at dawn and dusk when leaves move upwards (Figure 1B). Two confounding effects, the diurnal movement of leaves and the reduced rosette area detection power under simulated twilight, contribute to this pattern, which was not further analyzed in this study.

### Genotype and environmental effects on rosette growth and development

At any given time point, the precise developmental stage among plants grown under the same environmental condition is heterogeneous. As rosette area variation is the combinatory variation of leaf initiation (development) and leaf expansion (growth), a clear definition of growth trait requires a separation of growth from development. To normalize the developmental scale for genetic analysis, we defined rosette developmental stages (Boyes *et al.* 2001) according to images taken at noon. Reliable rosette area estimation could only be obtained for the interval between when the seedlings were thinned to a single plant per pot and when they grew beyond the pot edges. Therefore, we focused on the developmental timeframe between the beginning of the day, when the fourth true leaf was 10 pixels long (approximately, stage 1.04) and the end of the day, when the tenth true leaf was about 10 pixels long (stage 1.10).

We first examined the developmental stage variation for a training set, which included five accessions each with five replicates grown in a simulated Spain spring condition. Here we denoted the amount of time it takes for plants to reach stage 1.04 as T1.04 and the time to reach stage 1.10 as T1.10. Genetic variation explains a moderate proportion of the total variation for T1.04 (*H^2^* = 0.46) and for T1.10 (*H^2^* = 0.47). However, 88% of the variation for T1.10 can be explained by T1.04 (Figure S5A), suggesting that developmental variation is mainly due to heterogeneity at the initial stage. In addition, leaf initiations were relatively synchronized across genotypes by controlling T1.04 variation (Figure S5B). This suggests the feasibility of linear scaling to normalize developmental variation across plants (Turnbull *et al.* 2008). For each plant, we scaled stages 1.04 through 1.10 to a relative developmental timeframe of 0 through 1. Relative time at absolute time Ti was calculated as (Ti − T1.04) / (T1.10 – T1.04), which puts each plant on the same developmental timescale.

**Figure 2:**
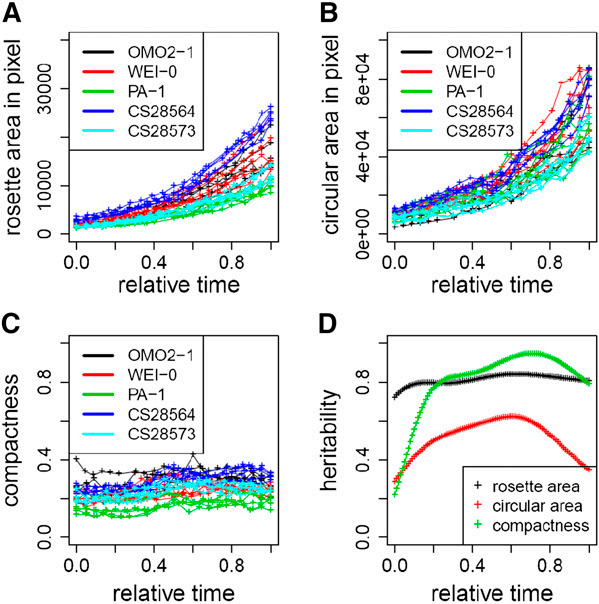
Genetic effect for rosette growth-related traits. Rosette area (A), circular area (B), and compactness (C) were plotted against relative growth time. Data points were obtained from noontime images across developmental stage 1.04 through 1.10 for each plant. Colors denote different accessions. (D) The broad sense heritability for rosette area (black), circular area (red), and compactness (green) was plotted against relative growth time. Heritability was calculated on spline-fitted data points.

We measured several growth-related traits on the pixels detected as rosette leaves. Rosette area (RA) was measured as the sum of these pixels. Radius was measured as the maximum distance between the geometric center of rosette to the rosette edge, and circular area (CA) was calculated with this radius. Compactness was defined as the ratio of RA to CA, the proportion of a filled circle. For each plant, a spline of RA, CA, or compactness against absolute time was fitted from stage 1.04 through 1.10. The growth curves were then scaled as described above (Figure S6). We estimated the genetic heritability of these traits for the training set. Across the developmental timeframe, there is a large component of genetic variation among accessions for these traits (Figure 2). The heritability of RA is relatively constant, with a median of 0.81. The heritability of CA peaks at 0.62 around the middle of developmental time and has a median of 0.53. The heritability of compactness peaks at 0.95 in late developmental time, with a median of 0.85. RA and CA are correlated traits, with an *r* ranging between 0.64 and 0.80 (Figure S7).

To reveal the environmental dependency of these traits, we grew the same training set under a simulated Sweden spring environment and compared it with the Spain spring condition. In general, plants grew faster and flowered with fewer leaves in Sweden spring compared with those in Spain spring. We compared traits measured when the fifth, sixth, and seventh true leaves were ∼10 pixels long (corresponding to 1.05, 1.06, and 1.07 stage, respectively) between the two environmental conditions (Figure S8). To estimate the source variance attributable to environment, development, genotype, and interaction effects, we partitioned the total variance for each trait by a multifactorial ANOVA (Table 1). Although environment has a dramatic effect on rosette area in absolute time (Figure 1B), the environmental contrast explains a small amount of variance of the normalized rosette area. As expected, development has large effect on rosette area as plants grow. Importantly, genotype also has a large main effect for these normalized traits. The interaction terms are particularly interesting. The genotype by development interaction explains 6% for RA and 4% for CA, indicating genotypes change growth differently through development. Genetic variation for compactness was largely unaffected by development. However, environment did change to some extent the way genotypes fill in their circular area. Consistent with this, petiole elongation and leaf blade expansion are temperature- and light-sensitive traits (Reed *et al.* 1993).

**Table 1:** Variance partitioning of environment, development, genotype, and interaction effects in the training set

	*P*			% Variance		
	RA	CA	Compactness	RA	CA	Compactness
Environment (Env)	1.3 × 10^−02^	NS	1.6 × 10^−03^	0.8%	0.2%	2.6%
Development (Dev)	2.1 × 10^−35^	5.1 × 10^−23^	9.5 × 10^−12^	42.6%	37.1%	15.8%
Genotype (Geno)	2.1 × 10^−29^	8.4 × 10^−17^	1.7 × 10^−23^	33.6%	26.4%	46.5%
Env:Dev	NS	NS	NS	0.2%	0.8%	0.8%
Env:Geno	NS	NS	3.9 × 10^−02^	0.4%	1.8%	2.6%
Dev:Geno	1.7 × 10^−06^	2.3 × 10^−02^	NS	6.1%	4.3%	0.8%
Env:Dev:Geno	NS	NS	NS	0.9%	2.1%	0.8%

RA, CA, and compactness were each analyzed by a multifactorial model: y ∼ environment+development+genotype+environment × development+environment × genotype+development × genotype+environment × development × genotype+ε.

NS, *P* value not significant at 0.05.

The identification of a substantial genetic effect among accessions in the training set gave us confidence to map the loci responsible for the variation of rosette growth. We mapped RA throughout development by GWA, using a mixed effects model that controls for kinship (pairwise genetic identity) among accessions (Kang *et al.* 2010). Due to data quality in this initial experiment, only a set of 57 accessions grown in Spain spring conditions and a different set of 58 accessions grown in Spain summer conditions could be used (File S2) for mapping. Unfortunately, this association study was underpowered, and there was only a slight enrichment of significant associations in real data over null expectation. This suggests a much larger sample size is needed for mapping the complex trait of rosette growth. Details for the GWA mapping are presented in File S3.

## Discussion

Rosette area is primarily determined by the rate of leaf emergence and expansion. The observed value of rosette area from a top view is affected by leaf twisting and overlap during growth, as well as diurnal movement. Nevertheless, rosette area measured via top-down imaging is directly related to leaf function, as this corresponds to the area where photosynthesis occurs. Although growth and development are concurrent, distinct genetic factors can underlay the two processes. We observed that plants were heterogeneous for early development, part of which may be traced back to variation in seed germination and/or seed size (Elwell *et al.* 2010; Turnbull *et al.* 2008). Interestingly, once such early heterogeneity was controlled, we found a much larger genotype effect for leaf expansion (growth) than for leaf initiation (development). Whether this is a common phenomenon needs a more comprehensive evaluation of genotypes. To focus this study, we uncoupled growth from development by scaling developmental time across plants. We detected major genetic components for three growth-related traits, RA, CA, and compactness.

In comparison with other reported approaches for high-throughput phenotyping, our imaging system requires simple camera hardware affixed in standard growth chambers. A notable advantage of our analysis approach is detecting plants under seasonally variable light conditions and across variable growth stages, which allows processing image stacks without manual intervention. Furthermore, our approach tolerates a relatively noisy background compared with others. In this study, we estimated the background thresholds of images through linear incorporation of two explanatory variables, the mean filtered light intensity and the rosette size. The linear relationship generally holds for the light conditions and plant growth stages we investigated. Refinement of modeling will lead to a unified, more robust estimation of background threshold. Phenotyping based on time-lapse imaging requires special attention in a few experimental procedures, including accurate placement of pots under the camera, allowing space between pots to avoid overlapping growth among plants, and proper pot labeling out of the camera’s view. We expect that simple imaging systems, such as the one described here, will become widely used in growth chambers, greenhouses, and ultimately field settings to quantify growth and development across contrasting environments.

## Supplementary Material

Supporting Information

## References

[bib1] Arvidsson S. Perez-Rodriguez P. Mueller-Roeber B., 2011 A growth phenotyping pipeline for Arabidopsis thaliana integrating image analysis and rosette area modeling for robust quantification of genotype effects. New Phytol. 191: 895–907 21569033 10.1111/j.1469-8137.2011.03756.x

[bib2] Ben-Haj-Salah H. Tardieu F., 1995 Temperature affects expansion rate of maize leaves without change in spatial distribution of cell length (analysis of the coordination between cell division and cell expansion). Plant Physiol. 109: 861–870 12228638 10.1104/pp.109.3.861 PMC161387

[bib3] Berger S. Sinha A. K. Roitsch T., 2007 Plant physiology meets phytopathology: plant primary metabolism and plant-pathogen interactions. J. Exp. Bot. 58: 4019–4026 18182420 10.1093/jxb/erm298

[bib4] Boyes D. C. Zayed A. M. Ascenzi R. McCaskill A. J. Hoffman N. E., 2001 Growth stage-based phenotypic analysis of Arabidopsis: a model for high throughput functional genomics in plants. Plant Cell 13: 1499–1510 11449047 10.1105/TPC.010011 PMC139543

[bib5] Cole B. Kay S. A. Chory J., 2011 Automated analysis of hypocotyl growth dynamics during shade avoidance in Arabidopsis. Plant J. 65: 991–1000 21288269 10.1111/j.1365-313X.2010.04476.x PMC3076959

[bib6] Cookson S. J. Chenu K. Granier C., 2007 Day length affects the dynamics of leaf expansion and cellular development in Arabidopsis thaliana partially through floral transition timing. Ann. Bot. (Lond.) 99: 703–711 10.1093/aob/mcm005 PMC2802938 17347163

[bib7] Donnelly P. M. Bonetta D. Tsukaya H. Dengler R. E. Dengler N. G., 1999 Cell cycling and cell enlargement in developing leaves of Arabidopsis. Dev. Biol. 215: 407–419 10545247 10.1006/dbio.1999.9443

[bib8] El-Lithy M. E. Clerkx E. J. Ruys G. J. Koornneef M. Vreugdenhil D., 2004 Quantitative trait locus analysis of growth-related traits in a new Arabidopsis recombinant inbred population. Plant Physiol. 135: 444–458 15122039 10.1104/pp.103.036822 PMC429397

[bib9] Elwell A. L. Gronwall D. S. Miller N. D. Spalding E. P. Brooks T. L., 2010 Separating parental environment from seed size effects on next generation growth and development in Arabidopsis. Plant Cell Environ. 34: 291–301 20955226 10.1111/j.1365-3040.2010.02243.x

[bib10] Fiehn O. Kloska S. Altmann T., 2001 Integrated studies on plant biology using multiparallel techniques. Curr. Opin. Biotechnol. 12: 82–86 11167078 10.1016/s0958-1669(00)00165-8

[bib11] Granier C. Aguirrezabal L. Chenu K. Cookson S. J. Dauzat M., 2006 PHENOPSIS, an automated platform for reproducible phenotyping of plant responses to soil water deficit in Arabidopsis thaliana permitted the identification of an accession with low sensitivity to soil water deficit. New Phytol. 169: 623–635 16411964 10.1111/j.1469-8137.2005.01609.x

[bib24] Kang H. M. Sul J. H. Service S. K. Zaitlen N. A. Kong S., 2010 Variance component model to account for sample structure in genome-wide association studies. Nat. Genet. 42: 348–354 20208533 10.1038/ng.548 PMC3092069

[bib12] Li Y. Huang Y. Bergelson J. Nordborg M. Borevitz J. O., 2010 Association mapping of local climate-sensitive quantitative trait loci in Arabidopsis thaliana. Proc. Natl. Acad. Sci. USA 107: 21199–21204 21078970 10.1073/pnas.1007431107 PMC3000268

[bib13] Massonnet C. Vile D. Fabre J. Hannah M. A. Caldana C., 2010 Probing the reproducibility of leaf growth and molecular phenotypes: a comparison of three Arabidopsis accessions cultivated in ten laboratories. Plant Physiol. 152: 2142–2157 20200072 10.1104/pp.109.148338 PMC2850010

[bib14] Miller N. D. Durham Brooks T. L. Assadi A. H. Spalding E. P., 2010 Detection of a gravitropism phenotype in glutamate receptor-like 3.3 mutants of Arabidopsis thaliana using machine vision and computation. Genetics 186: 585–593 20647506 10.1534/genetics.110.118711 PMC2946860

[bib15] Pereyra-Irujo G. A. Velazquez L. Lechner L. Aguirrezabal L. A., 2008 Genetic variability for leaf growth rate and duration under water deficit in sunflower: analysis of responses at cell, organ, and plant level. J. Exp. Bot. 59: 2221–2232 18448477 10.1093/jxb/ern087

[bib16] Perez-Perez J. M. Serrano-Cartagena J. Micol J. L., 2002 Genetic analysis of natural variations in the architecture of Arabidopsis thaliana vegetative leaves. Genetics 162: 893–915 12399398 10.1093/genetics/162.2.893 PMC1462278

[bib17] Reed J. W. Nagpal P. Poole D. S. Furuya M. Chory J., 1993 Mutations in the gene for the red/far-red light receptor phytochrome B alter cell elongation and physiological responses throughout Arabidopsis development. Plant Cell 5: 147–157 8453299 10.1105/tpc.5.2.147 PMC160258

[bib18] Sawada Y. Akiyama K. Sakata A. Kuwahara A. Otsuki H., 2009 Widely targeted metabolomics based on large-scale MS/MS data for elucidating metabolite accumulation patterns in plants. Plant Cell Physiol. 50: 37–47 19054808 10.1093/pcp/pcn183 PMC2638709

[bib19] Sklyar O. Huber W., 2006 Image analysis for microscopy screens. R News 6: 12–16

[bib20] Tisne S. Reymond M. Vile D. Fabre J. Dauzat M., 2008 Combined genetic and modeling approaches reveal that epidermal cell area and number in leaves are controlled by leaf and plant developmental processes in Arabidopsis. Plant Physiol. 148: 1117–1127 18701672 10.1104/pp.108.124271 PMC2556812

[bib21] Turnbull L. A. Paul-Victor C. Schmid B. Purves D. W., 2008 Growth rates, seed size, and physiology: Do small-seeded species really grow faster? Ecology 89: 1352–1363 18543628 10.1890/07-1531.1

[bib22] Walter A. Scharr H. Gilmer F. Zierer R. Nagel K. A., 2007 Dynamics of seedling growth acclimation towards altered light conditions can be quantified via GROWSCREEN: a setup and procedure designed for rapid optical phenotyping of different plant species. New Phytol. 174: 447–455 17388907 10.1111/j.1469-8137.2007.02002.x

[bib23] Werner T. Motyka V. Laucou V. Smets R. Van Onckelen H., 2003 Cytokinin-deficient transgenic Arabidopsis plants show multiple developmental alterations indicating opposite functions of cytokinins in the regulation of shoot and root meristem activity. Plant Cell 15: 2532–2550 14555694 10.1105/tpc.014928 PMC280559

