# Supplementary material for: Natural Genetic Variation for Growth and Development Revealed by High-Throughput Phenotyping in Arabidopsis thaliana
Source: G3 (Bethesda). 2012 Jan 1;2(1):29–34. doi: 10.1534/g3.111.001487 (PMC3276187; doi:10.1534/g3.111.001487)
Supplement: Supporting Information [file supp_2_1_29__index.html]

Supporting Information 

# Natural Genetic Variation for Growth and Development Revealed by High-Throughput Phenotyping in *Arabidopsis thaliana*

## Supporting Information for Zhang, Hause Jr, and Borevitz, 2012

**Files in this Data Supplement:**

- Supporting Information - Figures S1-S8 and Tables S1-S3 (PDF, 3 MB)
- Figure S1 - Detection of rosette (PDF, 392 KB)
- Figure S2 - (A) The density distribution of pixel intensity for a cropped image, from noon to dusk within a day. (B) The density distribution of pixel intensity for a cropped image, at noon time across days. (PDF, 264 KB)
- Figure S3 - The detection of rosette across different time points from sunrise to sunset within a day (PDF, 568 KB)
- Figure S4 - The differential of photothermal unit (ΔPTU) against time for simulated Spain (black) and Sweden (red) conditions (PDF, 428 KB)
- Figure S5 - (A) T1.10 is largely explained by T1.04. B) Leaf initiation was relatively synchronized once T1.04 was controlled. (PDF, 496 KB)
- Figure S6 - Fit rosette area (RA) against time by a one-knot spline for 5 accessions x 5 replicates grown in Spain spring condition (PDF, 440 KB)
- Figure S7 - Correlation between traits (PDF, 200 KB)
- Figure S8 - The rosette area (upper), circular area (middle) and compactness (lower) plotted for each genotype, across developmental stage 1.05 (black), 1.06 (red) and 1.07 (green), under Spain spring (cross points) and Sweden spring (diamond points) conditions (PDF, 308 KB)
- File S2 - Rosette Area Data Summary (PDF, 3.2 MB)
- File S3 - GWA Mapping of Rosette Area (PDF, 428 KB)
- File S1 - Supporting Movie (.zip, 23.6 MB)
