# Supplementary material for: Natural Genetic Variation for Growth and Development Revealed by High-Throughput Phenotyping in Arabidopsis thaliana
Source: G3 (Bethesda). 2012 Jan 1;2(1):29–34. doi: 10.1534/g3.111.001487 (PMC3276187; doi:10.1534/g3.111.001487)
Supplement: Supporting Information [file supp_2.1.29_001487SI.pdf]

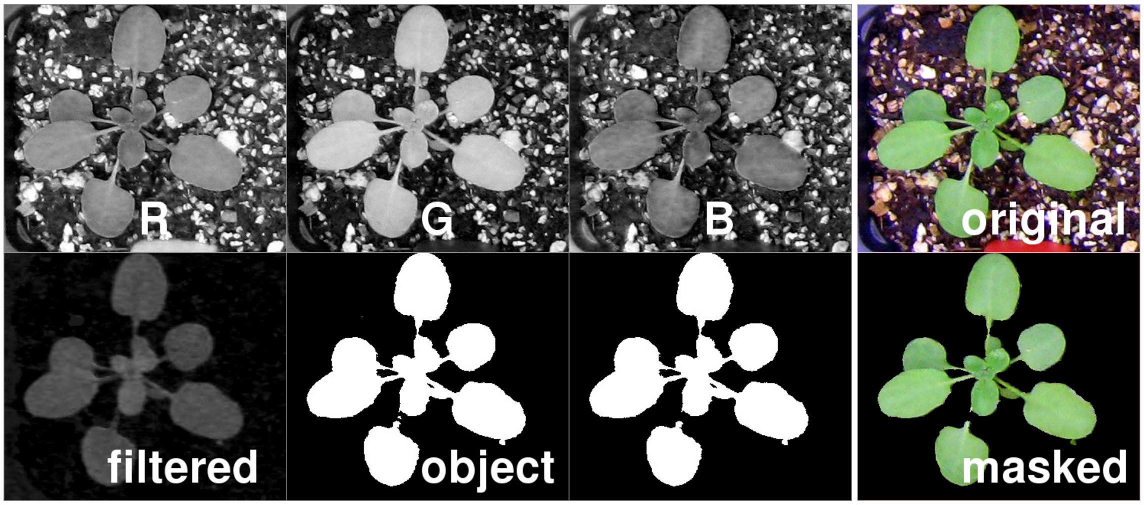

Figure S1 Detection of rosette. From left to right of the upper panel were red, green and blue channel, and the original image. From left to right of the lower panel were color-filtered image, image containing detected objects, image with noise objects removed, and the original image with background and noise objects removed.

A

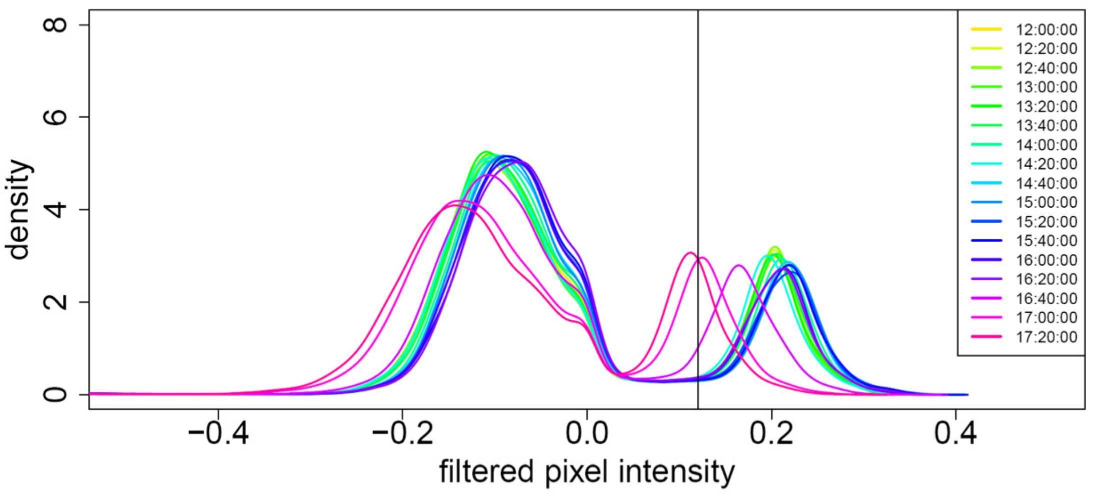

B

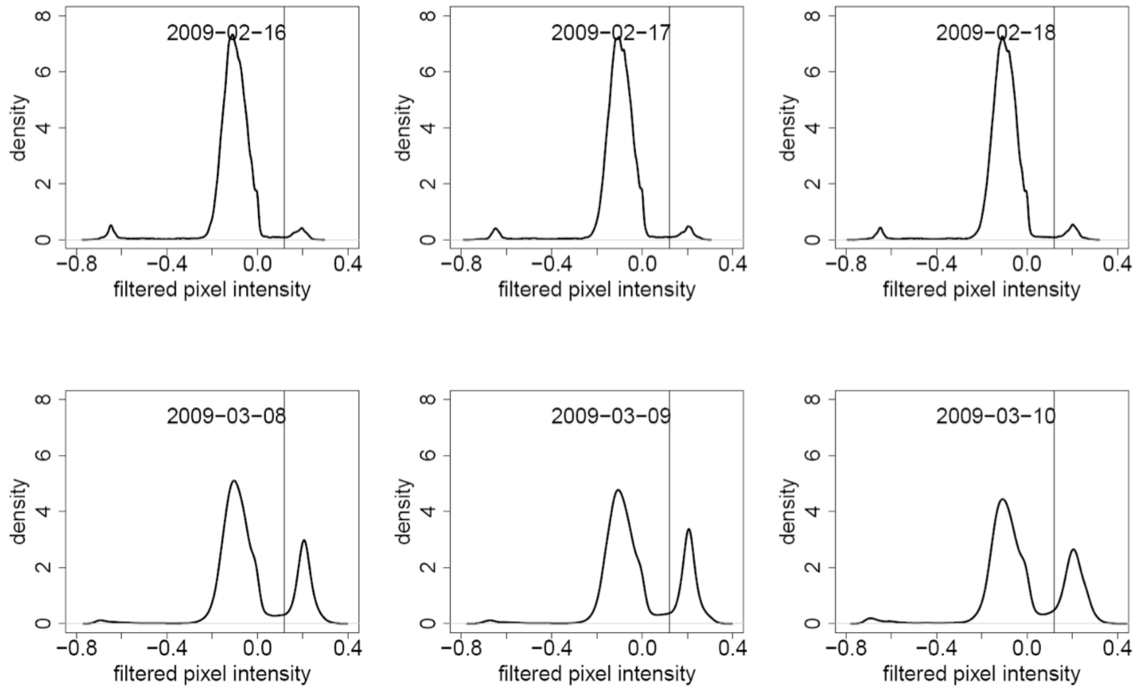

Figure S2 (A) The density distribution of pixel intensity for a cropped image, from noon to dusk within a day. The color filter applied here was green channel intensity – red channel intensity for simplification, thus the right-side peaks represent rosette pixels. A threshold of 0.12 (vertical line) is appropriate for noon but too high approaching sunset. **(B)** The density distribution of pixel intensity for a cropped image, at noon time across days. The color filter applied here was green channel intensity – red channel intensity for simplification, thus the right-side peaks represent rosette pixels. A threshold of 0.12 (vertical line) is appropriate when the rosette is small but too high as rosette grows.

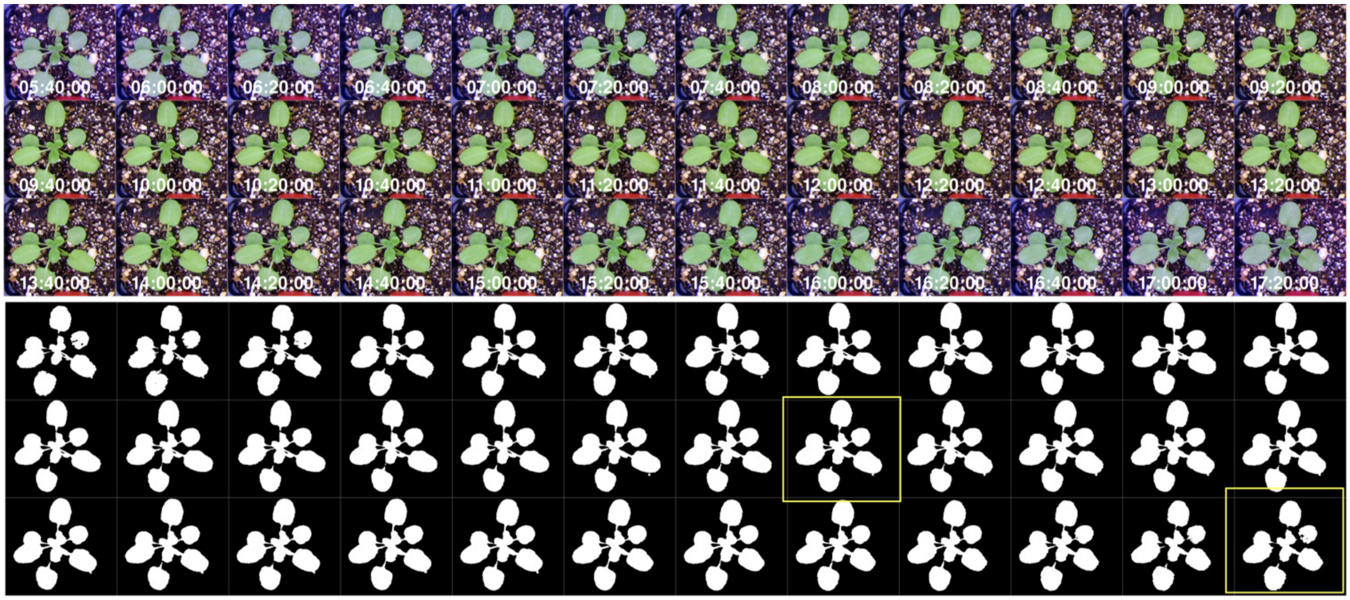

Figure S3 The detection of rosette across different time points from sunrise to sunset within a day. Note that rosette at noontime (12:00:00) is relatively extended than that toward dusk (17:20:00).

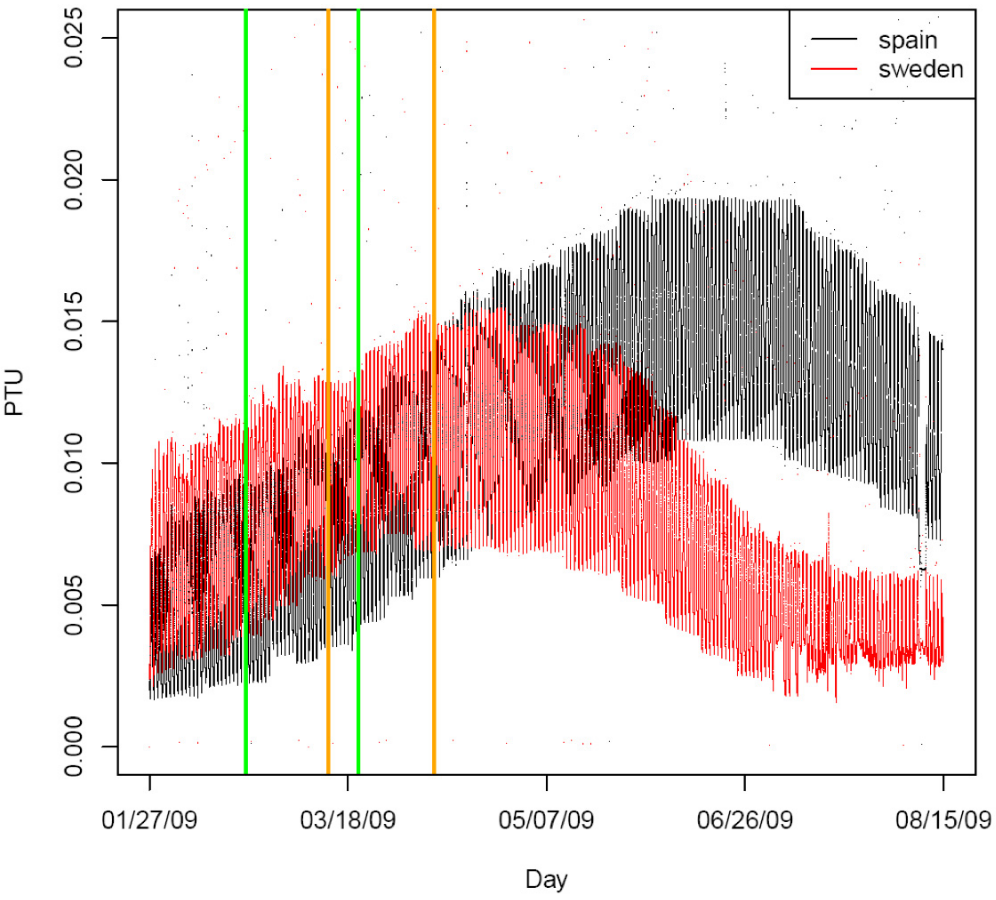

Figure S4 The differential of photothermal unit (ΔPTU) against time for simulated Spain (black) and Sweden (red) conditions. The interval between green lines represent the time interval analyzed for spring conditions, that between orange lines represent the time interval analyzed for summer conditions. ΔPTU was calculated according to Wilczek et al. 2009, for accession Col-0.

Reference: Wilczek AM, Roe JL, Knapp MC, Cooper MD, Lopez-Gallego C, Martin LJ, Muir CD, Sim S, Walker A, Anderson J, Egan JF, Moyers BT, Petipas R, Giakountis A, Charbit E, Coupland G, Welch SM and Schmitt J. 2009. Effects of genetic perturbation on seasonal life history plasticity. Science 323 (5916): 930-934.

A

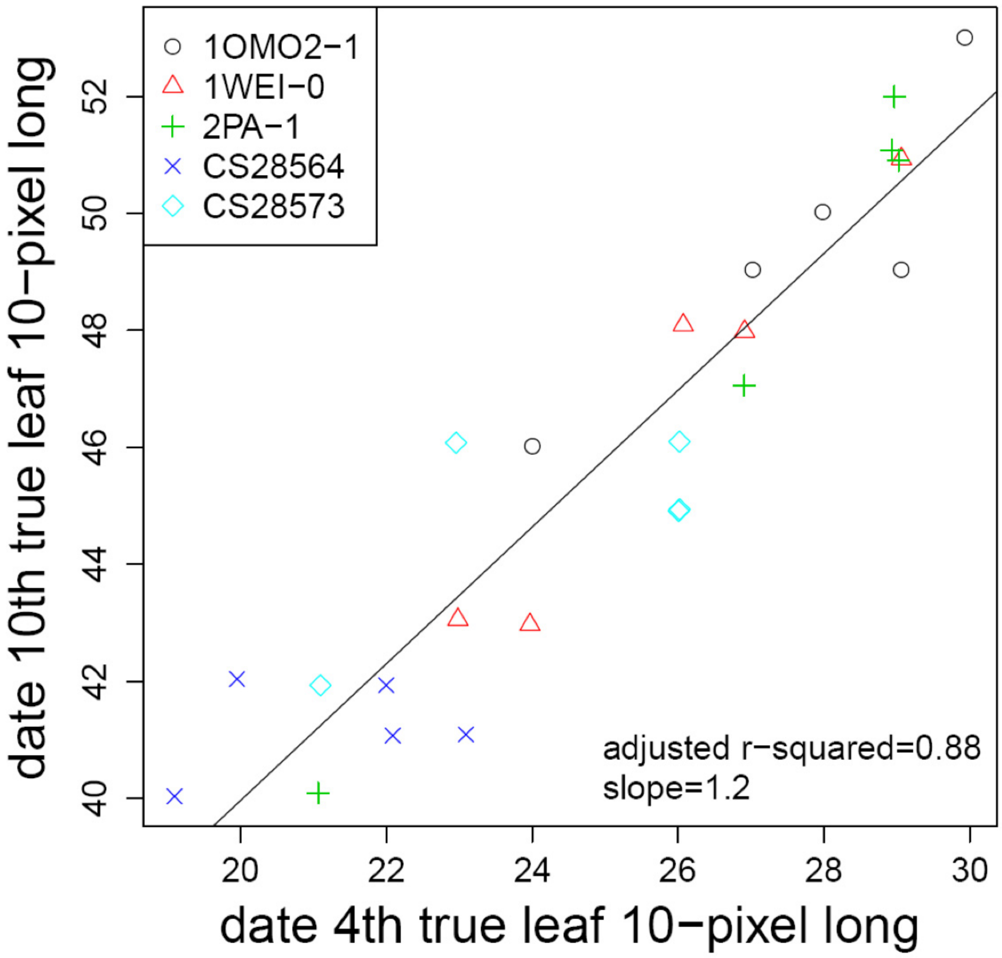
1OMO2−1
1WEI−0
2PA−1
CS28564
CS28573
adjusted r−squared=0.88
slope=1.2
date 10th true leaf 10−pixel long
date 4th true leaf 10−pixel long
40
42
44
46
48
50
52
20
22
24
26
28
30

B

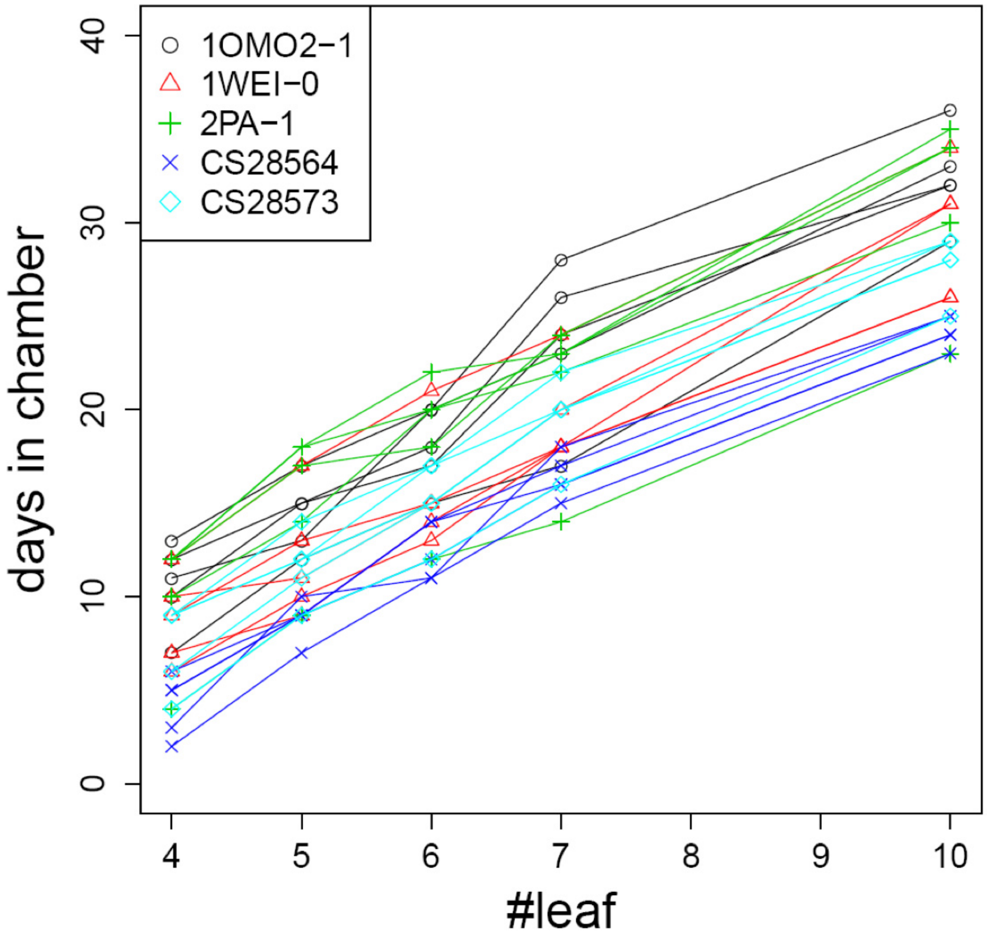

Figure S5 (A) . T1.10 is largely explained by T1.04. T1.10 was plotted against T1.04 for each plant. Colors denoted different accessions. The data points were jittered slightly on both x-axis and y-axis to avoid overlap. The adjusted r^2 is 0.88 for the model: T1.10 ~ T1.04 + ε.

(B) Leaf initiation was relatively synchronized once T1.04 was controlled. The number of days plant grown in chamber was plotted against the rosette developmental stage (when a specific leaf is 10-pixel in length). Data were analyzed by a linear model:

Ti (i > 1.04) ~ T1.04 + development + genotype + development x genotype + ε

Where development is a four-level factor including the 1.05, 1.06, 1.07 and 1.10 stages. Variance partitioning of T.1.04 (20%), development (76%), genotype (0.3%), genotype x development (0.6%).

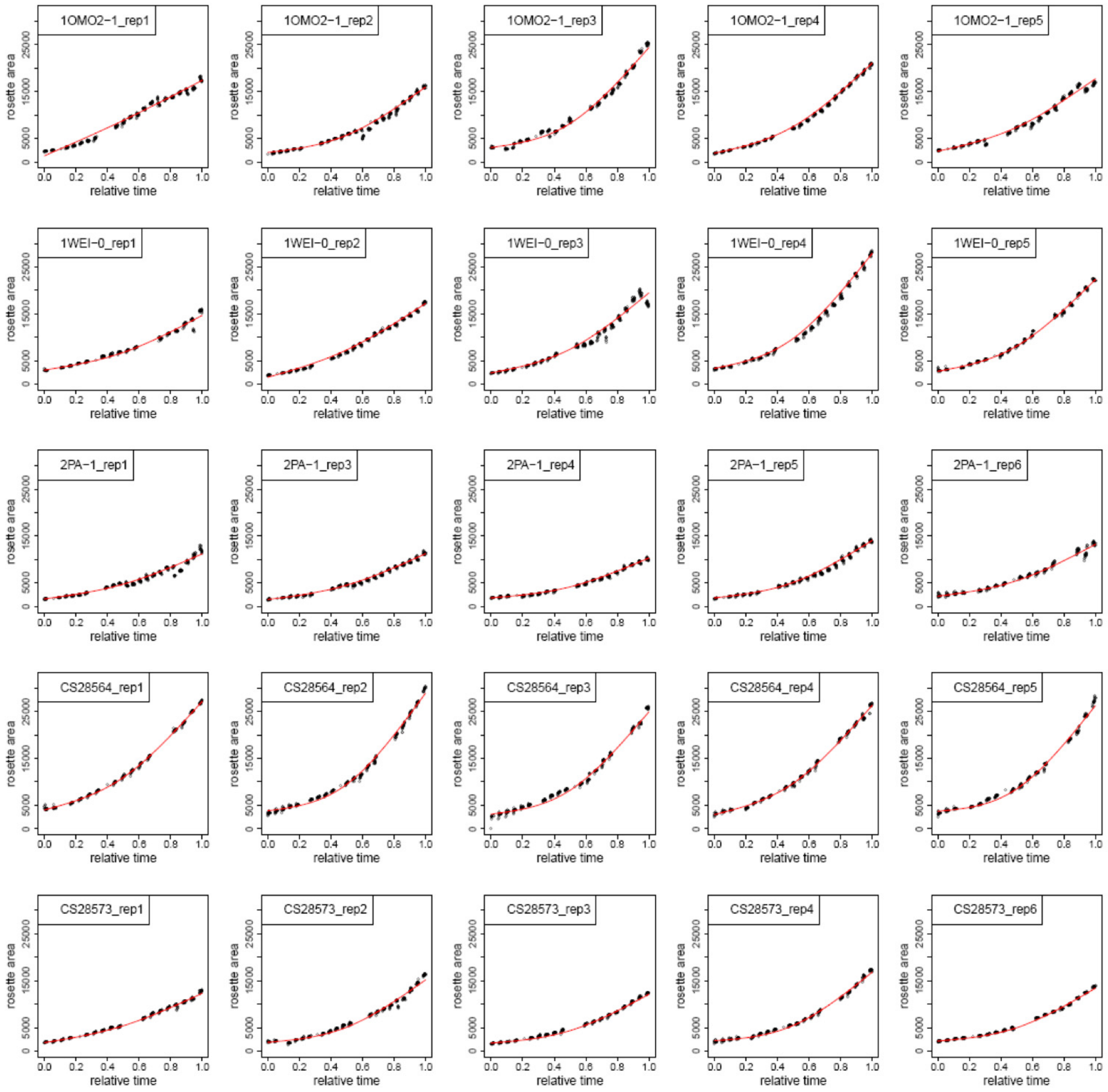

Figure S6 Fit rosette area (RA) against time by a one-knot spline for 5 accessions x 5 replicates grown in Spain spring condition. Cropped images taken from 8:00AM of the day at stage 1.04 to 4:00PM of the day at stage 1.10 were analyzed.

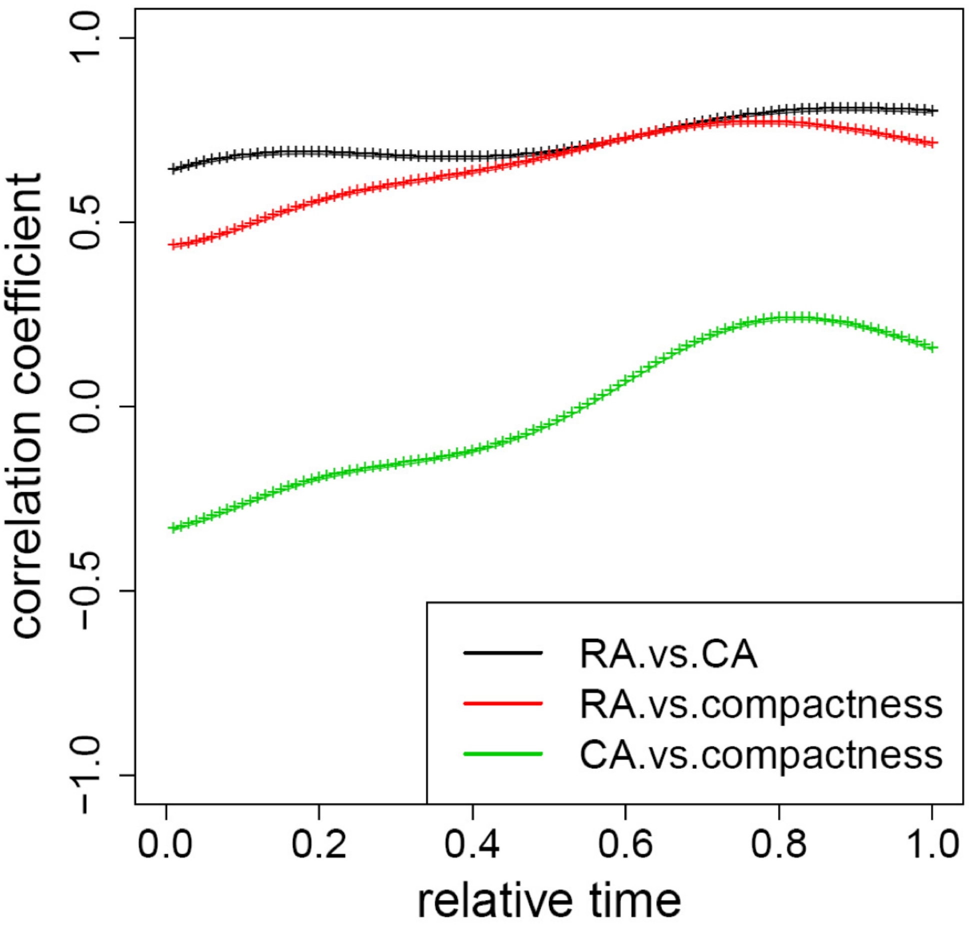

Figure S7 Correlation between traits. The correlation coefficients were plotted against relative developmental time for RA verse CA (black), RA verse compactness (red), and CA verse compactness (green). The correlation was calculated on spline-fitted data points.

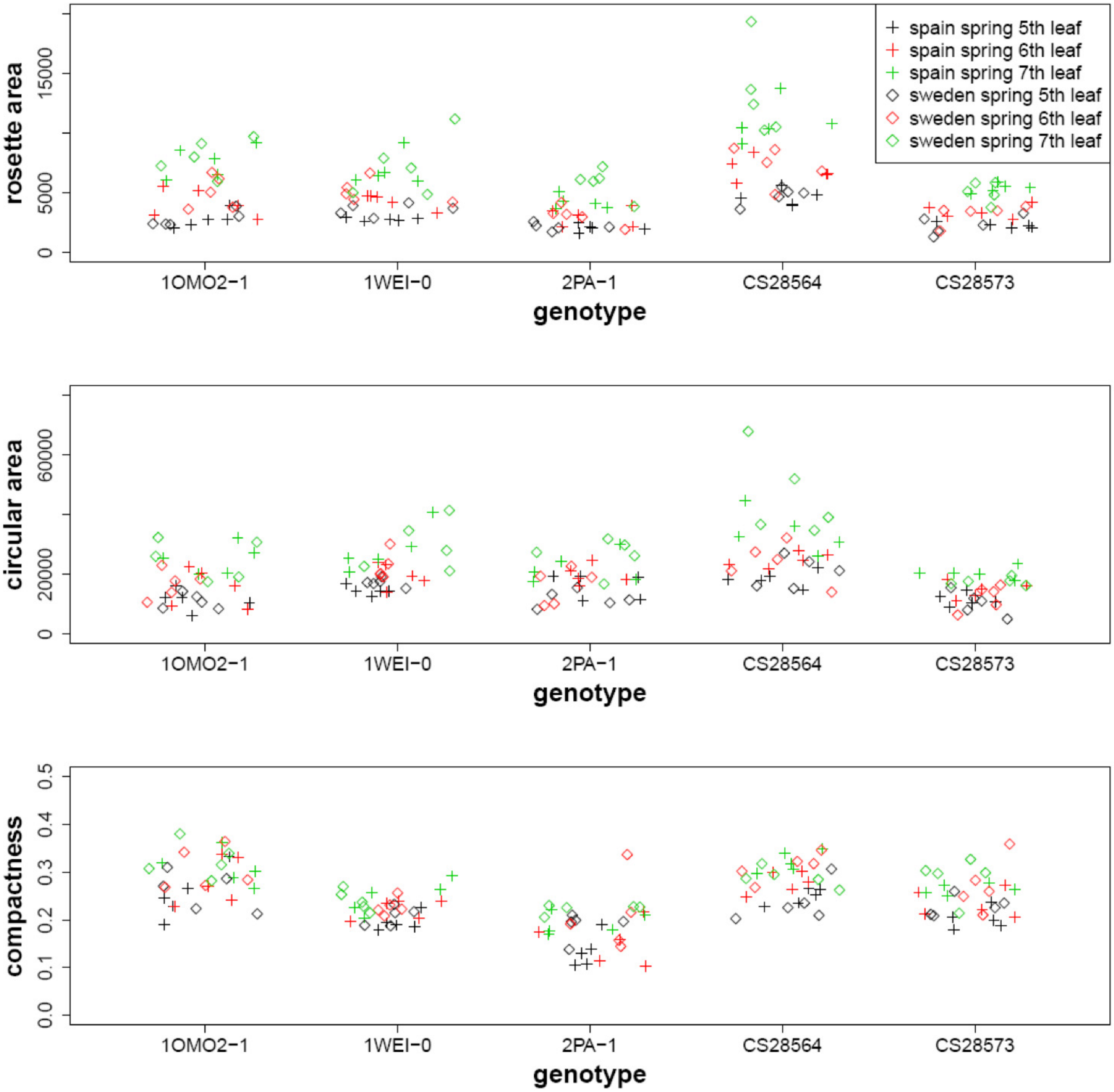

Figure S8 The rosette area (upper), circular area (middle) and compactness (lower) plotted for each genotype, across developmental stage 1.05 (black), 1.06 (red) and 1.07 (green), under Spain spring (cross points) and Sweden spring (diamond points) conditions.

Files S1-S3

Supporting Material

Files S1-S3 are available for download at http://www.g3journal.org/lookup/suppl/doi:10.1534/g3.111.001487/-/DC1.
