# Supplementary material for: Natural Genetic Variation for Growth and Development Revealed by High-Throughput Phenotyping in Arabidopsis thaliana
Source: G3 (Bethesda). 2012 Jan 1;2(1):29–34. doi: 10.1534/g3.111.001487 (PMC3276187; doi:10.1534/g3.111.001487)
Supplement: Supporting Information [file supp_2.1.29_FigureS1.pdf]

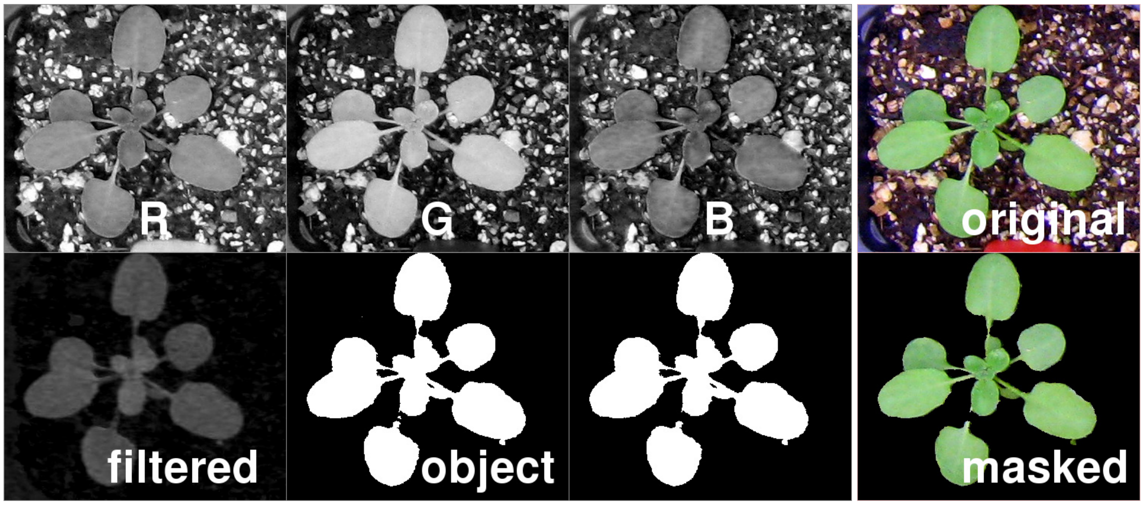

Figure S1 Detection of rosette. From left to right of the upper panel were red, green and blue channel, and the original image. From left to right of the lower panel were color-filtered image, image containing detected objects, image with noise objects removed, and the original image with background and noise objects removed.
