# Supplementary material for: Natural Genetic Variation for Growth and Development Revealed by High-Throughput Phenotyping in Arabidopsis thaliana
Source: G3 (Bethesda). 2012 Jan 1;2(1):29–34. doi: 10.1534/g3.111.001487 (PMC3276187; doi:10.1534/g3.111.001487)
Supplement: Supporting Information [file supp_2.1.29_FigureS2.pdf]

A

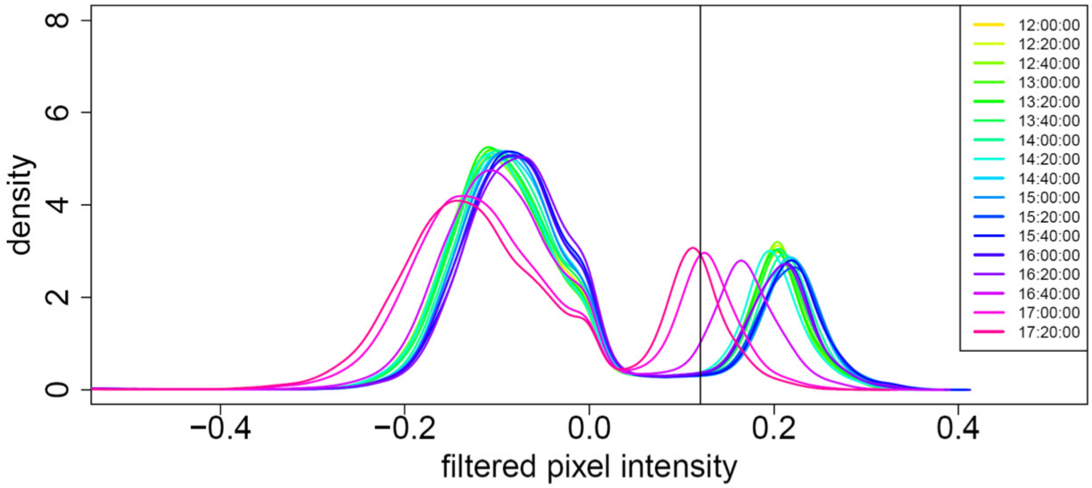

B

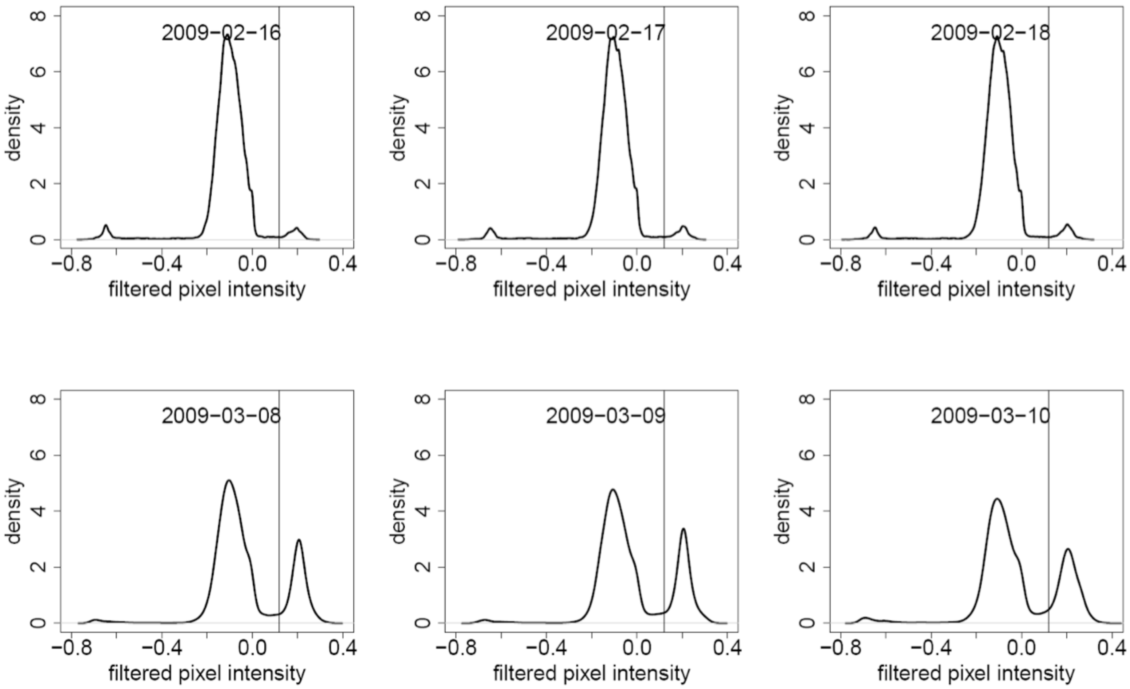

Figure S2 **(A)** The density distribution of pixel intensity for a cropped image, from noon to dusk within a day. The color filter applied here was green channel intensity – red channel intensity for simplification, thus the right-side peaks represent rosette pixels. A threshold of 0.12 (vertical line) is appropriate for noon but too high approaching sunset. **(B)** The density distribution of pixel intensity for a cropped image, at noon time across days. The color filter applied here was green channel intensity – red channel intensity for simplification, thus the right-side peaks represent rosette pixels. A threshold of 0.12 (vertical line) is appropriate when the rosette is small but too high as rosette grows.
