# Supplementary material for: Natural Genetic Variation for Growth and Development Revealed by High-Throughput Phenotyping in Arabidopsis thaliana
Source: G3 (Bethesda). 2012 Jan 1;2(1):29–34. doi: 10.1534/g3.111.001487 (PMC3276187; doi:10.1534/g3.111.001487)
Supplement: Supporting Information [file supp_2.1.29_FigureS3.pdf]

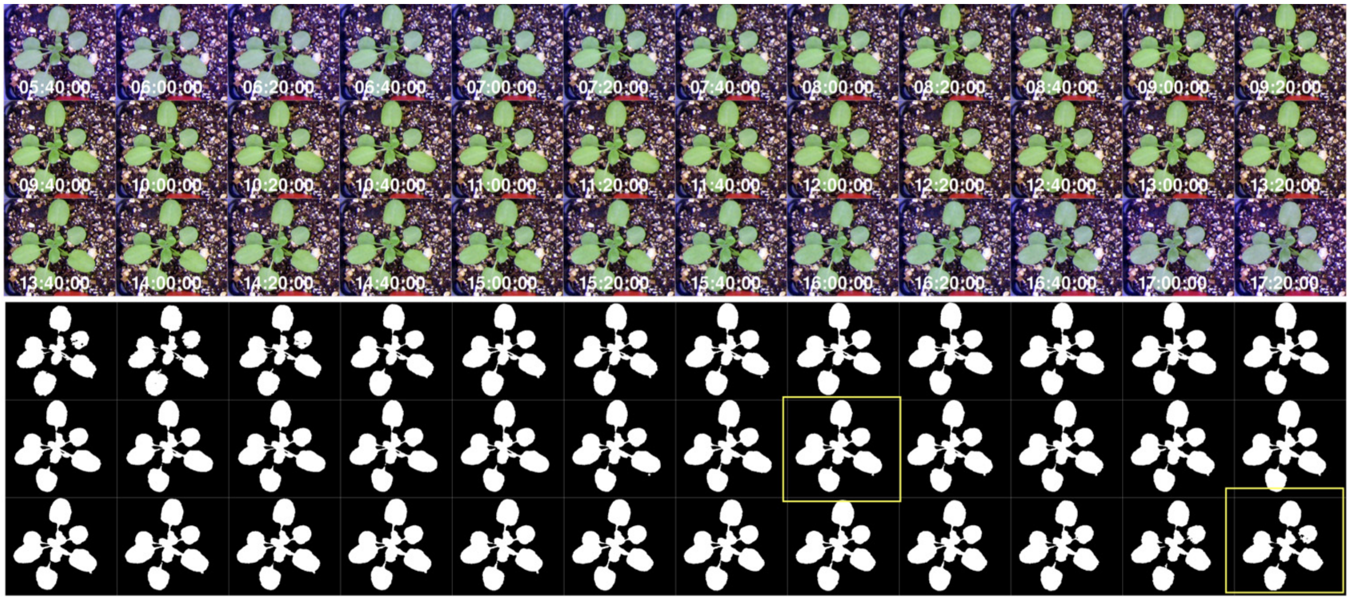

Figure S3 The detection of rosette across different time points from sunrise to sunset within a day. Note that rosette at noontime (12:00:00) is relatively extended than that toward dusk (17:20:00).
