# Supplementary material for: Natural Genetic Variation for Growth and Development Revealed by High-Throughput Phenotyping in Arabidopsis thaliana
Source: G3 (Bethesda). 2012 Jan 1;2(1):29–34. doi: 10.1534/g3.111.001487 (PMC3276187; doi:10.1534/g3.111.001487)
Supplement: Supporting Information [file supp_2.1.29_FigureS4.pdf]

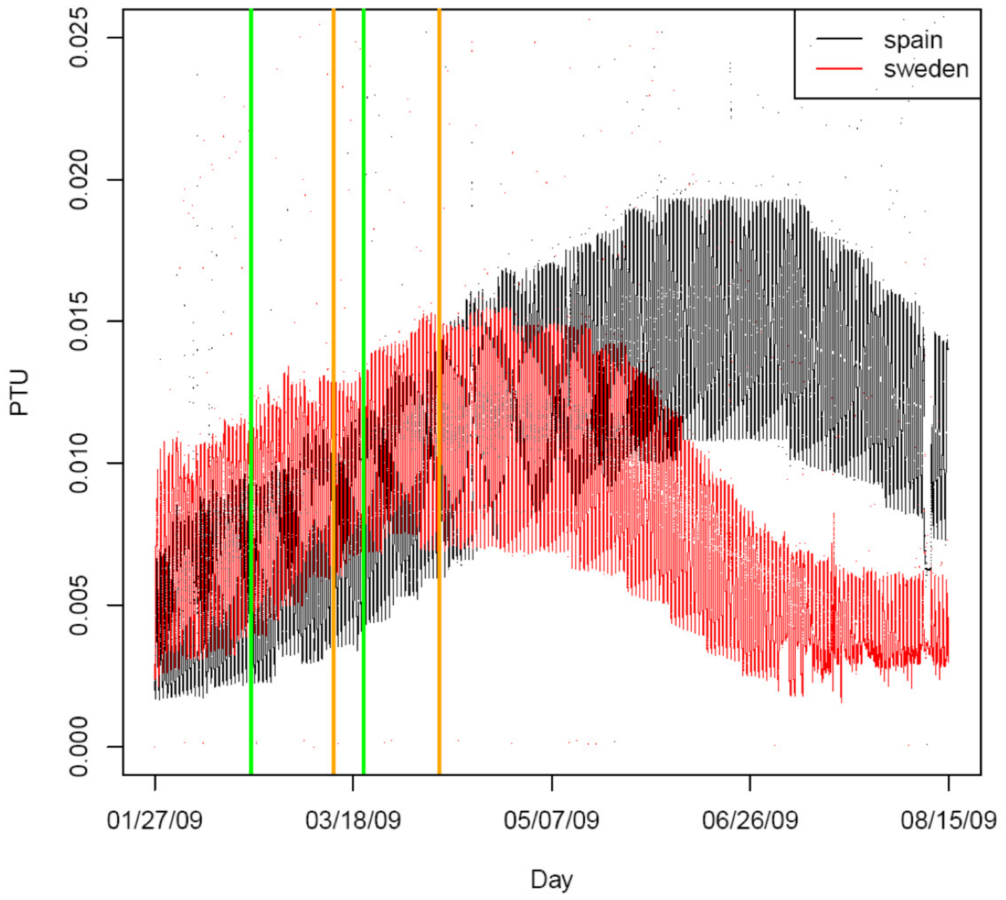

Figure S4 The differential of photothermal unit (ΔPTU) against time for simulated Spain (black) and Sweden (red) conditions. The interval between green lines represent the time interval analyzed for spring conditions, that between orange lines represent the time interval analyzed for summer conditions. ΔPTU was calculated according to Wilczek et al. 2009, for accession Col-0.

Reference: Wilczek AM, Roe JL, Knapp MC, Cooper MD, Lopez-Gallego C, Martin LJ, Muir CD, Sim S, Walker A, Anderson J, Egan JF, Moyers BT, Petipas R, Giakountis A, Charbit E, Coupland G, Welch SM and Schmitt J. 2009. Effects of genetic perturbation on seasonal life history plasticity. Science 323 (5916): 930-934.
