# Supplementary material for: Natural Genetic Variation for Growth and Development Revealed by High-Throughput Phenotyping in Arabidopsis thaliana
Source: G3 (Bethesda). 2012 Jan 1;2(1):29–34. doi: 10.1534/g3.111.001487 (PMC3276187; doi:10.1534/g3.111.001487)
Supplement: Supporting Information [file supp_2.1.29_FigureS5.pdf]

A

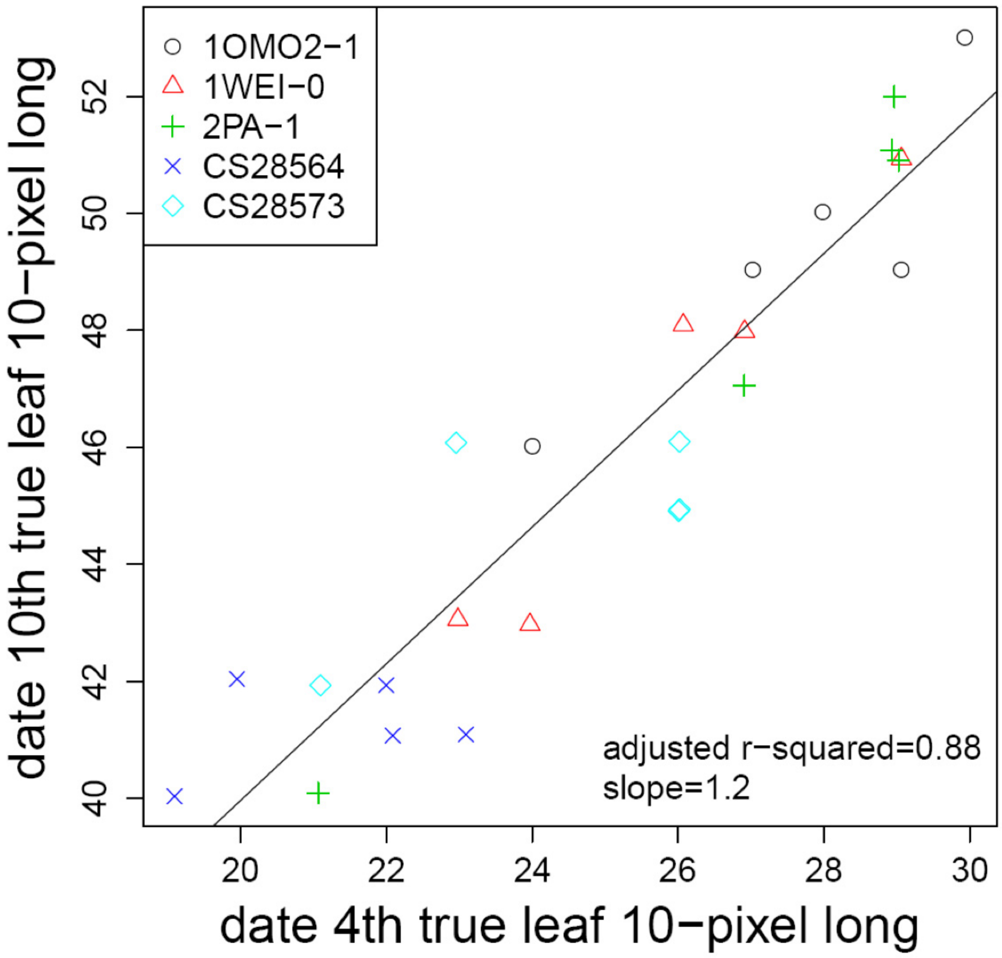
1OMO2−1
1WEI−0
2PA−1
CS28564
CS28573
adjusted r−squared=0.88
slope=1.2
date 10th true leaf 10−pixel long
40
42
44
46
48
50
52
20
22
24
26
28
30
date 4th true leaf 10−pixel long

B

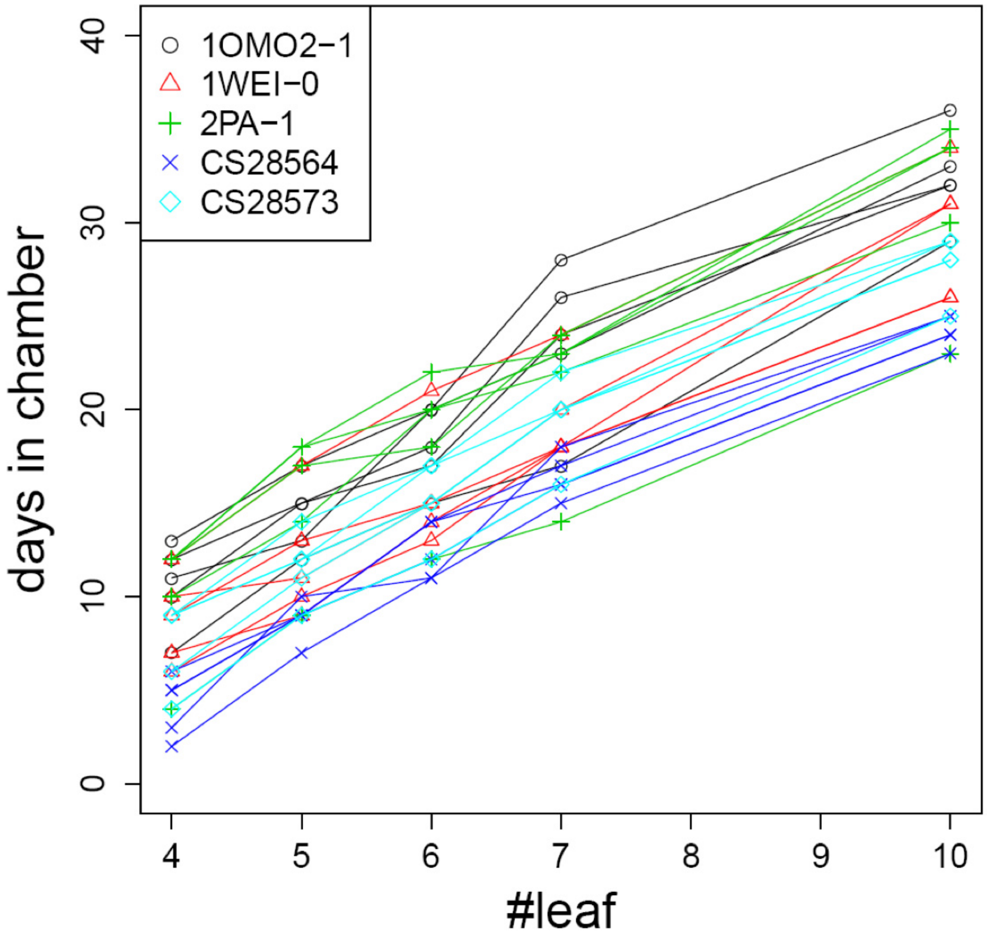

Figure S5 (A) . T1.10 is largely explained by T1.04. T1.10 was plotted against T1.04 for each plant. Colors denoted different accessions. The data points were jittered slightly on both x-axis and y-axis to avoid overlap. The adjusted r^2 is 0.88 for the model: T1.10 ~ T1.04 + ε.

(B) Leaf initiation was relatively synchronized once T1.04 was controlled. The number of days plant grown in chamber was plotted against the rosette developmental stage (when a specific leaf is 10-pixel in length). Data were analyzed by a linear model:

Ti (i > 1.04) ~ T1.04 + development + genotype + development x genotype + ε

Where development is a four-level factor including the 1.05, 1.06, 1.07 and 1.10 stages. Variance partitioning of T.1.04 (20%), development (76%), genotype (0.3%), genotype x development (0.6%).
