# Supplementary material for: Natural Genetic Variation for Growth and Development Revealed by High-Throughput Phenotyping in Arabidopsis thaliana
Source: G3 (Bethesda). 2012 Jan 1;2(1):29–34. doi: 10.1534/g3.111.001487 (PMC3276187; doi:10.1534/g3.111.001487)
Supplement: Supporting Information [file supp_2.1.29_FigureS6.pdf]

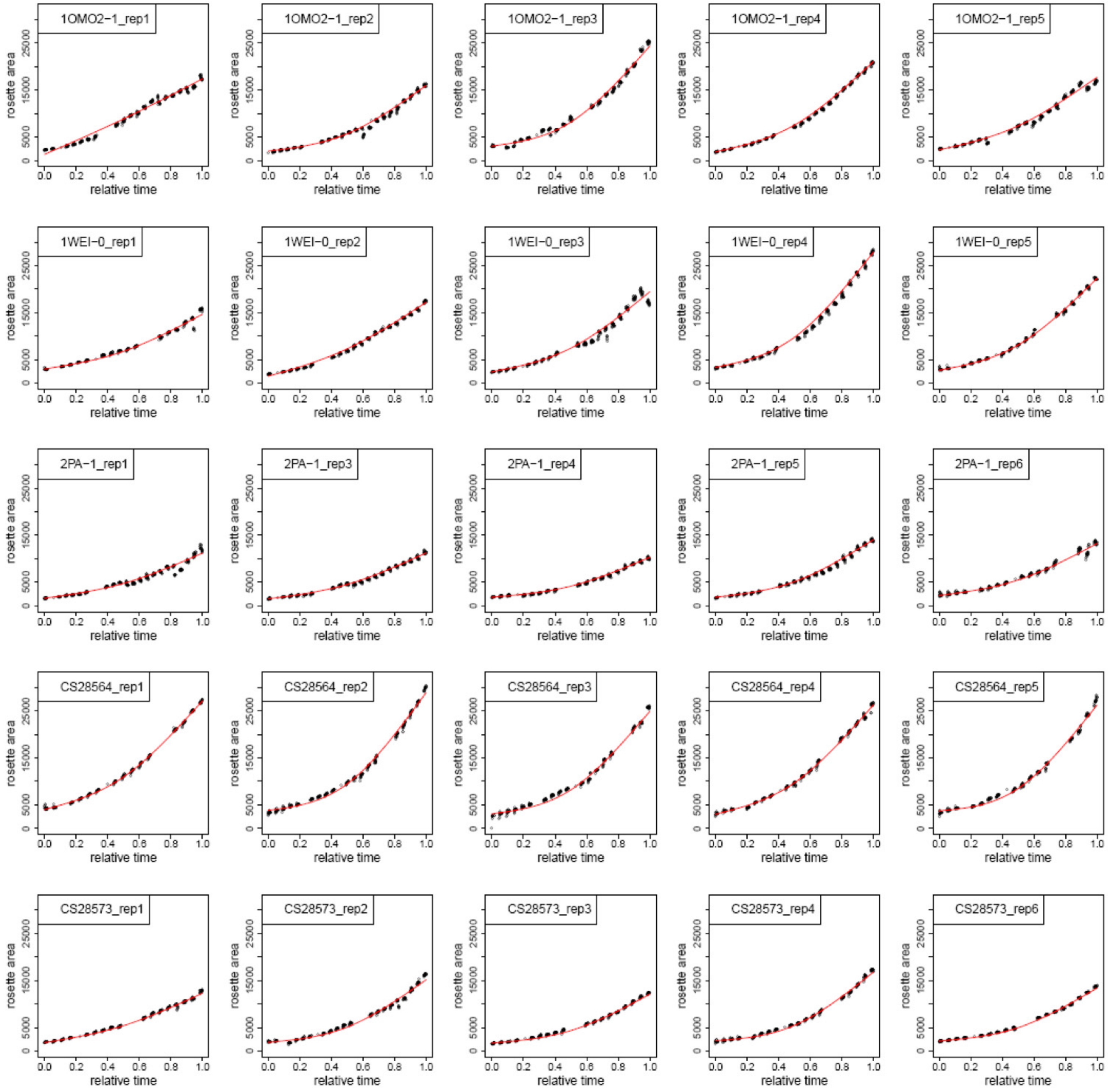

Figure S6 Fit rosette area (RA) against time by a one-knot spline for 5 accessions x 5 replicates grown in Spain spring condition. Cropped images taken from 8:00AM of the day at stage 1.04 to 4:00PM of the day at stage 1.10 were analyzed.
