# Supplementary material for: Natural Genetic Variation for Growth and Development Revealed by High-Throughput Phenotyping in Arabidopsis thaliana
Source: G3 (Bethesda). 2012 Jan 1;2(1):29–34. doi: 10.1534/g3.111.001487 (PMC3276187; doi:10.1534/g3.111.001487)
Supplement: Supporting Information [file supp_2.1.29_FigureS7.pdf]

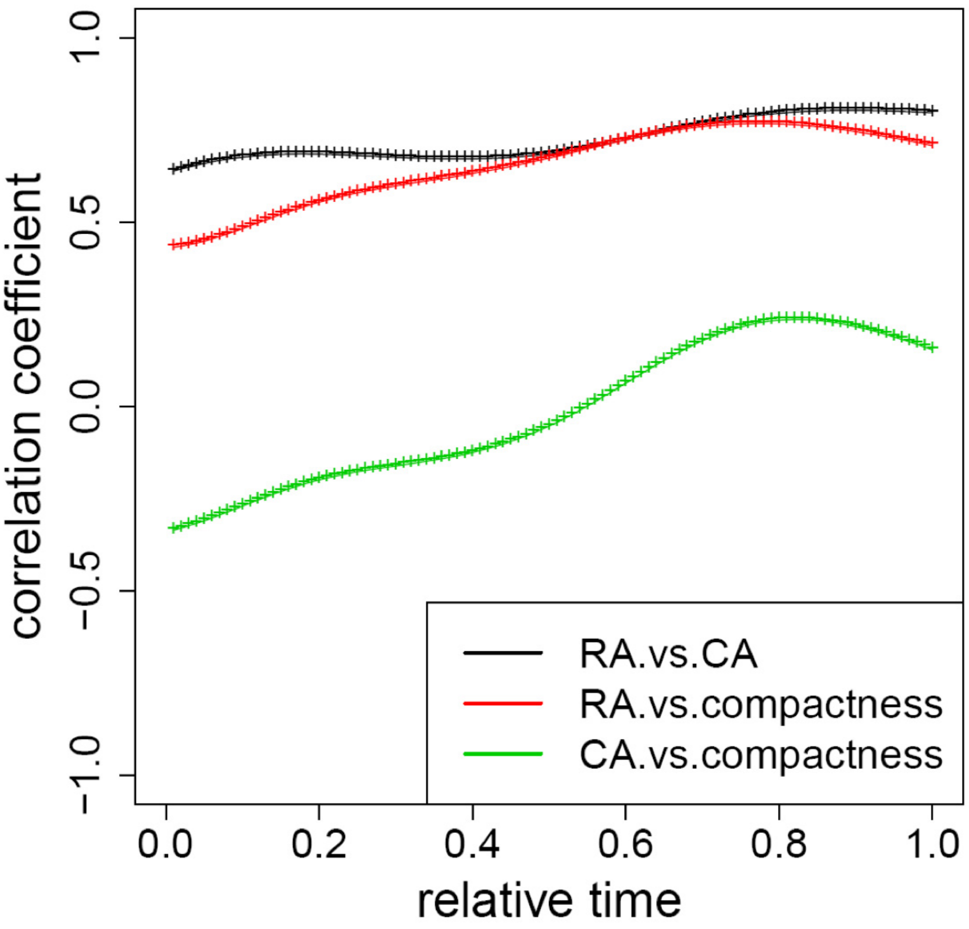

Figure S7 Correlation between traits. The correlation coefficients were plotted against relative developmental time for RA verse CA (black), RA verse compactness (red), and CA verse compactness (green). The correlation was calculated on spline-fitted data points.
