# Supplementary material for: Natural Genetic Variation for Growth and Development Revealed by High-Throughput Phenotyping in Arabidopsis thaliana
Source: G3 (Bethesda). 2012 Jan 1;2(1):29–34. doi: 10.1534/g3.111.001487 (PMC3276187; doi:10.1534/g3.111.001487)
Supplement: Supporting Information [file supp_2.1.29_FigureS8.pdf]

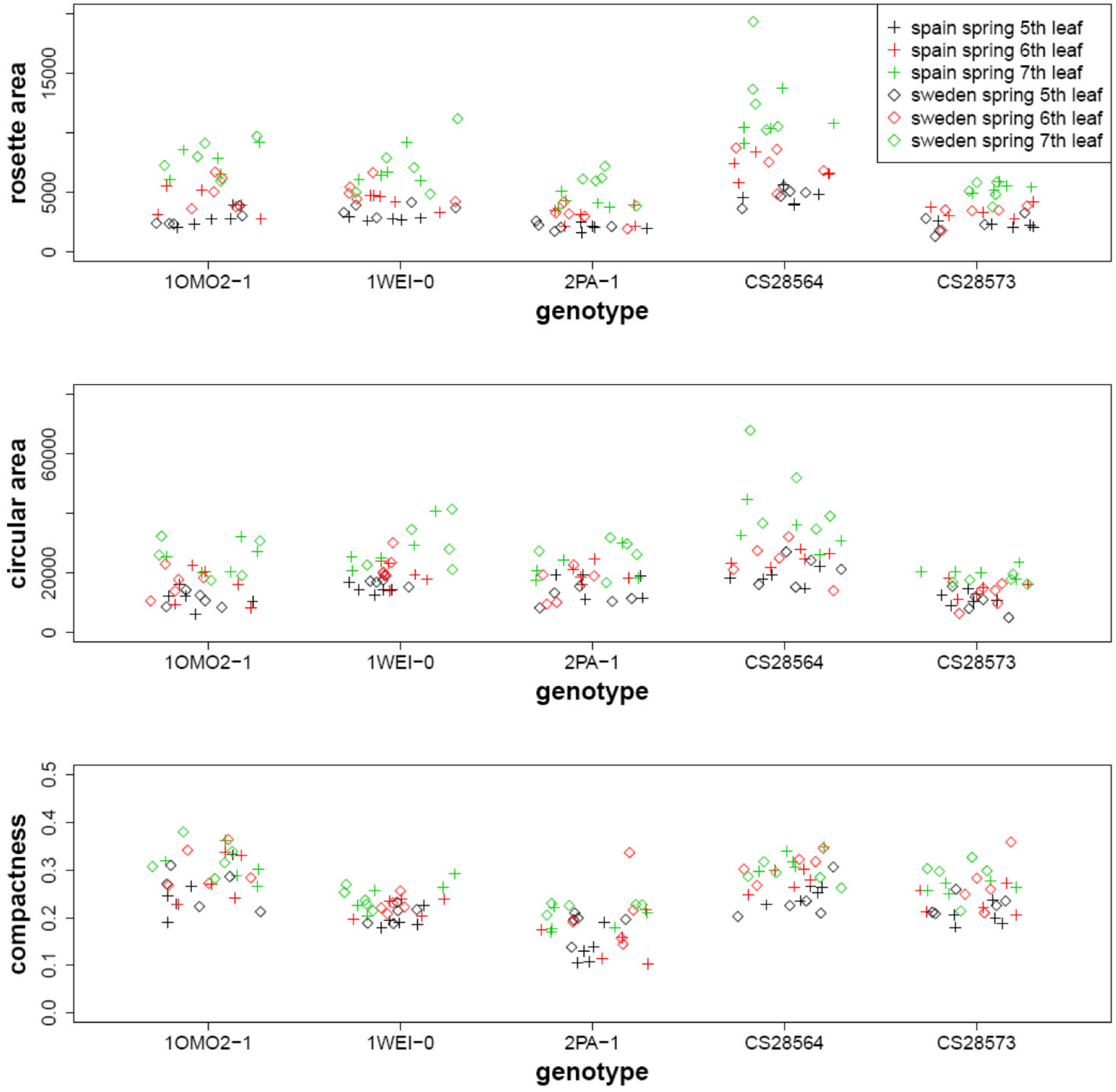

Figure S8 The rosette area (upper), circular area (middle) and compactness (lower) plotted for each genotype, across developmental stage 1.05 (black), 1.06 (red) and 1.07 (green), under Spain spring (cross points) and Sweden spring (diamond points) conditions.
