# Supplementary material for: Natural Genetic Variation for Growth and Development Revealed by High-Throughput Phenotyping in Arabidopsis thaliana
Source: G3 (Bethesda). 2012 Jan 1;2(1):29–34. doi: 10.1534/g3.111.001487 (PMC3276187; doi:10.1534/g3.111.001487)
Supplement: Supporting Information [file supp_2.1.29_FileS2.pdf]

Rosette Area Data Summary

Each page in the .pdf file present 6x6 plants from one flat. Some plants grown along the border of a flat were out of focus and were not included here. In each of the four environments plants were grown in four flats, two placed on top shelf and two placed on bottom shelf. (1) Plants in the two flats within Spain spring bottom shelf grew quite differently from those within top shelf. Thus only two flats within top shelf in Spain spring were analyzed in GWAS. (2) Plants in the two flats within Spain summer top shelf had a significant amount of missing images. Thus only two flats within bottom shelf in Spain summer were analyzed in GWAS. (3) A proportion of plants flowered very early in Sweden spring, leaving short developmental frame to be analyzed. Particularly plants grown in the two flats within Sweden spring bottom shelf were thinned very late. For Sweden summer, green labels were applied for many flats which made the estimation of rosette area unreliable. Thus the Sweden data were not analyzed in GWAS.

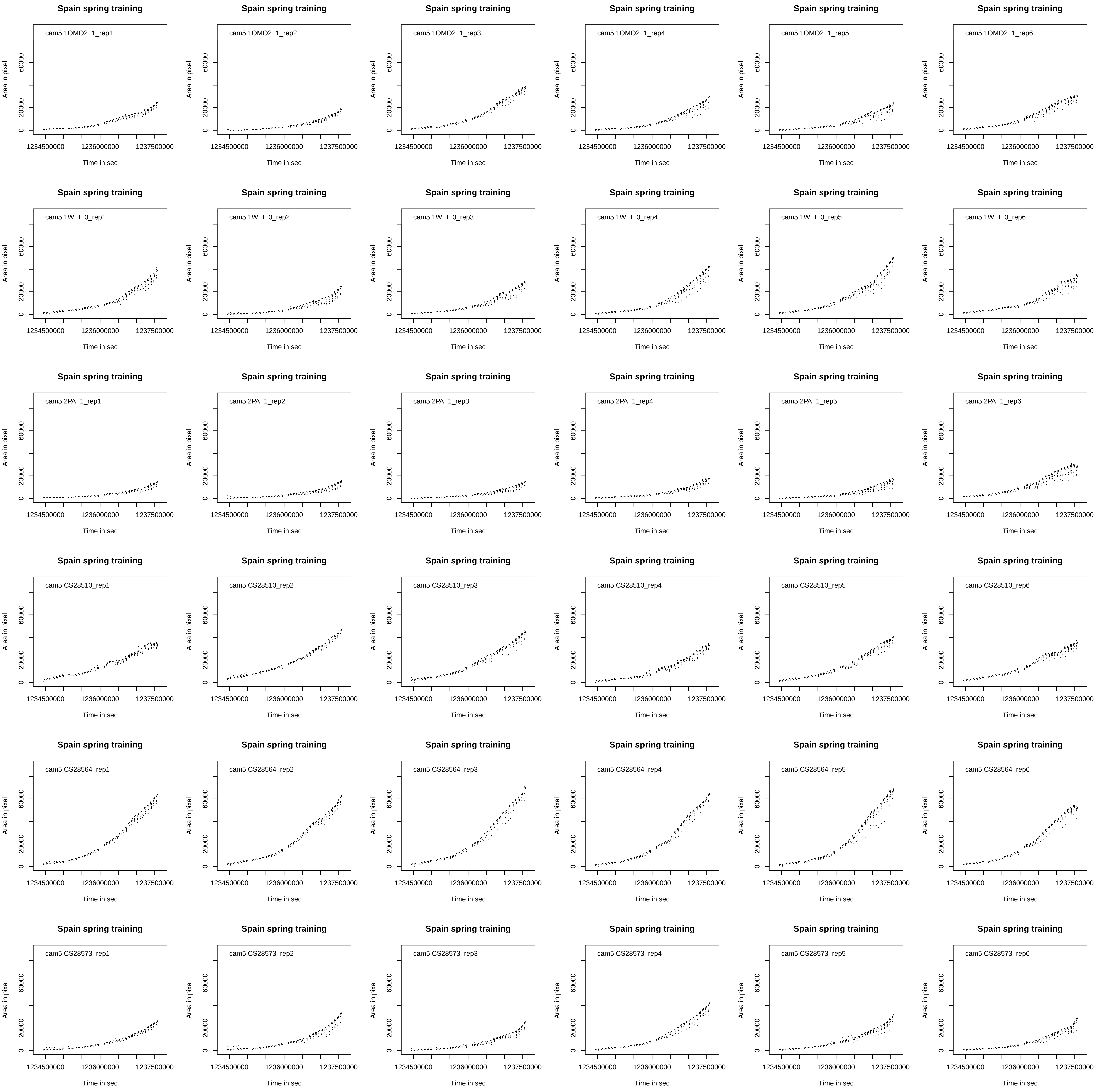
Spain spring training
cam5 1OMO2−1_rep1
cam5 1OMO2−1_rep2
cam5 1OMO2−1_rep3
cam5 1OMO2−1_rep4
cam5 1OMO2−1_rep5
cam5 1OMO2−1_rep6
cam5 1WEI−0_rep1
cam5 1WEI−0_rep2
cam5 1WEI−0_rep3
cam5 1WEI−0_rep4
cam5 1WEI−0_rep5
cam5 1WEI−0_rep6
cam5 2PA−1_rep1
cam5 2PA−1_rep2
cam5 2PA−1_rep3
cam5 2PA−1_rep4
cam5 2PA−1_rep5
cam5 2PA−1_rep6
cam5 CS28510_rep1
cam5 CS28510_rep2
cam5 CS28510_rep3
cam5 CS28510_rep4
cam5 CS28510_rep5
cam5 CS28510_rep6
cam5 CS28564_rep1
cam5 CS28564_rep2
cam5 CS28564_rep3
cam5 CS28564_rep4
cam5 CS28564_rep5
cam5 CS28564_rep6
cam5 CS28573_rep1
cam5 CS28573_rep2
cam5 CS28573_rep3
cam5 CS28573_rep4
cam5 CS28573_rep5
cam5 CS28573_rep6
Area in pixel
0
20000
60000
Time in sec
1234500000
1236000000
1237500000

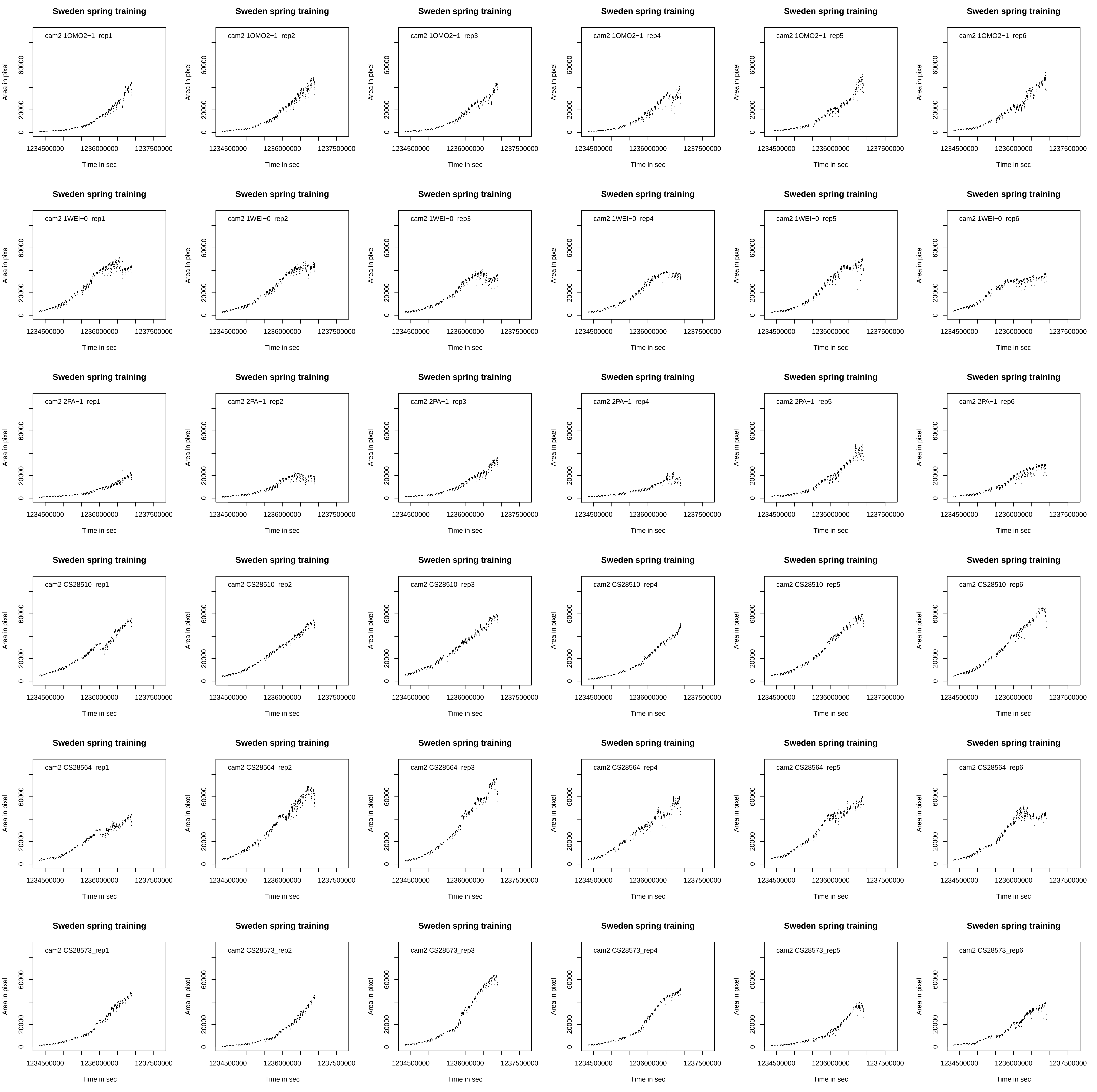
Sweden spring training
cam2 1OMO2−1_rep1
cam2 1OMO2−1_rep2
cam2 1OMO2−1_rep3
cam2 1OMO2−1_rep4
cam2 1OMO2−1_rep5
cam2 1OMO2−1_rep6
cam2 1WEI−0_rep1
cam2 1WEI−0_rep2
cam2 1WEI−0_rep3
cam2 1WEI−0_rep4
cam2 1WEI−0_rep5
cam2 1WEI−0_rep6
cam2 2PA−1_rep1
cam2 2PA−1_rep2
cam2 2PA−1_rep3
cam2 2PA−1_rep4
cam2 2PA−1_rep5
cam2 2PA−1_rep6
cam2 CS28510_rep1
cam2 CS28510_rep2
cam2 CS28510_rep3
cam2 CS28510_rep4
cam2 CS28510_rep5
cam2 CS28510_rep6
cam2 CS28564_rep1
cam2 CS28564_rep2
cam2 CS28564_rep3
cam2 CS28564_rep4
cam2 CS28564_rep5
cam2 CS28564_rep6
cam2 CS28573_rep1
cam2 CS28573_rep2
cam2 CS28573_rep3
cam2 CS28573_rep4
cam2 CS28573_rep5
cam2 CS28573_rep6
Area in pixel
0
20000
60000
Time in sec
1234500000
1236000000
1237500000

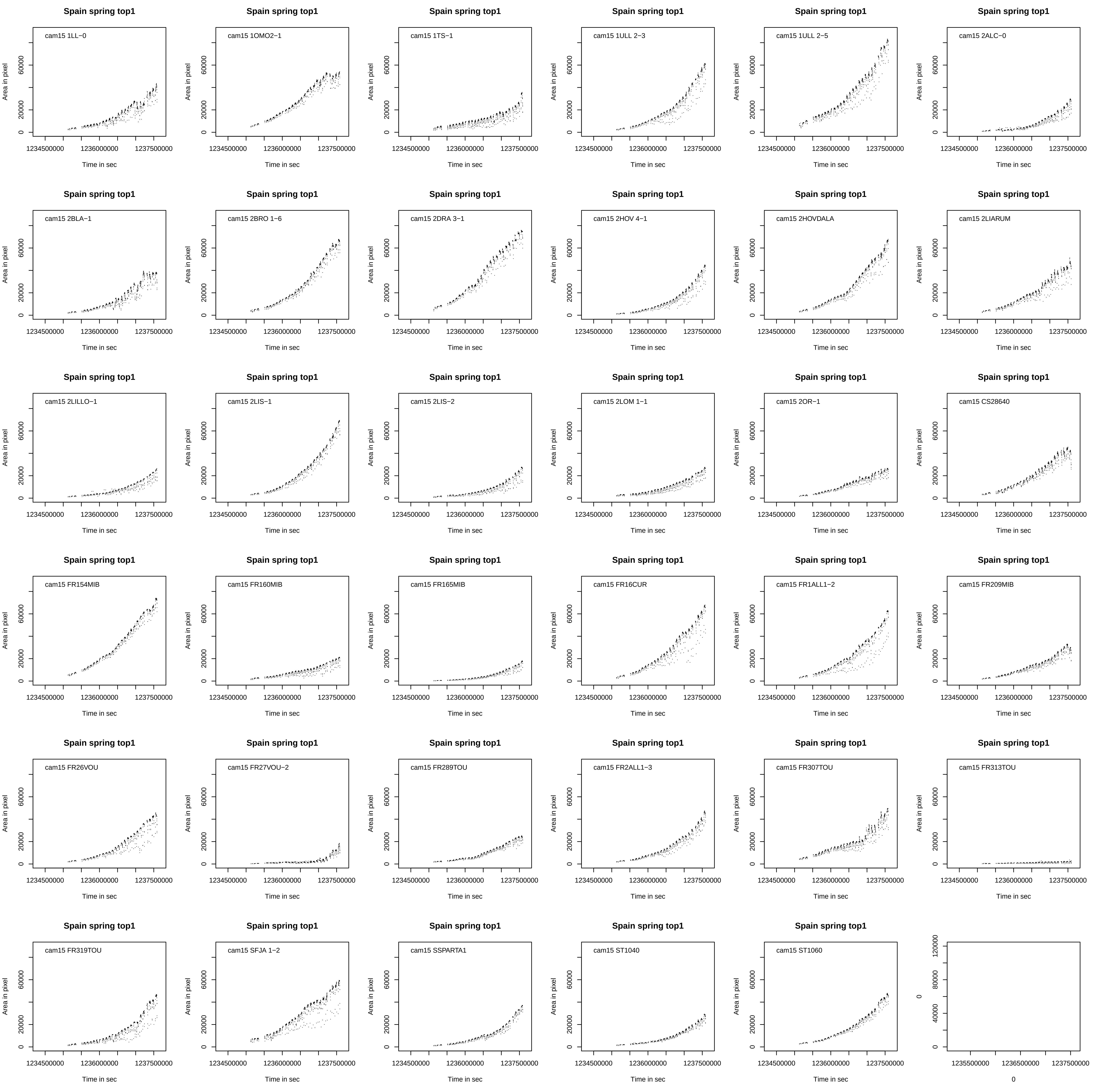
Spain spring top1
cam15 1LL−0
Spain spring top1
cam15 1OMO2−1
Spain spring top1
cam15 1TS−1
Spain spring top1
cam15 1ULL 2−3
Spain spring top1
cam15 1ULL 2−5
Spain spring top1
cam15 2ALC−0
Spain spring top1
cam15 2BLA−1
Spain spring top1
cam15 2BRO 1−6
Spain spring top1
cam15 2DRA 3−1
Spain spring top1
cam15 2HOV 4−1
Spain spring top1
cam15 2HOVDALA
Spain spring top1
cam15 2LIARUM
Spain spring top1
cam15 2LILLO−1
Spain spring top1
cam15 2LIS−1
Spain spring top1
cam15 2LIS−2
Spain spring top1
cam15 2LOM 1−1
Spain spring top1
cam15 2OR−1
Spain spring top1
cam15 CS28640
Spain spring top1
cam15 FR154MIB
Spain spring top1
cam15 FR160MIB
Spain spring top1
cam15 FR165MIB
Spain spring top1
cam15 FR16CUR
Spain spring top1
cam15 FR1ALL1−2
Spain spring top1
cam15 FR209MIB
Spain spring top1
cam15 FR26VOU
Spain spring top1
cam15 FR27VOU−2
Spain spring top1
cam15 FR289TOU
Spain spring top1
cam15 FR2ALL1−3
Spain spring top1
cam15 FR307TOU
Spain spring top1
cam15 FR313TOU
Spain spring top1
cam15 FR319TOU
Spain spring top1
cam15 SFJA 1−2
Spain spring top1
cam15 SSPARTA1
Spain spring top1
cam15 ST1040
Spain spring top1
cam15 ST1060
Area in pixel
0
20000
60000
Time in sec
1234500000
1236000000
1237500000
0
40000
80000
120000
1235500000
1236500000
1237500000

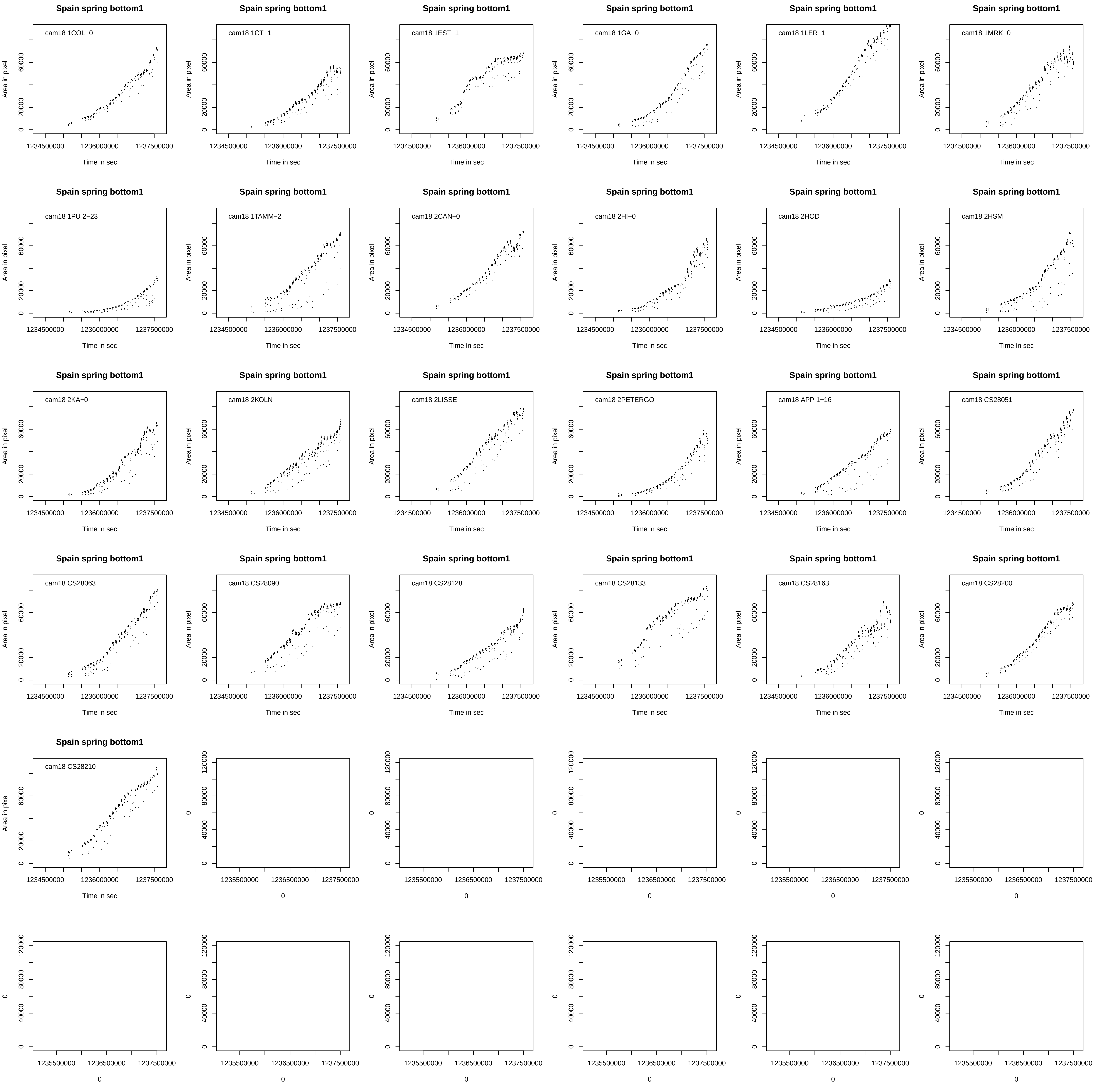
Spain spring bottom1
cam18 1COL−0
cam18 1CT−1
cam18 1EST−1
cam18 1GA−0
cam18 1LER−1
cam18 1MRK−0
cam18 1PU 2−23
cam18 1TAMM−2
cam18 2CAN−0
cam18 2HI−0
cam18 2HOD
cam18 2HSM
cam18 2KA−0
cam18 2KOLN
cam18 2LISSE
cam18 2PETERGO
cam18 APP 1−16
cam18 CS28051
cam18 CS28063
cam18 CS28090
cam18 CS28128
cam18 CS28133
cam18 CS28163
cam18 CS28200
cam18 CS28210
Area in pixel
0
20000
60000
Time in sec
1234500000
1236000000
1237500000
40000
80000
120000
1235500000
1236500000

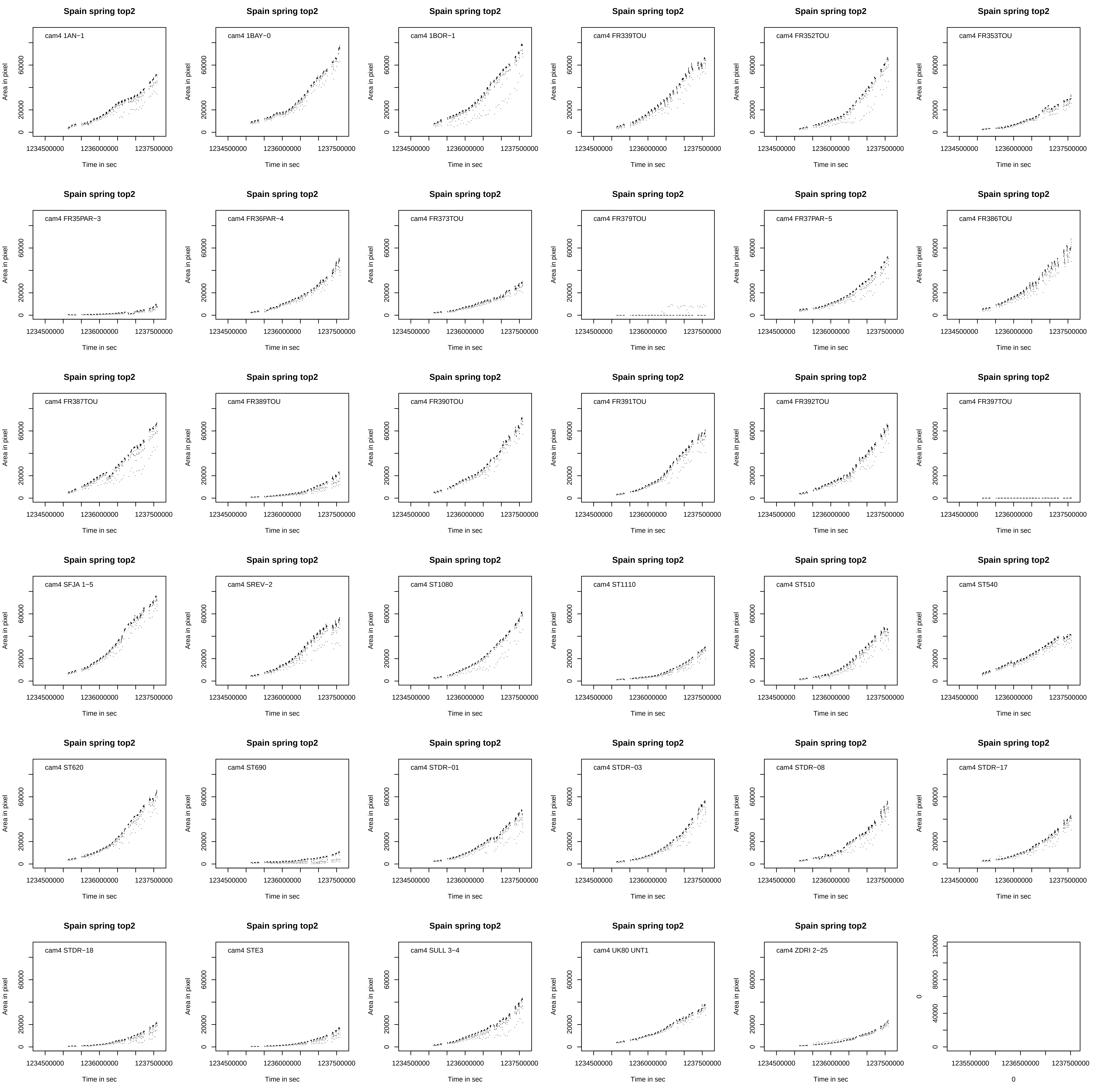
Spain spring top2
cam4 1AN−1
Spain spring top2
cam4 1BAY−0
Spain spring top2
cam4 1BOR−1
Spain spring top2
cam4 FR339TOU
Spain spring top2
cam4 FR352TOU
Spain spring top2
cam4 FR353TOU
Spain spring top2
cam4 FR35PAR−3
Spain spring top2
cam4 FR36PAR−4
Spain spring top2
cam4 FR373TOU
Spain spring top2
cam4 FR379TOU
Spain spring top2
cam4 FR37PAR−5
Spain spring top2
cam4 FR386TOU
Spain spring top2
cam4 FR387TOU
Spain spring top2
cam4 FR389TOU
Spain spring top2
cam4 FR390TOU
Spain spring top2
cam4 FR391TOU
Spain spring top2
cam4 FR392TOU
Spain spring top2
cam4 FR397TOU
Spain spring top2
cam4 SFJA 1−5
Spain spring top2
cam4 SREV−2
Spain spring top2
cam4 ST1080
Spain spring top2
cam4 ST1110
Spain spring top2
cam4 ST510
Spain spring top2
cam4 ST540
Spain spring top2
cam4 ST620
Spain spring top2
cam4 ST690
Spain spring top2
cam4 STDR−01
Spain spring top2
cam4 STDR−03
Spain spring top2
cam4 STDR−08
Spain spring top2
cam4 STDR−17
Spain spring top2
cam4 STDR−18
Spain spring top2
cam4 STE3
Spain spring top2
cam4 SULL 3−4
Spain spring top2
cam4 UK80 UNT1
Spain spring top2
cam4 ZDRI 2−25
Area in pixel
Time in sec
0
20000
60000
1234500000
1236000000
1237500000

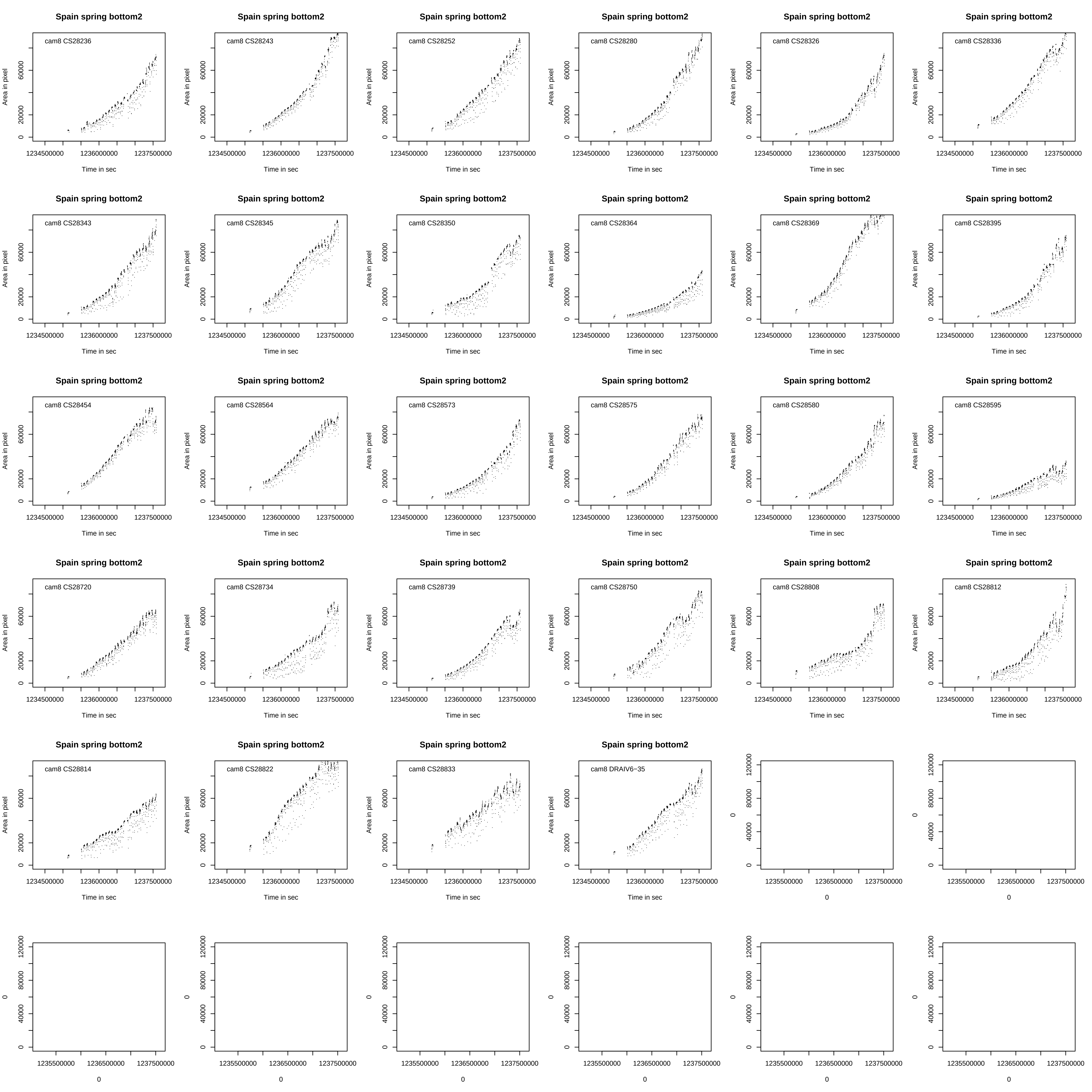
Spain spring bottom2
cam8 CS28236
Spain spring bottom2
cam8 CS28243
Spain spring bottom2
cam8 CS28252
Spain spring bottom2
cam8 CS28280
Spain spring bottom2
cam8 CS28326
Spain spring bottom2
cam8 CS28336
Spain spring bottom2
cam8 CS28343
Spain spring bottom2
cam8 CS28345
Spain spring bottom2
cam8 CS28350
Spain spring bottom2
cam8 CS28364
Spain spring bottom2
cam8 CS28369
Spain spring bottom2
cam8 CS28395
Spain spring bottom2
cam8 CS28454
Spain spring bottom2
cam8 CS28564
Spain spring bottom2
cam8 CS28573
Spain spring bottom2
cam8 CS28575
Spain spring bottom2
cam8 CS28580
Spain spring bottom2
cam8 CS28595
Spain spring bottom2
cam8 CS28720
Spain spring bottom2
cam8 CS28734
Spain spring bottom2
cam8 CS28739
Spain spring bottom2
cam8 CS28750
Spain spring bottom2
cam8 CS28808
Spain spring bottom2
cam8 CS28812
Spain spring bottom2
cam8 CS28814
Spain spring bottom2
cam8 CS28822
Spain spring bottom2
cam8 CS28833
Spain spring bottom2
cam8 DRAIV6−35
Area in pixel
Time in sec
0
20000
60000
1234500000
1236000000
1237500000
40000
80000
120000
1235500000
1236500000

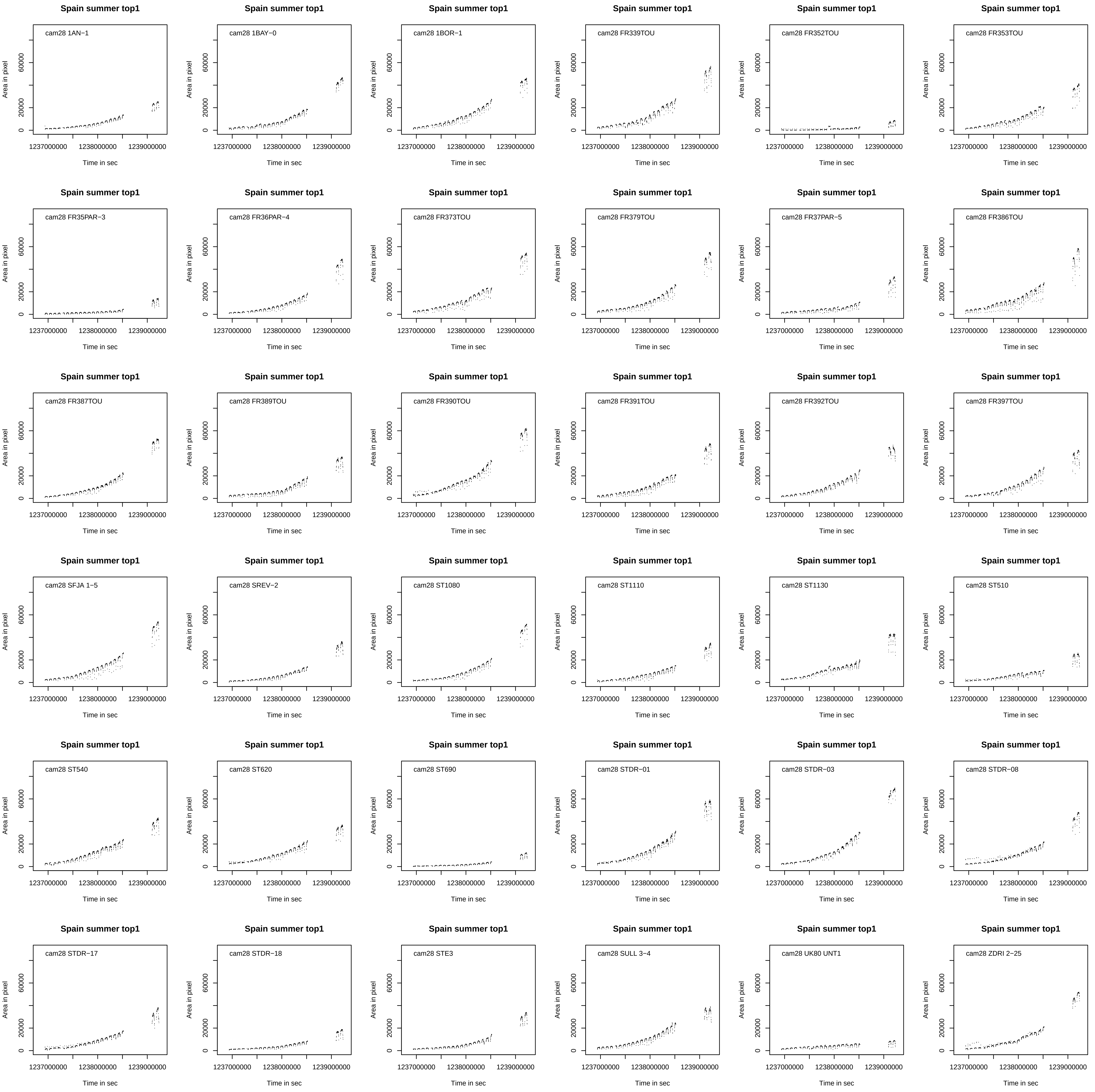
Spain summer top1
cam28 1AN−1
cam28 1BAY−0
cam28 1BOR−1
cam28 FR339TOU
cam28 FR352TOU
cam28 FR353TOU
cam28 FR35PAR−3
cam28 FR36PAR−4
cam28 FR373TOU
cam28 FR379TOU
cam28 FR37PAR−5
cam28 FR386TOU
cam28 FR387TOU
cam28 FR389TOU
cam28 FR390TOU
cam28 FR391TOU
cam28 FR392TOU
cam28 FR397TOU
cam28 SFJA 1−5
cam28 SREV−2
cam28 ST1080
cam28 ST1110
cam28 ST1130
cam28 ST510
cam28 ST540
cam28 ST620
cam28 ST690
cam28 STDR−01
cam28 STDR−03
cam28 STDR−08
cam28 STDR−17
cam28 STDR−18
cam28 STE3
cam28 SULL 3−4
cam28 UK80 UNT1
cam28 ZDRI 2−25
Area in pixel
0
20000
60000
Time in sec
1237000000
1238000000
1239000000

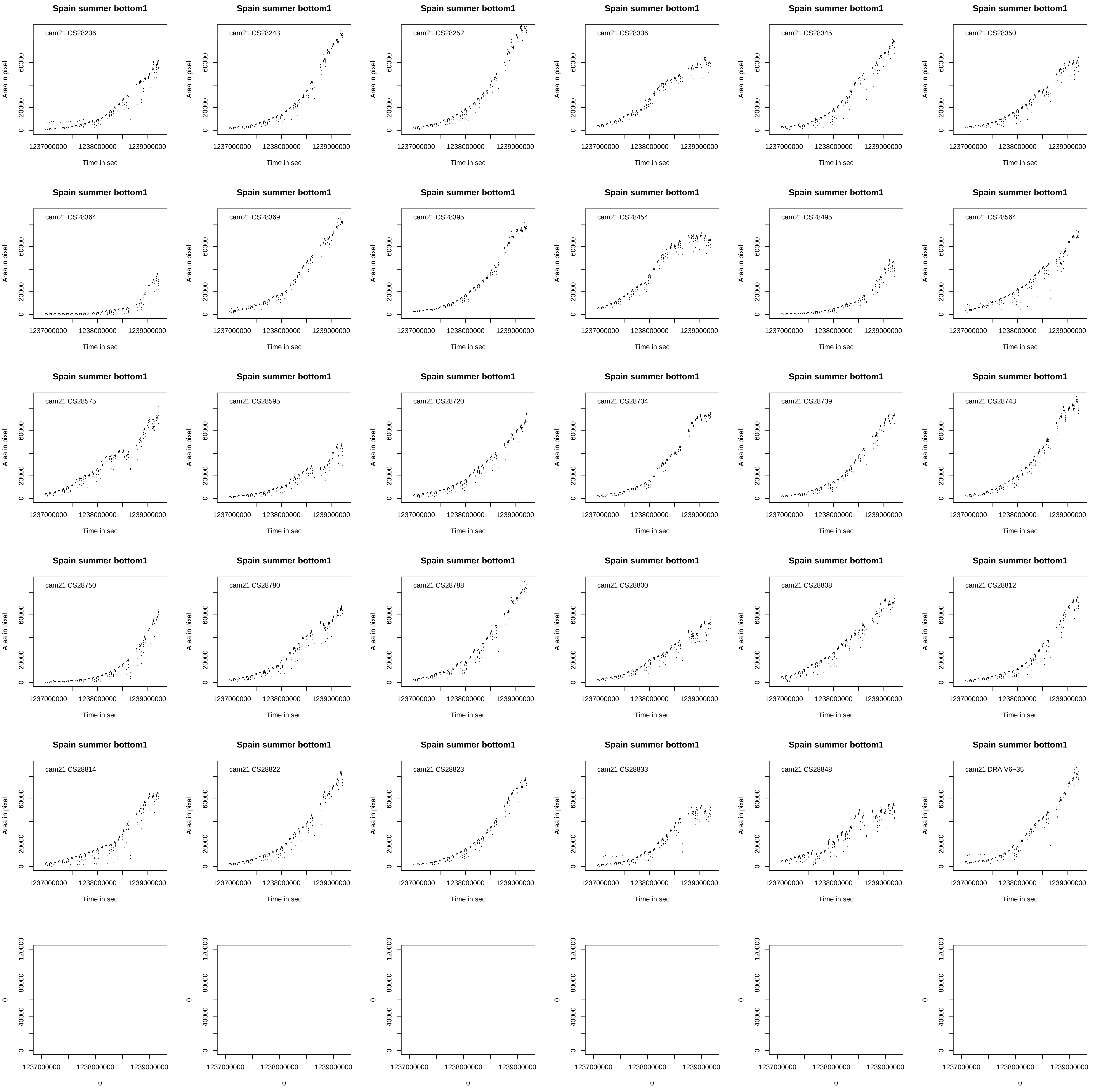
Spain summer bottom1
cam21 CS28236
Spain summer bottom1
cam21 CS28243
Spain summer bottom1
cam21 CS28252
Spain summer bottom1
cam21 CS28336
Spain summer bottom1
cam21 CS28345
Spain summer bottom1
cam21 CS28350
Spain summer bottom1
cam21 CS28364
Spain summer bottom1
cam21 CS28369
Spain summer bottom1
cam21 CS28395
Spain summer bottom1
cam21 CS28454
Spain summer bottom1
cam21 CS28495
Spain summer bottom1
cam21 CS28564
Spain summer bottom1
cam21 CS28575
Spain summer bottom1
cam21 CS28595
Spain summer bottom1
cam21 CS28720
Spain summer bottom1
cam21 CS28734
Spain summer bottom1
cam21 CS28739
Spain summer bottom1
cam21 CS28743
Spain summer bottom1
cam21 CS28750
Spain summer bottom1
cam21 CS28780
Spain summer bottom1
cam21 CS28788
Spain summer bottom1
cam21 CS28800
Spain summer bottom1
cam21 CS28808
Spain summer bottom1
cam21 CS28812
Spain summer bottom1
cam21 CS28814
Spain summer bottom1
cam21 CS28822
Spain summer bottom1
cam21 CS28823
Spain summer bottom1
cam21 CS28833
Spain summer bottom1
cam21 CS28848
Spain summer bottom1
cam21 DRAIV6−35
Area in pixel
Time in sec

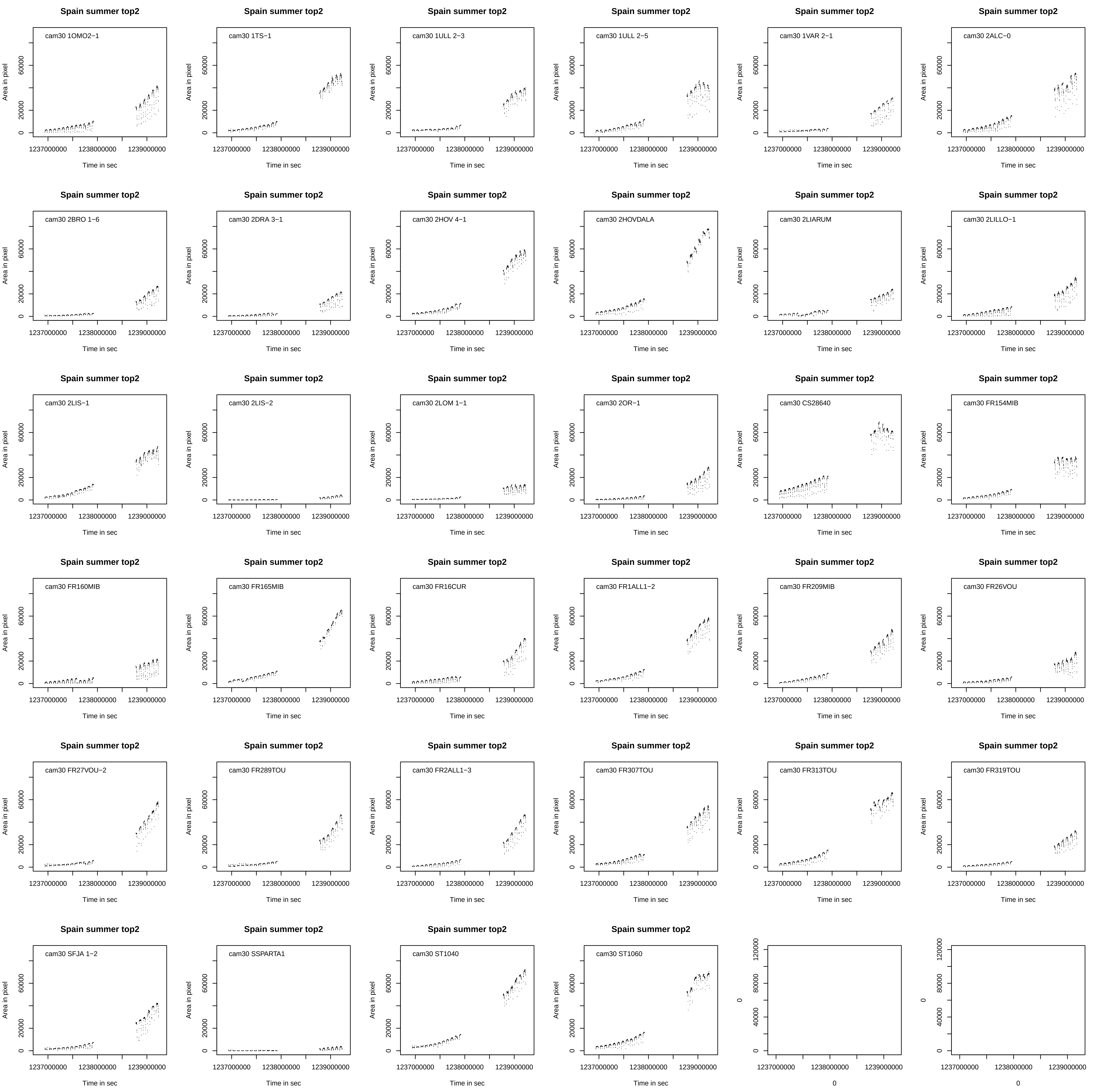

Spain summer top2
cam30 1OMO2−1
Spain summer top2
cam30 1TS−1
Spain summer top2
cam30 1ULL 2−3
Spain summer top2
cam30 1ULL 2−5
Spain summer top2
cam30 1VAR 2−1
Spain summer top2
cam30 2ALC−0
Spain summer top2
cam30 2BRO 1−6
Spain summer top2
cam30 2DRA 3−1
Spain summer top2
cam30 2HOV 4−1
Spain summer top2
cam30 2HOVDALA
Spain summer top2
cam30 2LIARUM
Spain summer top2
cam30 2LILLO−1
Spain summer top2
cam30 2LIS−1
Spain summer top2
cam30 2LIS−2
Spain summer top2
cam30 2LOM 1−1
Spain summer top2
cam30 2OR−1
Spain summer top2
cam30 CS28640
Spain summer top2
cam30 FR154MIB
Spain summer top2
cam30 FR160MIB
Spain summer top2
cam30 FR165MIB
Spain summer top2
cam30 FR16CUR
Spain summer top2
cam30 FR1ALL1−2
Spain summer top2
cam30 FR209MIB
Spain summer top2
cam30 FR26VOU
Spain summer top2
cam30 FR27VOU−2
Spain summer top2
cam30 FR289TOU
Spain summer top2
cam30 FR2ALL1−3
Spain summer top2
cam30 FR307TOU
Spain summer top2
cam30 FR313TOU
Spain summer top2
cam30 FR319TOU
Spain summer top2
cam30 SFJA 1−2
Spain summer top2
cam30 SSPARTA1
Spain summer top2
cam30 ST1040
Spain summer top2
cam30 ST1060
Area in pixel
0
20000
60000
Time in sec
1237000000
1238000000
1239000000
0
40000
80000
120000

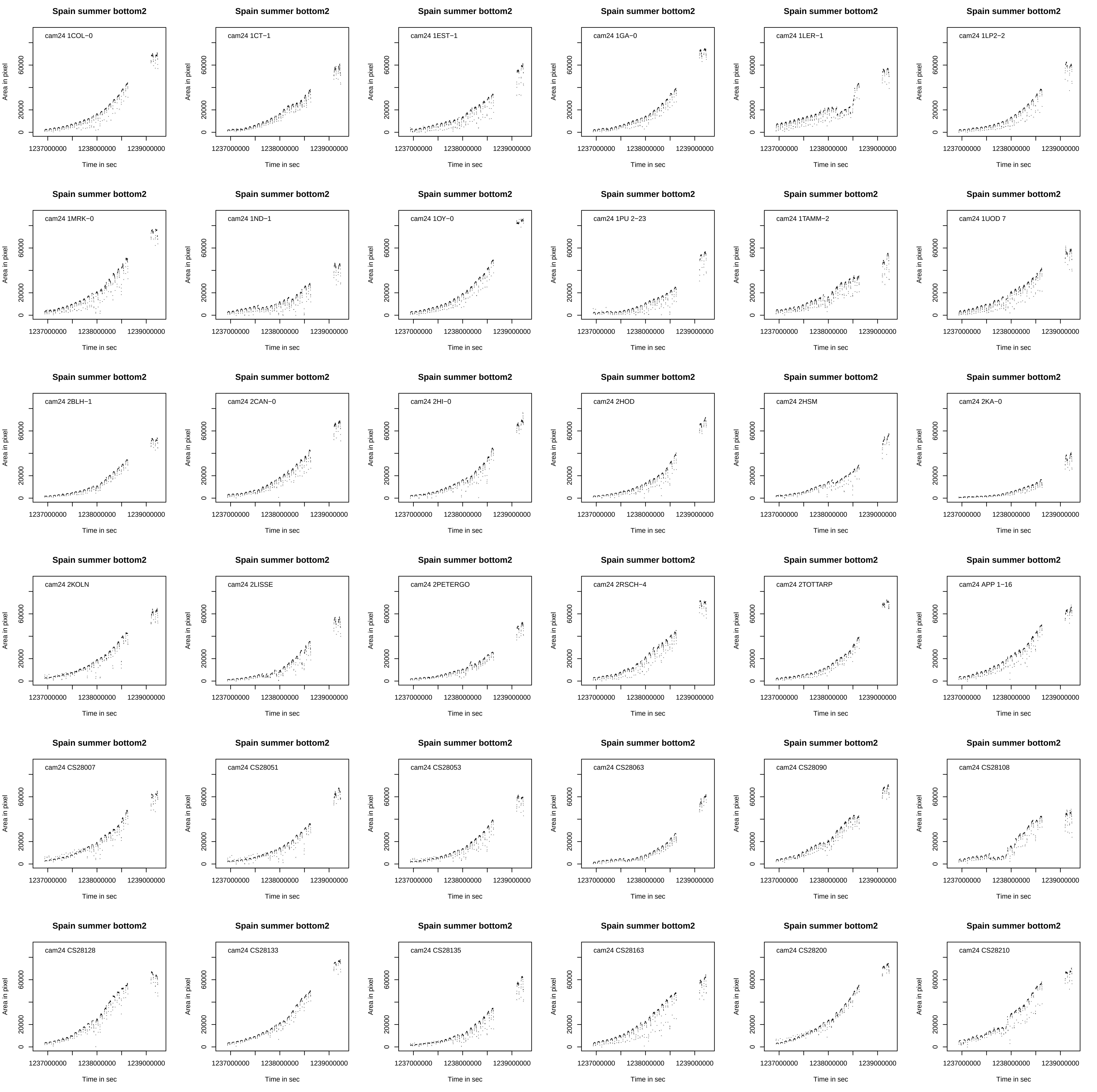
Spain summer bottom2
cam24 1COL−0
cam24 1CT−1
cam24 1EST−1
cam24 1GA−0
cam24 1LER−1
cam24 1LP2−2
cam24 1MRK−0
cam24 1ND−1
cam24 1OY−0
cam24 1PU 2−23
cam24 1TAMM−2
cam24 1UOD 7
cam24 2BLH−1
cam24 2CAN−0
cam24 2HI−0
cam24 2HOD
cam24 2HSM
cam24 2KA−0
cam24 2KOLN
cam24 2LISSE
cam24 2PETERGO
cam24 2RSCH−4
cam24 2TOTTARP
cam24 APP 1−16
cam24 CS28007
cam24 CS28051
cam24 CS28053
cam24 CS28063
cam24 CS28090
cam24 CS28108
cam24 CS28128
cam24 CS28133
cam24 CS28135
cam24 CS28163
cam24 CS28200
cam24 CS28210
Area in pixel
Time in sec
0
20000
60000
1237000000
1238000000
1239000000

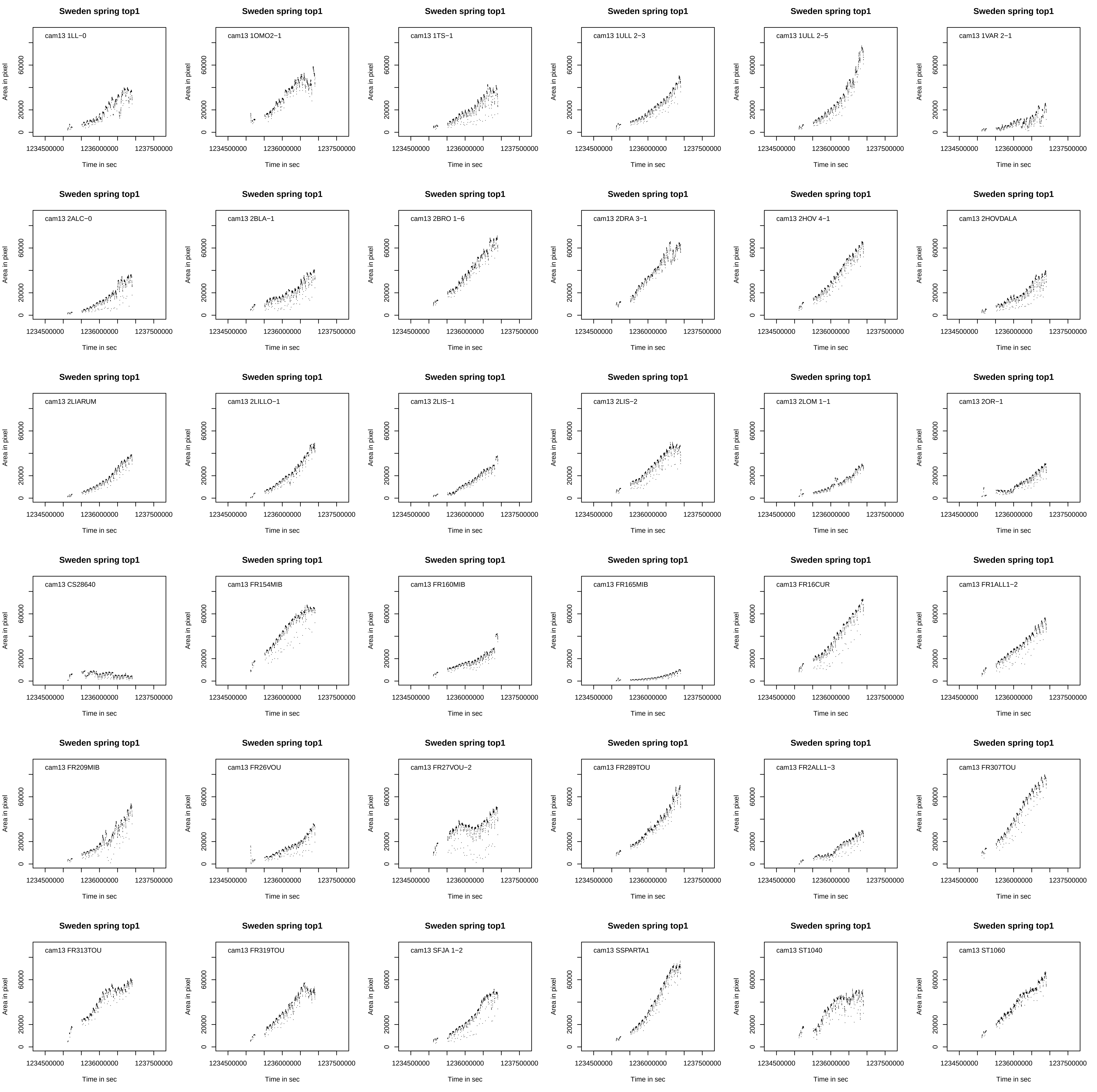
Sweden spring top1
cam13 1LL−0
Sweden spring top1
cam13 1OMO2−1
Sweden spring top1
cam13 1TS−1
Sweden spring top1
cam13 1ULL 2−3
Sweden spring top1
cam13 1ULL 2−5
Sweden spring top1
cam13 1VAR 2−1
Sweden spring top1
cam13 2ALC−0
Sweden spring top1
cam13 2BLA−1
Sweden spring top1
cam13 2BRO 1−6
Sweden spring top1
cam13 2DRA 3−1
Sweden spring top1
cam13 2HOV 4−1
Sweden spring top1
cam13 2HOVDALA
Sweden spring top1
cam13 2LIARUM
Sweden spring top1
cam13 2LILLO−1
Sweden spring top1
cam13 2LIS−1
Sweden spring top1
cam13 2LIS−2
Sweden spring top1
cam13 2LOM 1−1
Sweden spring top1
cam13 2OR−1
Sweden spring top1
cam13 CS28640
Sweden spring top1
cam13 FR154MIB
Sweden spring top1
cam13 FR160MIB
Sweden spring top1
cam13 FR165MIB
Sweden spring top1
cam13 FR16CUR
Sweden spring top1
cam13 FR1ALL1−2
Sweden spring top1
cam13 FR209MIB
Sweden spring top1
cam13 FR26VOU
Sweden spring top1
cam13 FR27VOU−2
Sweden spring top1
cam13 FR289TOU
Sweden spring top1
cam13 FR2ALL1−3
Sweden spring top1
cam13 FR307TOU
Sweden spring top1
cam13 FR313TOU
Sweden spring top1
cam13 FR319TOU
Sweden spring top1
cam13 SFJA 1−2
Sweden spring top1
cam13 SSPARTA1
Sweden spring top1
cam13 ST1040
Sweden spring top1
cam13 ST1060
Area in pixel
0
20000
60000
Time in sec
1234500000
1236000000
1237500000

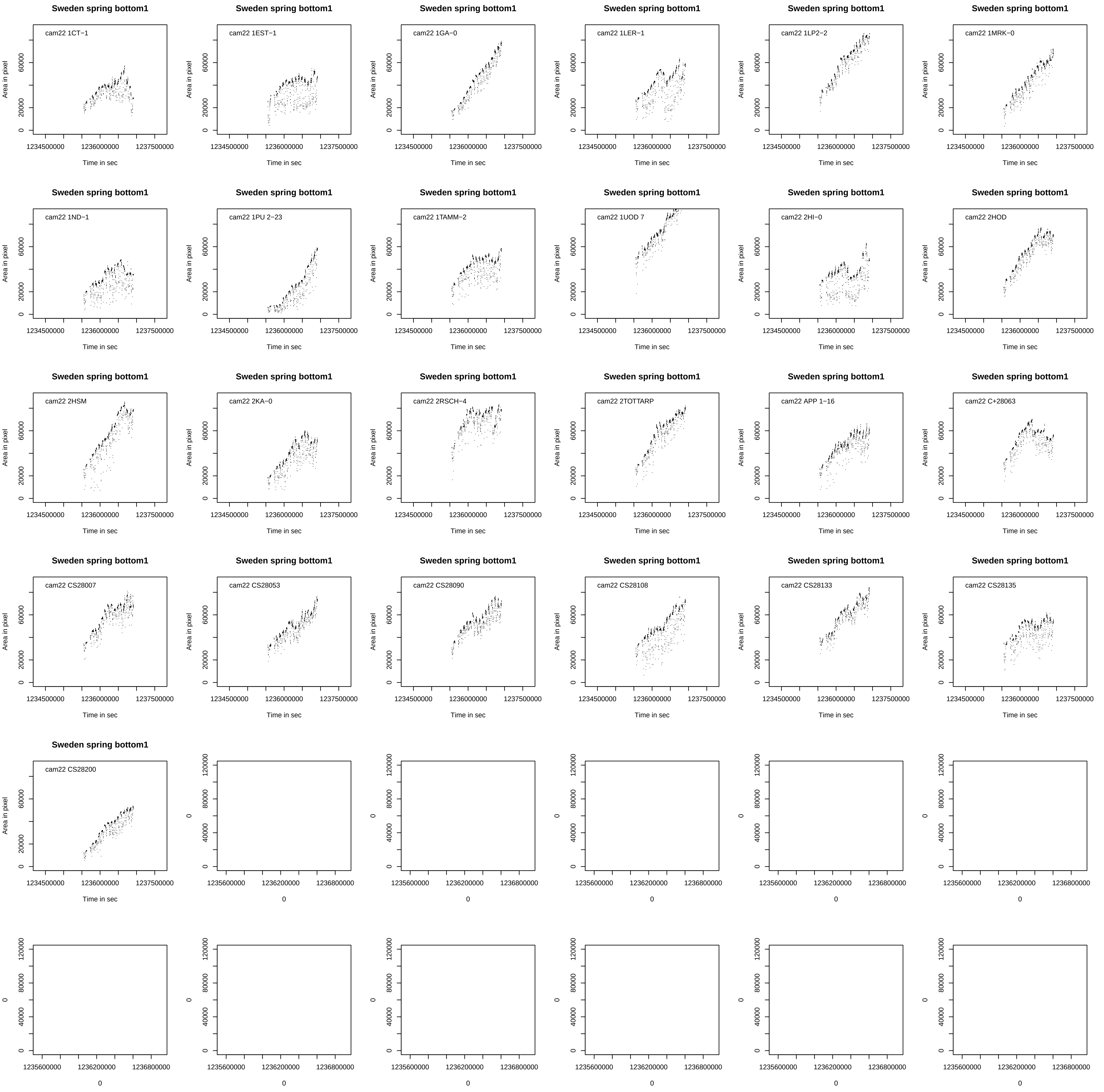

Sweden spring bottom1
cam22 1CT−1
cam22 1EST−1
cam22 1GA−0
cam22 1LER−1
cam22 1LP2−2
cam22 1MRK−0
cam22 1ND−1
cam22 1PU 2−23
cam22 1TAMM−2
cam22 1UOD 7
cam22 2HI−0
cam22 2HOD
cam22 2HSM
cam22 2KA−0
cam22 2RSCH−4
cam22 2TOTTARP
cam22 APP 1−16
cam22 C+28063
cam22 CS28007
cam22 CS28053
cam22 CS28090
cam22 CS28108
cam22 CS28133
cam22 CS28135
cam22 CS28200
Area in pixel
Time in sec
0
20000
60000
1234500000
1236000000
1237500000
40000
80000
120000
1235600000
1236200000
1236800000

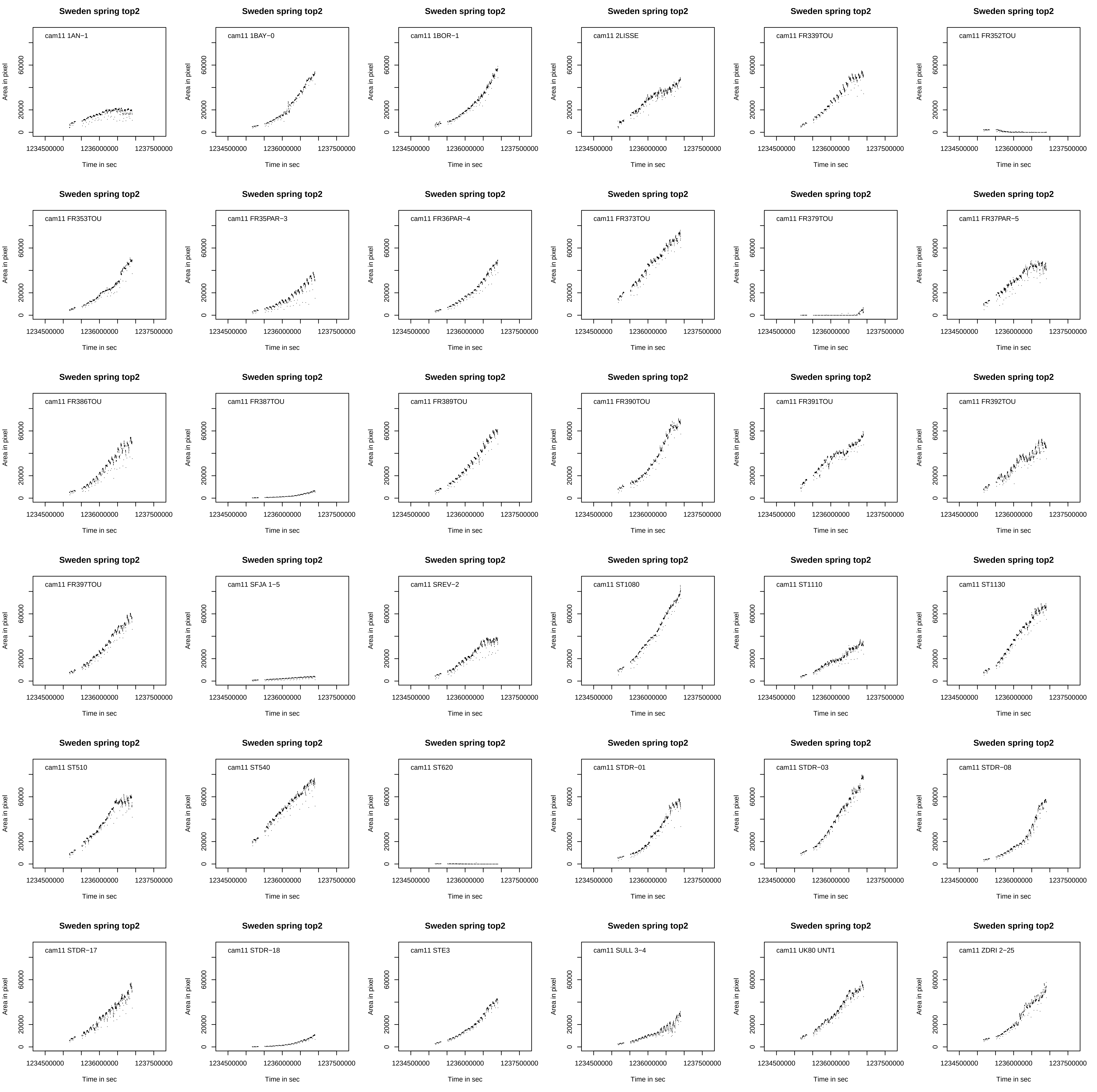
Sweden spring top2
cam11 1AN−1
Sweden spring top2
cam11 1BAY−0
Sweden spring top2
cam11 1BOR−1
Sweden spring top2
cam11 2LISSE
Sweden spring top2
cam11 FR339TOU
Sweden spring top2
cam11 FR352TOU
Sweden spring top2
cam11 FR353TOU
Sweden spring top2
cam11 FR35PAR−3
Sweden spring top2
cam11 FR36PAR−4
Sweden spring top2
cam11 FR373TOU
Sweden spring top2
cam11 FR379TOU
Sweden spring top2
cam11 FR37PAR−5
Sweden spring top2
cam11 FR386TOU
Sweden spring top2
cam11 FR387TOU
Sweden spring top2
cam11 FR389TOU
Sweden spring top2
cam11 FR390TOU
Sweden spring top2
cam11 FR391TOU
Sweden spring top2
cam11 FR392TOU
Sweden spring top2
cam11 FR397TOU
Sweden spring top2
cam11 SFJA 1−5
Sweden spring top2
cam11 SREV−2
Sweden spring top2
cam11 ST1080
Sweden spring top2
cam11 ST1110
Sweden spring top2
cam11 ST1130
Sweden spring top2
cam11 ST510
Sweden spring top2
cam11 ST540
Sweden spring top2
cam11 ST620
Sweden spring top2
cam11 STDR−01
Sweden spring top2
cam11 STDR−03
Sweden spring top2
cam11 STDR−08
Sweden spring top2
cam11 STDR−17
Sweden spring top2
cam11 STDR−18
Sweden spring top2
cam11 STE3
Sweden spring top2
cam11 SULL 3−4
Sweden spring top2
cam11 UK80 UNT1
Sweden spring top2
cam11 ZDRI 2−25
Area in pixel
0
20000
60000
Time in sec
1234500000
1236000000
1237500000

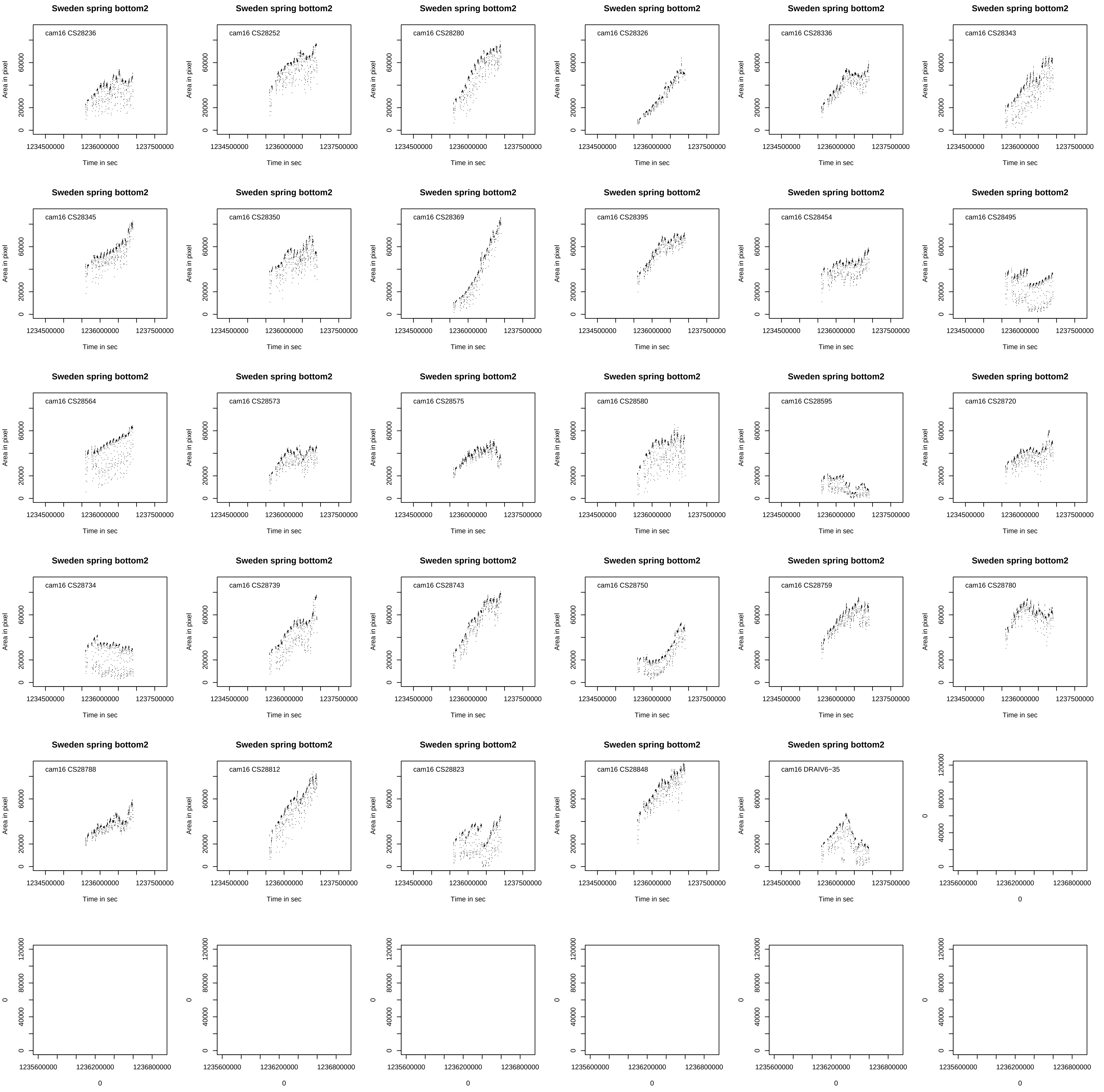
Sweden spring bottom2
cam16 CS28236
cam16 CS28252
cam16 CS28280
cam16 CS28326
cam16 CS28336
cam16 CS28343
cam16 CS28345
cam16 CS28350
cam16 CS28369
cam16 CS28395
cam16 CS28454
cam16 CS28495
cam16 CS28564
cam16 CS28573
cam16 CS28575
cam16 CS28580
cam16 CS28595
cam16 CS28720
cam16 CS28734
cam16 CS28739
cam16 CS28743
cam16 CS28750
cam16 CS28759
cam16 CS28780
cam16 CS28788
cam16 CS28812
cam16 CS28823
cam16 CS28848
cam16 DRAIV6−35
Area in pixel
0
20000
60000
Time in sec
1234500000
1236000000
1237500000
0
40000
80000
120000
1235600000
1236200000
1236800000

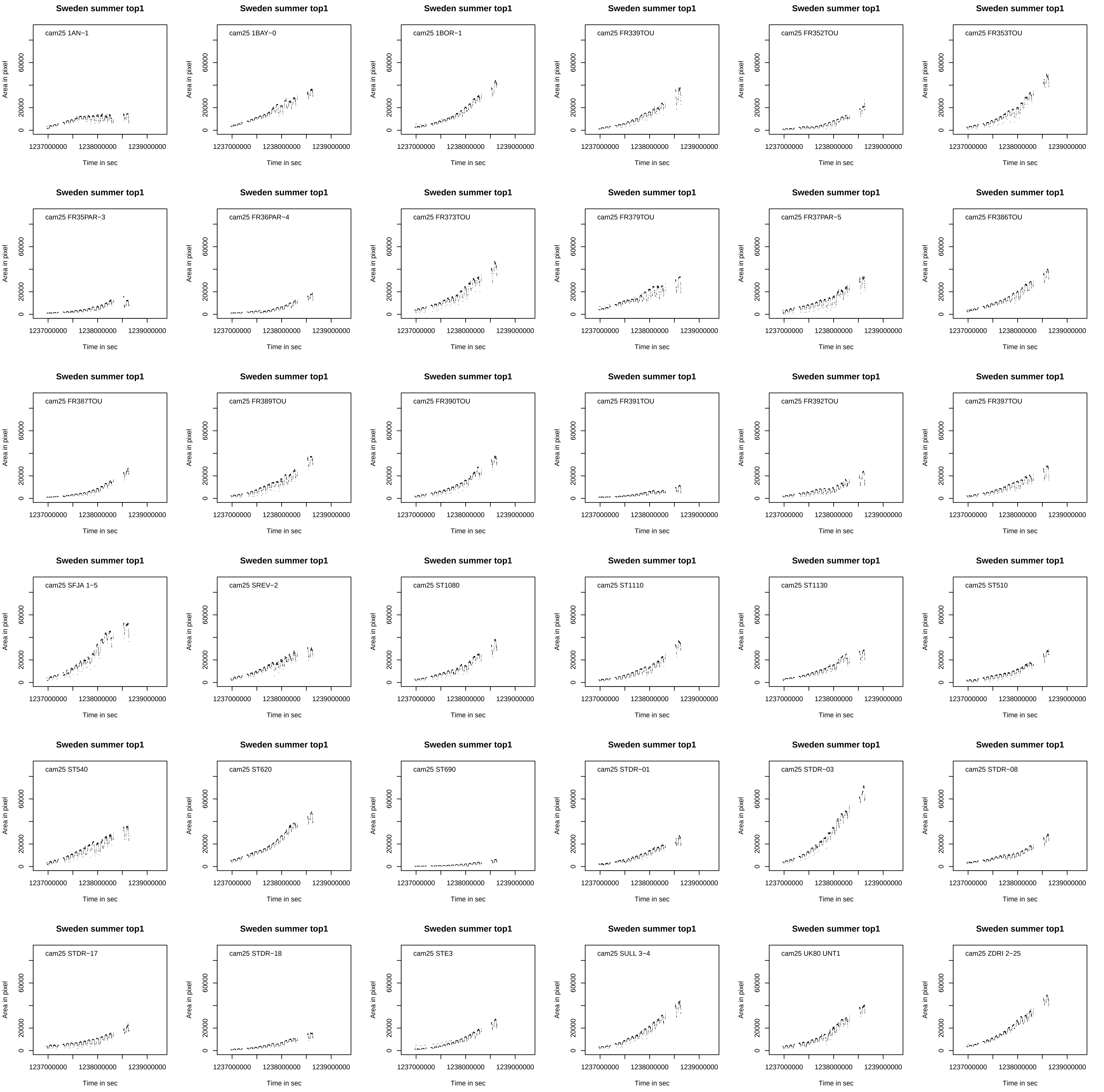
Sweden summer top1
cam25 1AN−1
Sweden summer top1
cam25 1BAY−0
Sweden summer top1
cam25 1BOR−1
Sweden summer top1
cam25 FR339TOU
Sweden summer top1
cam25 FR352TOU
Sweden summer top1
cam25 FR353TOU
Sweden summer top1
cam25 FR35PAR−3
Sweden summer top1
cam25 FR36PAR−4
Sweden summer top1
cam25 FR373TOU
Sweden summer top1
cam25 FR379TOU
Sweden summer top1
cam25 FR37PAR−5
Sweden summer top1
cam25 FR386TOU
Sweden summer top1
cam25 FR387TOU
Sweden summer top1
cam25 FR389TOU
Sweden summer top1
cam25 FR390TOU
Sweden summer top1
cam25 FR391TOU
Sweden summer top1
cam25 FR392TOU
Sweden summer top1
cam25 FR397TOU
Sweden summer top1
cam25 SFJA 1−5
Sweden summer top1
cam25 SREV−2
Sweden summer top1
cam25 ST1080
Sweden summer top1
cam25 ST1110
Sweden summer top1
cam25 ST1130
Sweden summer top1
cam25 ST510
Sweden summer top1
cam25 ST540
Sweden summer top1
cam25 ST620
Sweden summer top1
cam25 ST690
Sweden summer top1
cam25 STDR−01
Sweden summer top1
cam25 STDR−03
Sweden summer top1
cam25 STDR−08
Sweden summer top1
cam25 STDR−17
Sweden summer top1
cam25 STDR−18
Sweden summer top1
cam25 STE3
Sweden summer top1
cam25 SULL 3−4
Sweden summer top1
cam25 UK80 UNT1
Sweden summer top1
cam25 ZDRI 2−25
Area in pixel
0
20000
60000
Time in sec
1237000000
1238000000
1239000000

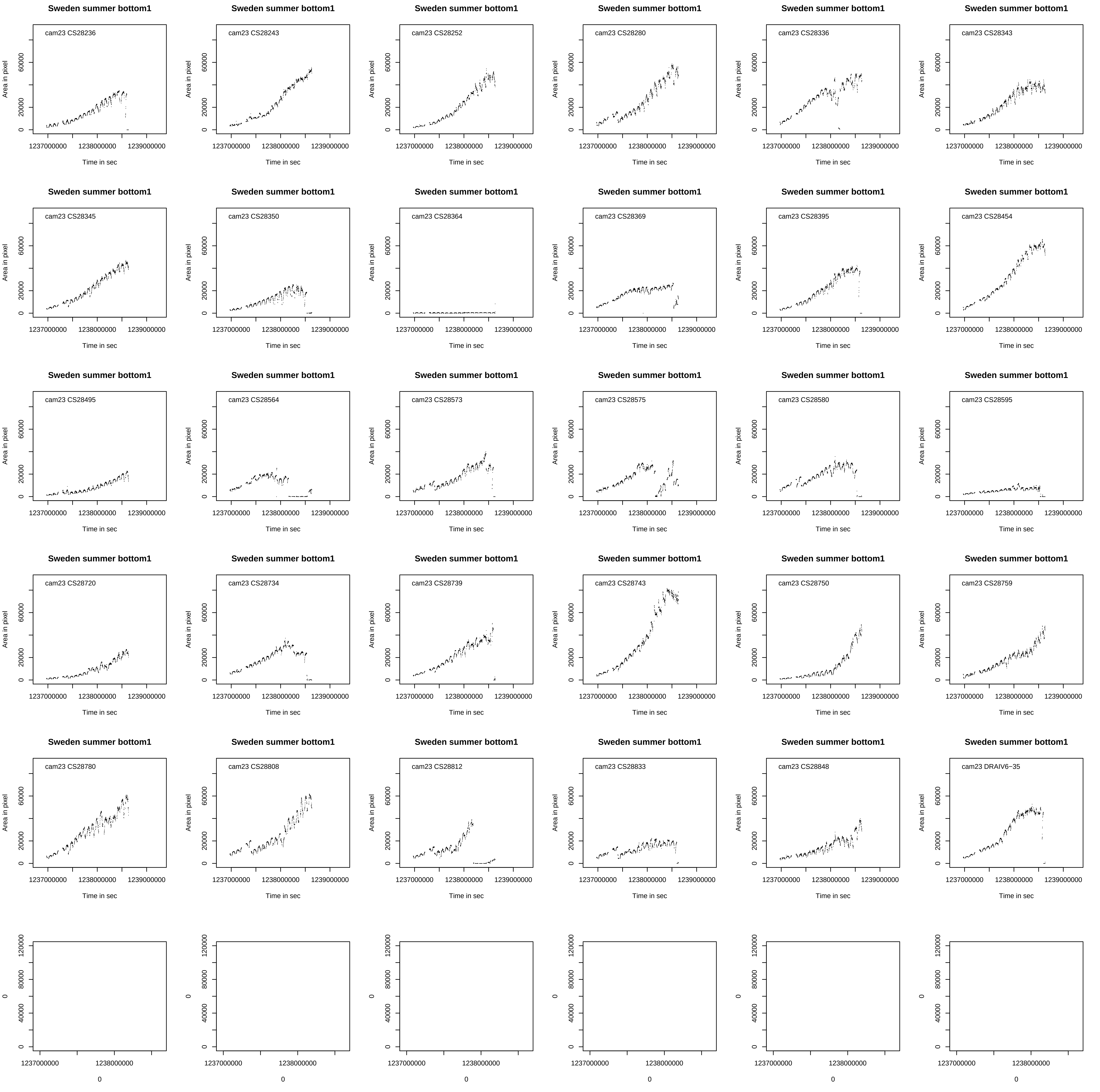
Sweden summer bottom1
cam23 CS28236
Sweden summer bottom1
cam23 CS28243
Sweden summer bottom1
cam23 CS28252
Sweden summer bottom1
cam23 CS28280
Sweden summer bottom1
cam23 CS28336
Sweden summer bottom1
cam23 CS28343
Sweden summer bottom1
cam23 CS28345
Sweden summer bottom1
cam23 CS28350
Sweden summer bottom1
cam23 CS28364
Sweden summer bottom1
cam23 CS28369
Sweden summer bottom1
cam23 CS28395
Sweden summer bottom1
cam23 CS28454
Sweden summer bottom1
cam23 CS28495
Sweden summer bottom1
cam23 CS28564
Sweden summer bottom1
cam23 CS28573
Sweden summer bottom1
cam23 CS28575
Sweden summer bottom1
cam23 CS28580
Sweden summer bottom1
cam23 CS28595
Sweden summer bottom1
cam23 CS28720
Sweden summer bottom1
cam23 CS28734
Sweden summer bottom1
cam23 CS28739
Sweden summer bottom1
cam23 CS28743
Sweden summer bottom1
cam23 CS28750
Sweden summer bottom1
cam23 CS28759
Sweden summer bottom1
cam23 CS28780
Sweden summer bottom1
cam23 CS28808
Sweden summer bottom1
cam23 CS28812
Sweden summer bottom1
cam23 CS28833
Sweden summer bottom1
cam23 CS28848
Sweden summer bottom1
cam23 DRAIV6−35
Area in pixel
0
20000
60000
1237000000
1238000000
1239000000
Time in sec

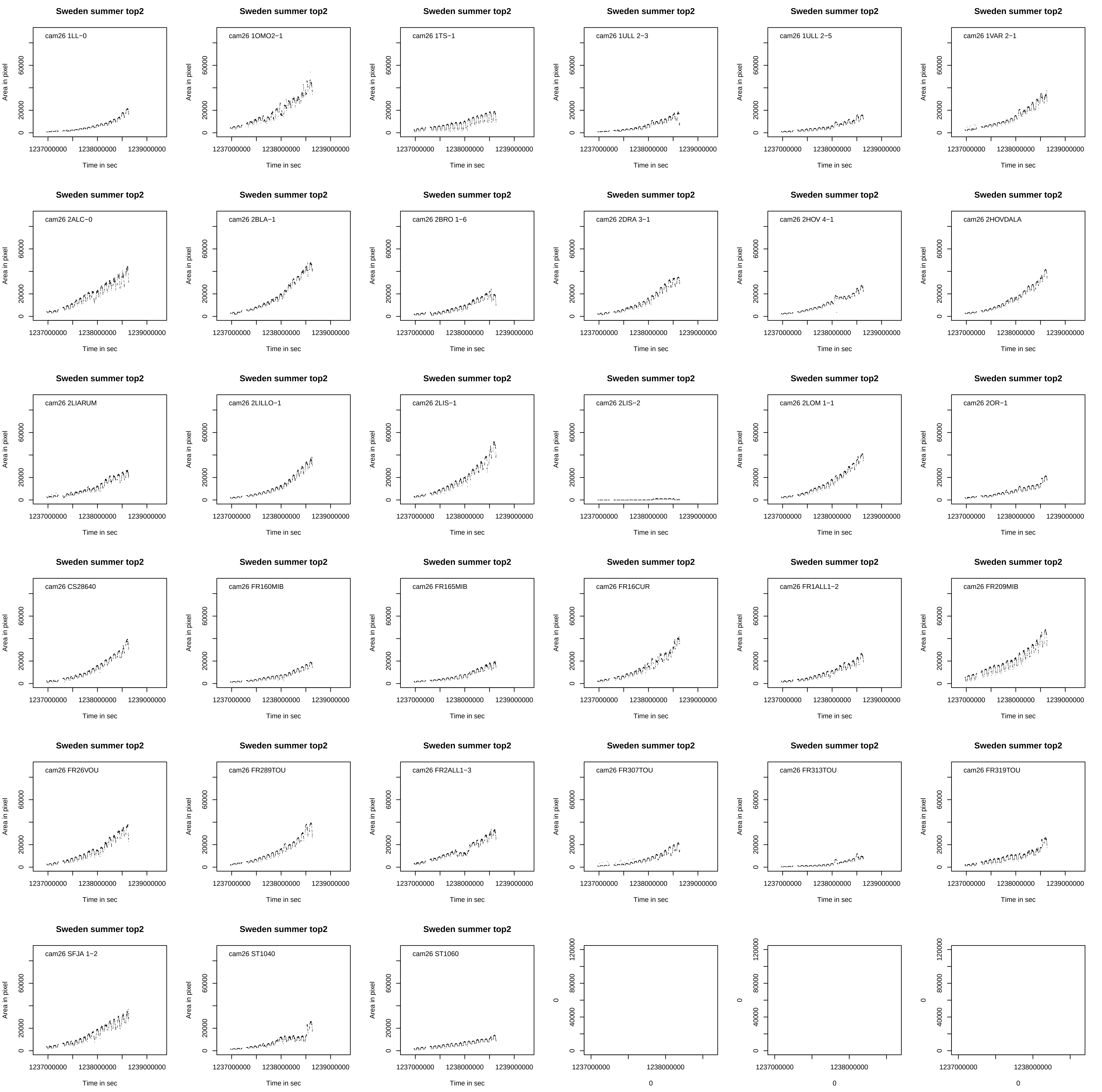
Sweden summer top2
cam26 1LL−0
cam26 1OMO2−1
cam26 1TS−1
cam26 1ULL 2−3
cam26 1ULL 2−5
cam26 1VAR 2−1
cam26 2ALC−0
cam26 2BLA−1
cam26 2BRO 1−6
cam26 2DRA 3−1
cam26 2HOV 4−1
cam26 2HOVDALA
cam26 2LIARUM
cam26 2LILLO−1
cam26 2LIS−1
cam26 2LIS−2
cam26 2LOM 1−1
cam26 2OR−1
cam26 CS28640
cam26 FR160MIB
cam26 FR165MIB
cam26 FR16CUR
cam26 FR1ALL1−2
cam26 FR209MIB
cam26 FR26VOU
cam26 FR289TOU
cam26 FR2ALL1−3
cam26 FR307TOU
cam26 FR313TOU
cam26 FR319TOU
cam26 SFJA 1−2
cam26 ST1040
cam26 ST1060
Area in pixel
0
20000
60000
Time in sec
1237000000
1238000000
1239000000
40000
80000
120000

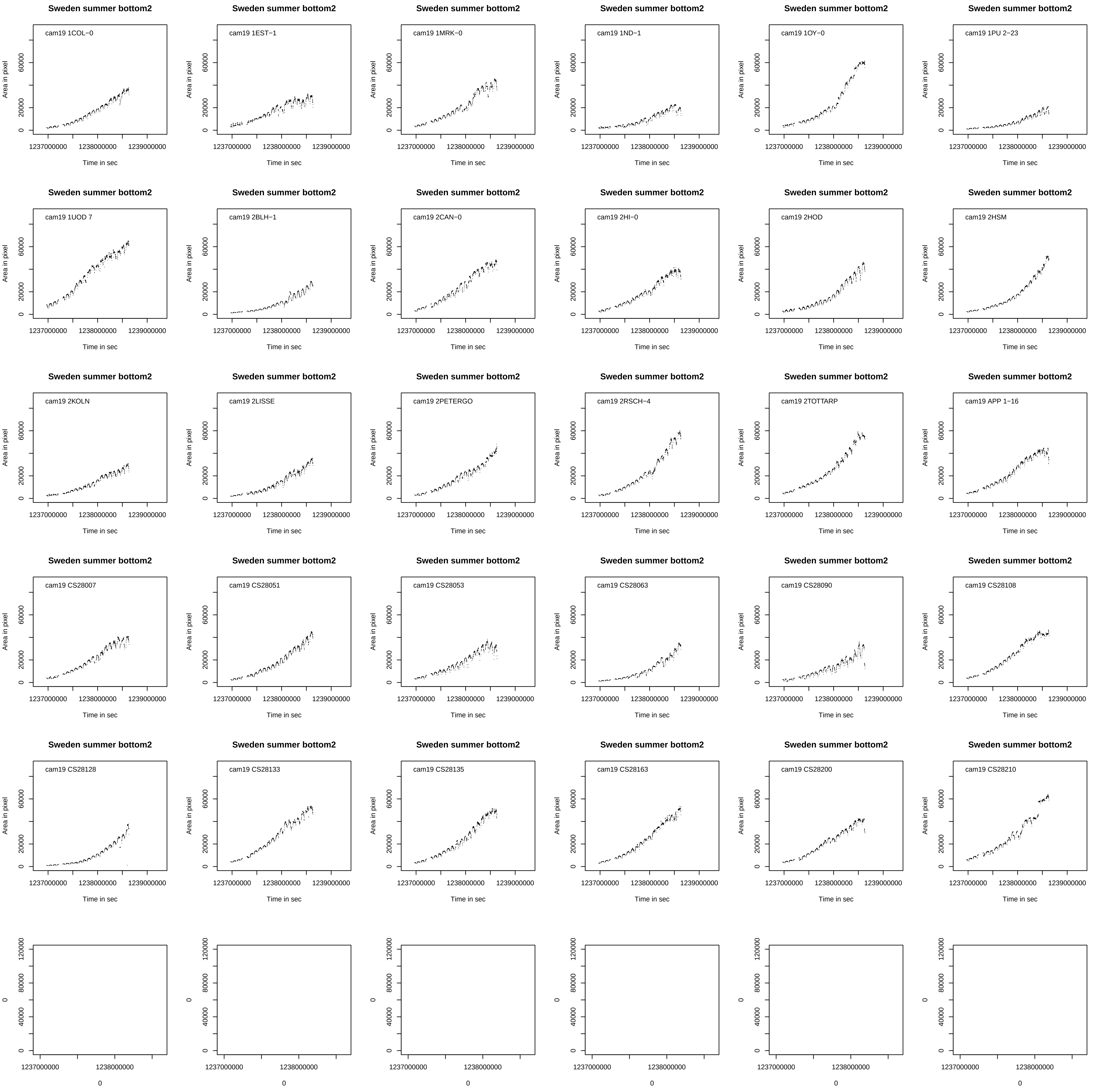

Sweden summer bottom2
cam19 1COL−0
cam19 1EST−1
cam19 1MRK−0
cam19 1ND−1
cam19 1OY−0
cam19 1PU 2−23
cam19 1UOD 7
cam19 2BLH−1
cam19 2CAN−0
cam19 2HI−0
cam19 2HOD
cam19 2HSM
cam19 2KOLN
cam19 2LISSE
cam19 2PETERGO
cam19 2RSCH−4
cam19 2TOTTARP
cam19 APP 1−16
cam19 CS28007
cam19 CS28051
cam19 CS28053
cam19 CS28063
cam19 CS28090
cam19 CS28108
cam19 CS28128
cam19 CS28133
cam19 CS28135
cam19 CS28163
cam19 CS28200
cam19 CS28210
Area in pixel
0
20000
60000
Time in sec
1237000000
1238000000
1239000000
0
40000
80000
120000
