# Supplementary material for: Natural Genetic Variation for Growth and Development Revealed by High-Throughput Phenotyping in Arabidopsis thaliana
Source: G3 (Bethesda). 2012 Jan 1;2(1):29–34. doi: 10.1534/g3.111.001487 (PMC3276187; doi:10.1534/g3.111.001487)
Supplement: Supporting Information [file supp_2.1.29_FileS3.pdf]

GWA Mapping of Rosette Area

RA was measured every 20min from the beginning of the day when plants reach the 1.05 stage to the end of the day when plants reach the 1.10 stage, for Spain spring and Spain summer conditions. Growth was scaled across the developmental frame, and RA were obtained at 200 evenly spaced developmental time points.

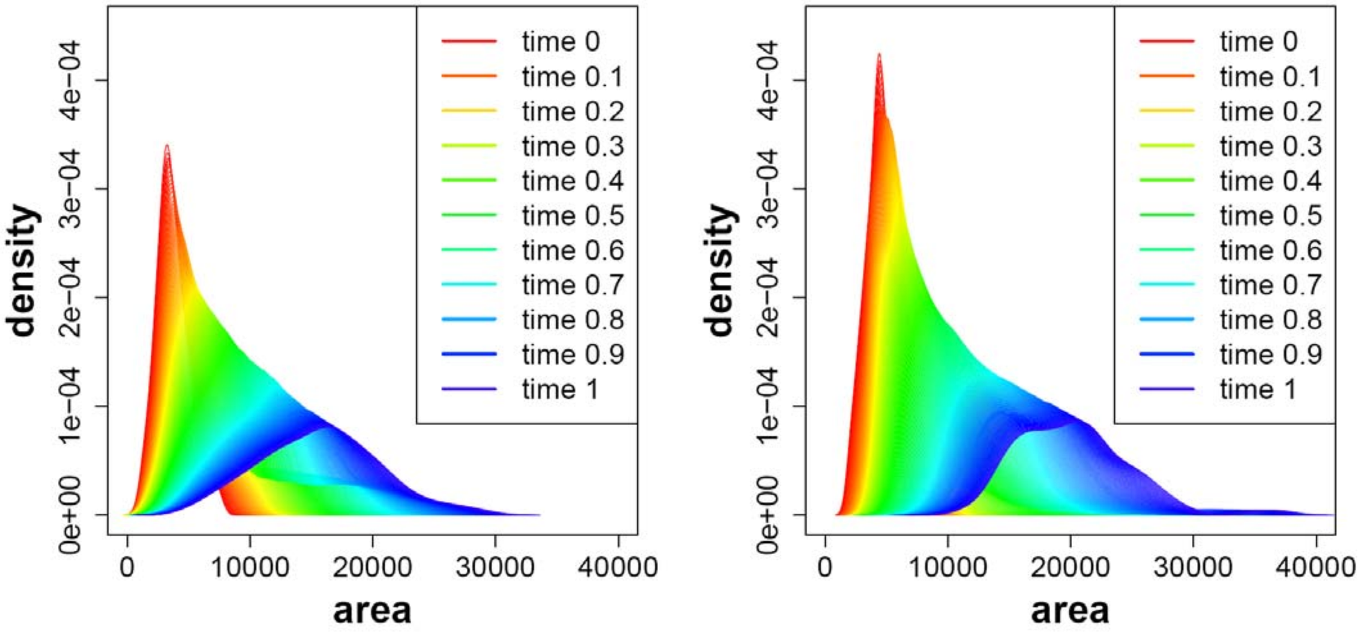

The density distributions of RA across relative developmental time, for Spain spring (left) and Spain summer (right) conditions. RA under both growth conditions generally show a normal distribution across development, implying the polygenic nature of a complex trait.

For GWA analysis, 145,652 (Spain spring) and 147,627 (Spain summer) SNPs with minor allele frequency > 0.1 were scanned by a single locus model using EMMA (Kang et al. 2008), at each developmental time point. The model includes a kinship matrix as covariance of additive random genetic effect, and flat as a fixed covariate.

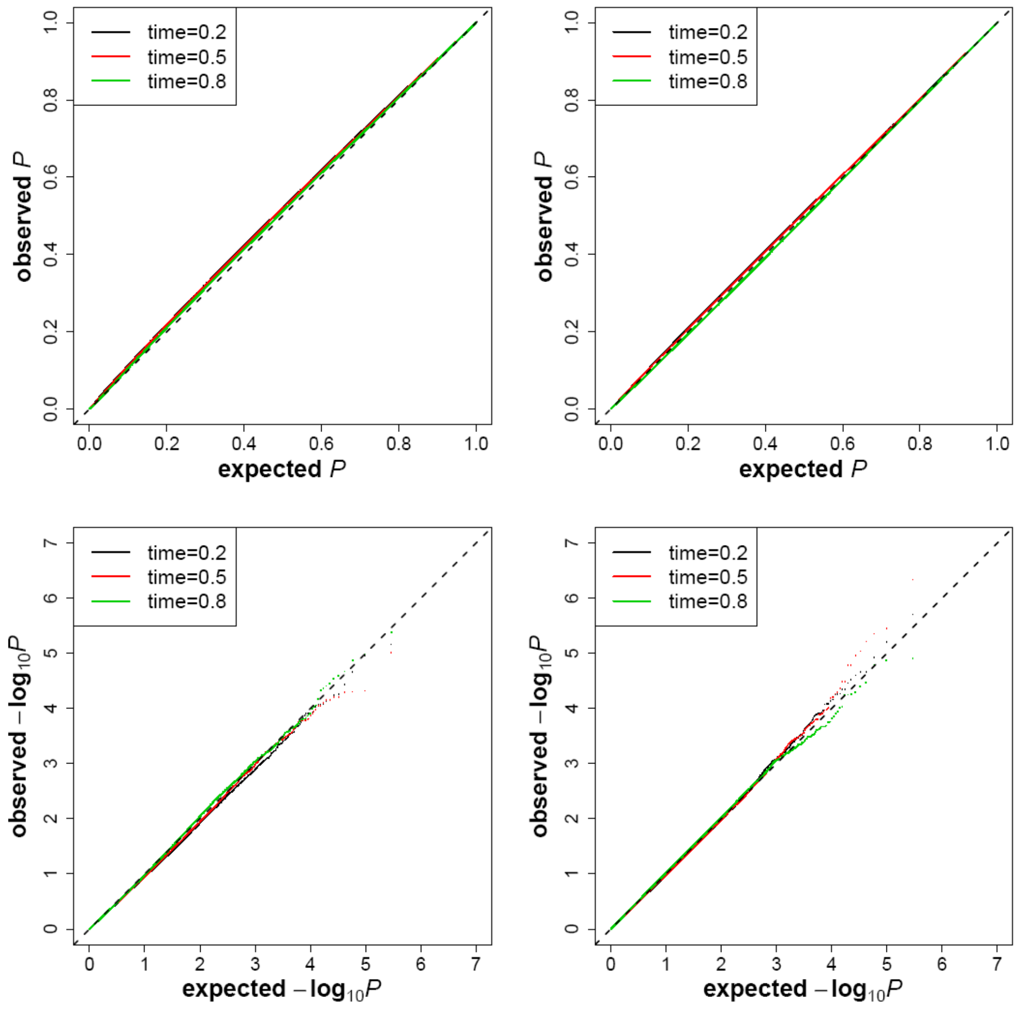

Few enrichment of significant associations in real data. In the upper panel, the observed p-value were plotted against the expected p-value, whereas in the lower panel, the observed $-\log_{10}$ p-value were plotted against the expected $-\log_{10}$ p-value, for Spain spring (left panel) and Spain summer (right panel), at relatively developmental time 0.2 (black solid line), 0.5 (red solid line) and 0.8 (green solid line). Dashed line represents diagonal line.

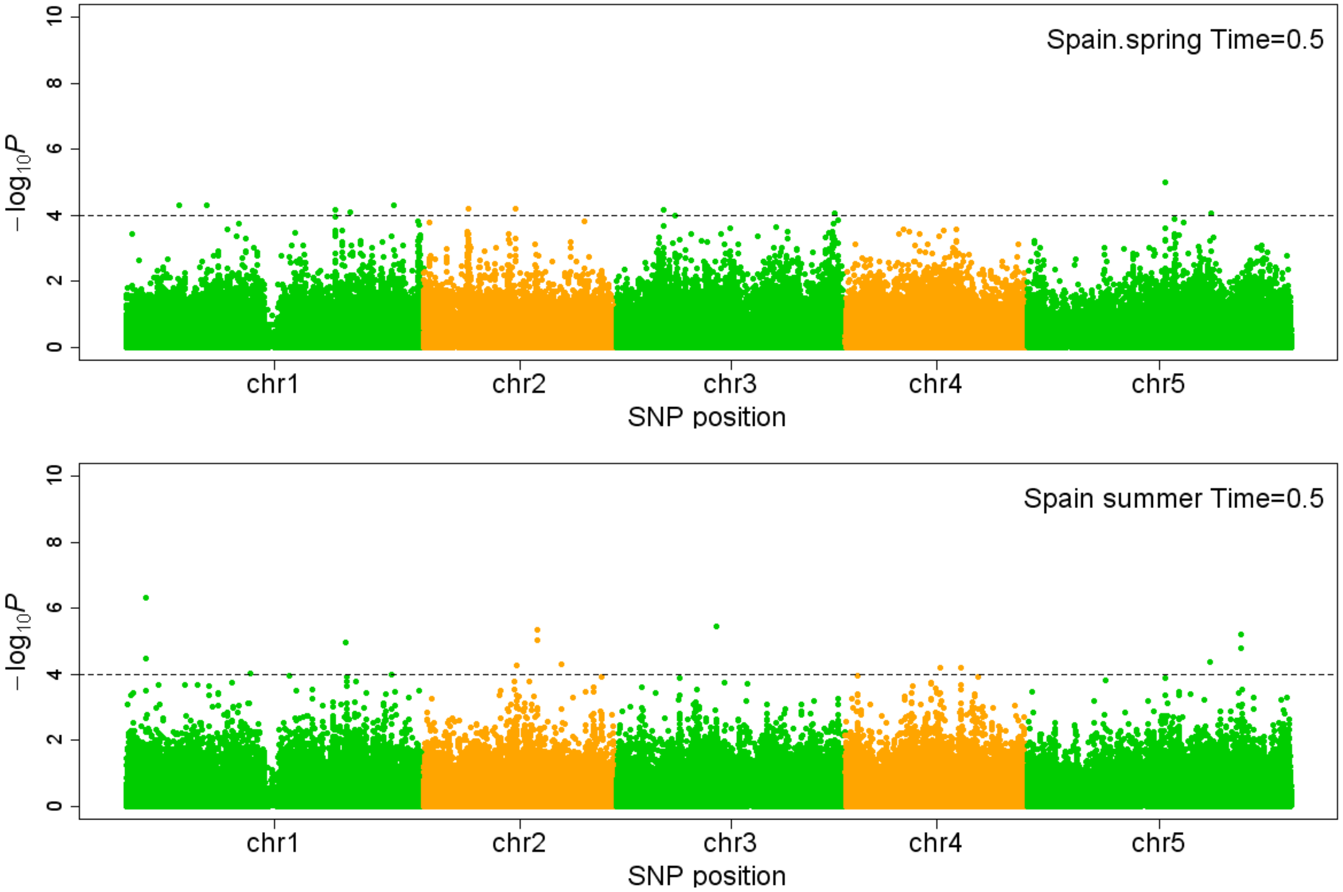

At relative developmental time of 0.5, the GWA mapping result in Spain spring (upper panel) and Spain summer (lower panel). The $-\log_{10}$ p-value were plotted against chromosomal position.

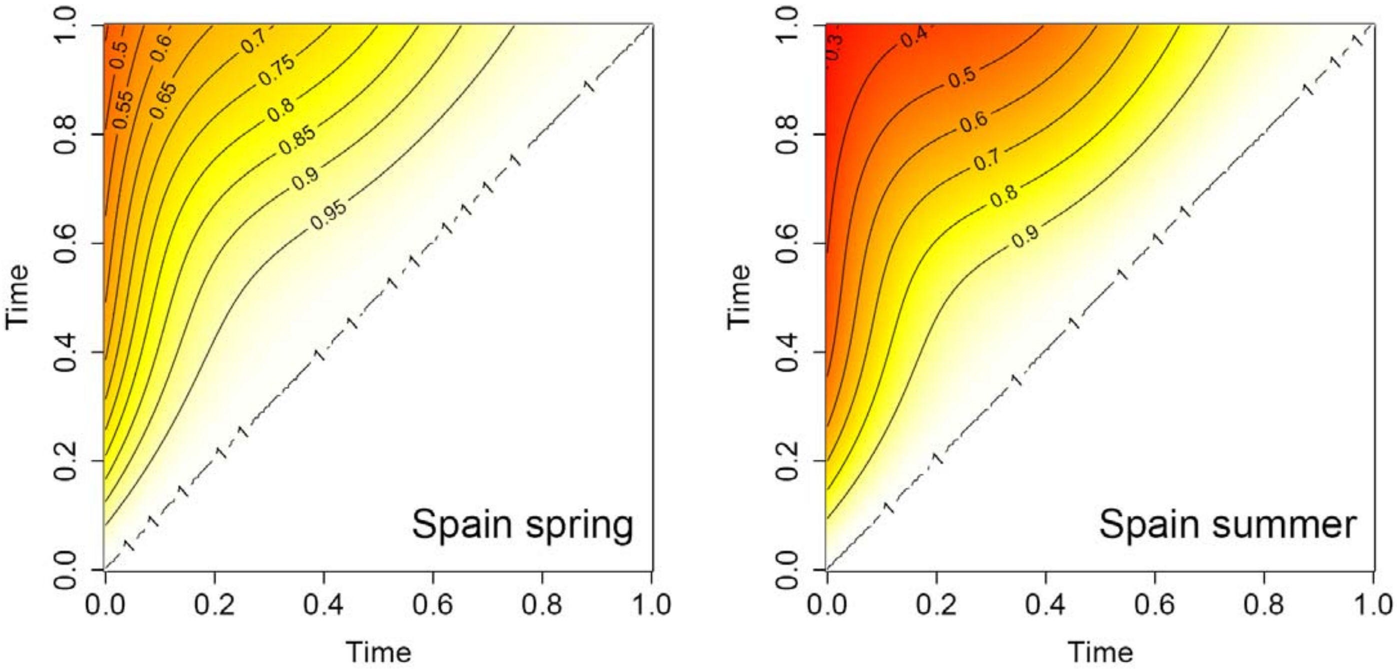

Correlation of association profiles (-$\log_{10}$ p-value) across developmental time within Spain spring (left) and within Spain summer (right). Across the developmental time frame, the association profiles within Spain spring were more correlated than those within Spain summer (mean r = 0.87 and 0.78, respectively). Under both environments, the mid- and late-growth stages were more similar with each other than with early-growth stage.

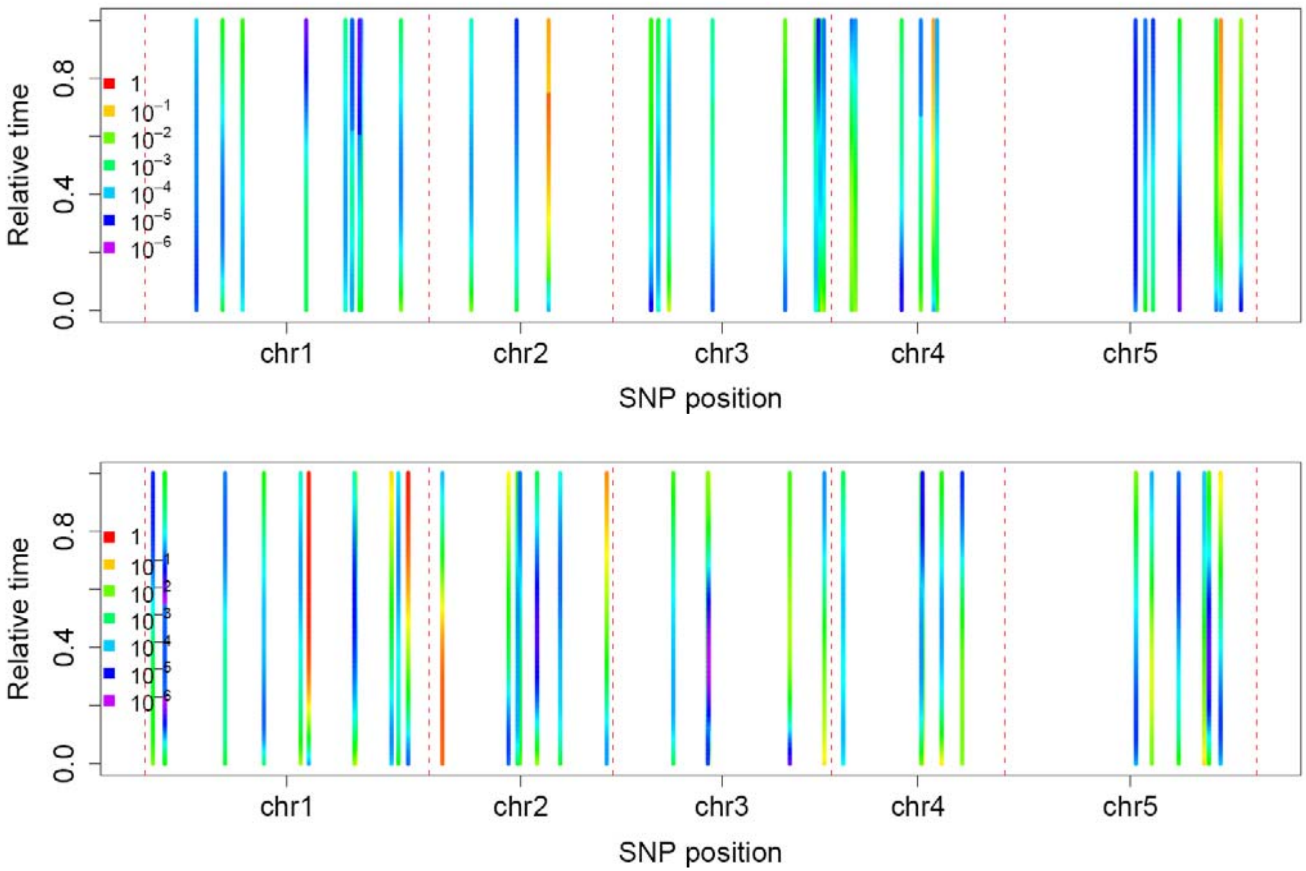

A total of 35 and 36 unlinked ($r^2 > 0.2$) association peaks were found for Spain spring (upper panel) and Spain summer (lower panel), respectively, at $p < 10^{-4}$ for at least one time point across development. Some of these QTL have a consistent effect throughout development, while others act at early, later, or at intermediate stages of development.

For each time point, Best Linear Unbiased Predictors (BLUPs) for accessions across flat within environment were used for estimation of QTL effect (Tian et al. 2011). BLUPs were obtained using QTLRel (Cheng et al. 2010).

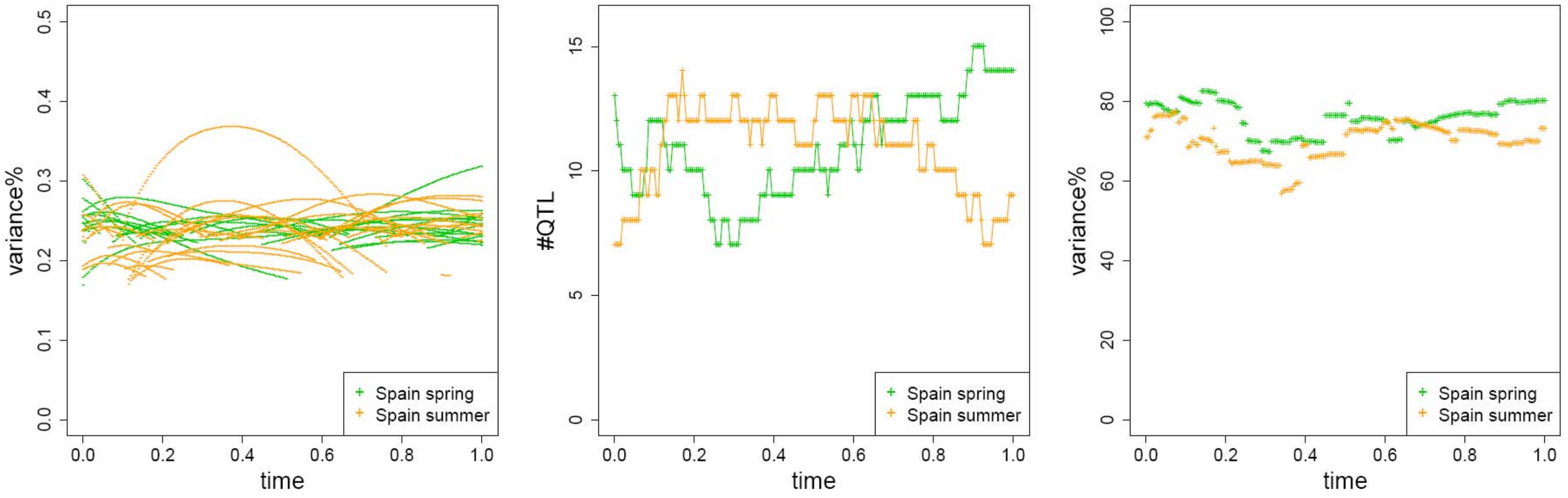

A single peak explained 17%-37% trait variation in a single locus model, with a median of 24% (left). At specific developmental time points, 7-15 and 7-14 SNPs were significant at $p < 10^{-4}$ for Spain spring and Spain summer condition, respectively (middle). Together these SNPs explained 67%-82% trait variation in Spain spring and 57%-77% trait variation in Spain summer, using a multi-locus model (right). The multi-locus model includes additive, unlinked SNPs ($r^2 > 0.2$) with marginal $-\log_{10} p > 4$. Green points represent Spain spring, orange points represent Spain summer.

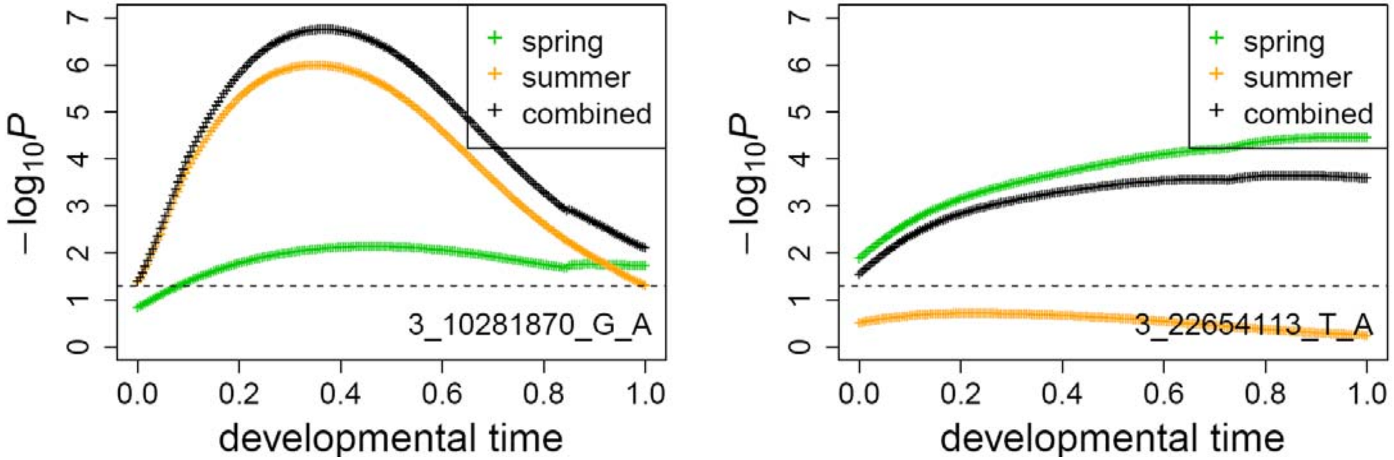

Few association peak overlapped between the two environments. SNP 3_10281870_G_A (left, MAF=0.28 in spring, 0.22 in summer) is the only SNP that is significant at $p < 10^{-4}$ in one environment, while still significant at $p < 0.01$ in the other. Most of the associated SNPs look like this SNP 3_22654113_T_A (right, MAF=0.21 in spring, 0.10 in summer). The $-\log_{10}$ p-value were plotted against relative developmental time. Combined: the $-\log_{10}$ p-value obtained by Fisher's combined probability test, which treats Spring and Summer as independent cohorts.

References

Cheng, R., J.E. Lim, K.E. Samocha, G. Sokoloff, M. Abney, A.D. Skol, and A.A. Palmer. 2010. Genome-wide association studies and the problem of relatedness among advanced intercross lines and other highly recombinant populations. Genetics 185: 1033-1044.

Kang, H.M., N.A. Zaitlen, C.M. Wade, A. Kirby, D. Heckerman, M.J. Daly, and E. Eskin. 2008. Efficient control of population structure in model organism association mapping. Genetics 178: 1709-1723.

Tian, F., P.J. Bradbury, P.J. Brown, H. Hung, Q. Sun, S. Flint-Garcia, T.R. Rocheford, M.D. McMullen, J.B. Holland, and E.S. Buckler. 2011. Genome-wide association study of leaf architecture in the maize nested association mapping population. Nat Genet 43: 159-162.
